# The inferior fronto-occipital fasciculus: bridging phylogeny, ontogeny and functional anatomy

**DOI:** 10.1093/brain/awaf055

**Published:** 2025-02-11

**Authors:** Davide Giampiccolo, Guillaume Herbet, Hugues Duffau

**Affiliations:** Department of Clinical and Experimental Epilepsy, UCL Queen Square Institute of Neurology, University College London, London WC1N 3BG, UK; Victor Horsley Department of Neurosurgery, National Hospital for Neurology and Neurosurgery, Queen Square, London WC1N 3BG, UK; Department of Neurosurgery, Institute of Neuroscience, Cleveland Clinic London, London SW1X 7HY, UK; Department of Neurosurgery, Gui de Chauliac Hospital, Montpellier University Medical Center, Montpellier 34295, France; Institut Universitaire de France, Paris 75005, France; Department of Medicine, University of Montpellier, Montpellier 34090, France; Praxiling Laboratory, UMR 5267, CNRS, Paul Valéry University, Montpellier 34090, France; Department of Neurosurgery, Gui de Chauliac Hospital, Montpellier University Medical Center, Montpellier 34295, France; Institute of Functional Genomics, University of Montpellier, INSERM, CNRS, Montpellier 34000, France

**Keywords:** brain mapping, IFOF, white matter stimulation, cognition, semantic control, social-semantic control

## Abstract

The inferior-fronto-occipital fasciculus (IFOF) is a long-range white matter tract that connects the prefrontal cortex with parietal, posterior temporal and occipital cortices. First identified in the 19th century through the pioneering studies of Mayo and Meynert using blunt dissection, its anatomy and function remain contentious topics. Structurally, its projections are well documented in human blunt dissection and tractography literature, yet its existence has been questioned by tract-tracing studies in macaques. Functionally, while traditional results from direct white matter stimulation during awake surgery suggested a contribution to language, recent evidence from stimulation and lesion data may indicate a broader role in executive control, extending to attention, motor cognition, memory, reading, emotion recognition and theory of mind.

This review begins by examining anatomical evidence suggesting that the IFOF evolved in non-human primates to connect temporal and occipital cortices to prefrontal regions involved in context-dependent selection of visual features for action. We then integrate developmental, electrophysiological, functional and anatomical evidence for the human IFOF to propose it has a similar role in manipulation of visual features in our species—particularly when inhibition of overriding but task-irrelevant stimuli is required to prioritize a second, task-relevant stimulus.

Next, we introduce a graded model in which dorsal (orbitofrontal, superior and middle frontal to precuneal, angular and supero-occipital projections) and ventral (inferior frontal to posterotemporal, basal temporal and infero-occipital) projections of the IFOF support perceptual or conceptual control of visual representations for action, respectively. Leveraging this model, we address controversies in the current literature regarding language, motor cognition, attention and emotion under the unifying view of cognitive control. Finally, we discuss surgical implications for this model and its impact on predicting and preventing neurological deficits in neurosurgery.

## Teaser

Giampiccolo *et al.* review the anatomy and function of the inferior fronto-occipital fasciculus. They propose that it plays a role in visual cognitive control and has two distinct layers: a dorsal layer, which supports control based on perceptual features, and a ventral layer, which is engaged in control of conceptual content.

**See Hoven et al. (https://doi.org/10.1093/brain/awaf114) for a scientific commentary on this article.**

## Introduction

**Figure 1:**
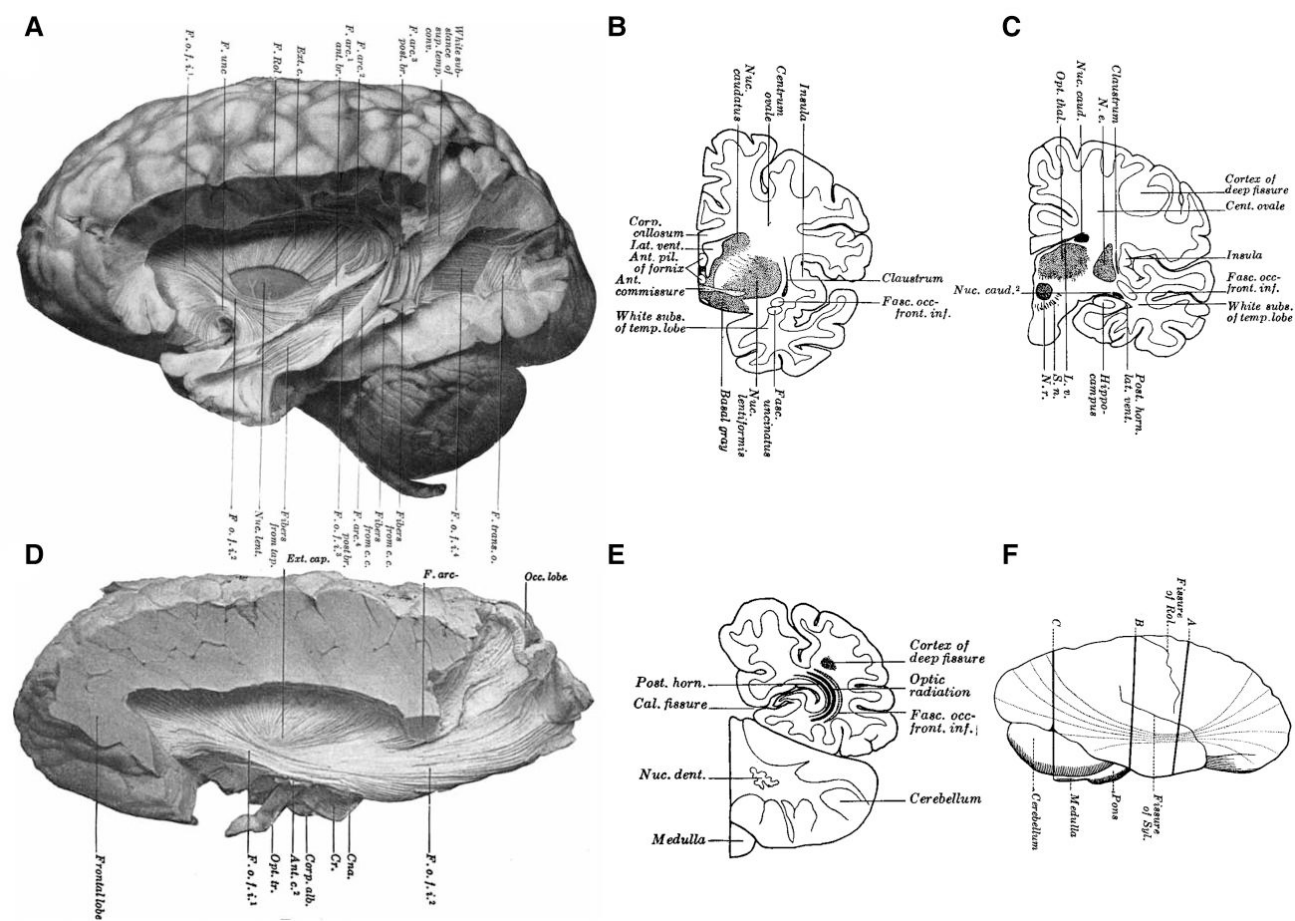
**Historical dissection of the inferior fronto-occipital fasciculus following Curran**. Dissection of the fasciculus occipito-frontalis inferior as described by Curran (modified from Curran, 1909).^3^ (**A**) 3D lateral view, showing the relationship of the fasciculus occipito-frontalis inferior (*f.o.f.i.*) with the uncinate fasciculus (*f. unc*) and the arcuate fasciculus (*f. arc*, distinguished in an anterior and posterior branch). (**B**) Sectional coronal view at the level of the temporal stem, with fasciculus occipito-frontalis inferior (*fasc.occ-front.inf.*) being located above the uncinate fasciculus (*fasc. uncinatus*). (**C**) Sectional coronal view at the level of the temporal lobe, with the fasciculus occipito-frontalis inferior (*fasc.occ-front.inf.*) located above the lateral ventricle, within the sagittal stratum. (**D**) 3D lateral view, the fasciculus occipito-frontalis inferior (*f.o.f.i.*) is shown along its whole extension having removed adjacent crossing fibres. (**E**) Sectional coronal view of the occipital lobe, with the fasciculus occipito-frontalis inferior (*fasc.occ-front.inf.*) being located in the superficial layer of the sagittal stratum, superficial to the optic radiation. (**F**) Depiction of fasciculus occipito-frontalis inferior's projections between frontal and parieto-occipital regions.

The inferior fronto-occipital fasciculus (IFOF) is one of the longest association white matter pathways in humans. The existence of projections passing above the temporal lateral ventricle, the temporal stem and the limen insulae to the frontal lobe was initially depicted in 1823 in engravings by Herbert Mayo^1^ and described by Theodor Meynert using blunt dissection.^2^ Further anatomical evidence from these connections came from Curran in 1909,^3^ who replicated Meynert's results describing ‘a large associating bundle of fibres uniting, as its name indicates, the occipital with the frontal lobe. It also contains fibres, which join the frontal lobe with the posterior part of the temporal and parietal lobes’ and first used the name ‘fasciculus occipito-frontalis inferior’ to distinguish this from the superior fronto-occipital fasciculus reported by Onufrowicz in patients with agenesis of the corpus callosum (Fig. 1).^4,5^

The emergence of white matter stimulation during awake neurosurgery in the second millennium brought about a renewed interest in the inferior fronto-occipital fasciculus. In 2005, it became apparent that stimulation along its trajectory in the sagittal stratum, at temporo-occipital level, insula or frontal lobe could cause semantic paraphasia during a picture naming task.^6^ Importantly, subsequent stimulation data showed that semantic deficits were not restricted to verbal content: impaired non-verbal semantic processing at the same subcortical locations was present when using the Pyramid and Palm Tree test (PPTT),^7^ a visual task where a black-and-white picture is associated with second, semantically-related picture. This causal evidence from stimulation was supported by converging results from neuroimaging studies in healthy participants and stroke patients,^8,9^ with the IFOF proposed to underlie semantic processing,^10^ and particularly semantic representations within language.^8,11^

Despite this, the anatomy and function of the IFOF are far from being clarified. On one hand, not only the extension of its cortical projections, but its nature as a monosynaptic cortico-cortical connection has been historically^5^ and recently^12^ debated as its existence was not supported by tract-tracing in non-human primates^5,13,14^ and may have stemmed from artefacts produced by blunt dissection and tractography.^5,12^ On the other hand, while some consider it the pre-eminent connection for semantic language processing, its major occipital and inferior temporal projections—rather than superior temporal—make it poorly suited to support acoustic representation of ‘features, segments (phonemes), syllabic structure, phonological word forms, grammatical features and semantic information’ as predicted by the dual stream model of Hickok and Poeppel.^15^ This discrepancy has to be considered together with recent evidence that this pathway may not be specific to language as its interruption through stimulation or resection can also impact motor cognition, attention, mentalizing and memory.^16-23^ Hence, the anatomy and function of the IFOF are considered a matter of controversy.

This review addresses this issue. In the first section, we focus on the anatomy of the IFOF in non-human primates. We discuss literature, including tract-tracing studies in primates, that supports the existence of the IFOF along the simian lineage. This aims to resolve the debate surrounding the existence of the IFOF and posits not only that this pathway evolved early through primate phylogeny, but also that its function may precede the development of language. In the second part, we focus on the human IFOF, first discussing its development, anatomy and lateralization, then proposing a role for this fascicle in manipulation of visual features for goal-directed behaviour according to its layers. In the third section, we discuss how this pathway may support shared executive processes within different functional domains, thus offering a unifying view to disputes on its role in language, motor cognition, attention, memory and emotional control. In the last section, we discuss the relevance this has for neurosurgery.

This review utilizes complementary anatomical and electrophysiological techniques to investigate anatomy and function of the IFOF in both the left and right hemispheres, leveraging, whenever possible, causal data from brain stimulation. The literature reviewed (928 articles) and criteria for selection can be found in the online Supplementary material.

Post-mortem invasive tracing methods using viruses^24^ or isotope techniques^14,25^ in non-human primates are the most precise method for disentangling white matter connections in animal models. These have proven invaluable in establishing the cortical connections among primate brain areas, which may represent network archetypes for human connections.^26^ However, it is important to consider that there are sizable differences between non-human and human primates and different (or novel)^27^ connections may support the more sophisticated behavioural repertoire unique of humans.^28^ Therefore, to accurately discuss the anatomy of the IFOF in humans, it is necessary to take the anatomo-functional divergence of humans from non-human primates into account and to adopt other techniques to account for this. Blunt dissection has been used since the 19th century to identify white matter pathways *ex vivo* by peeling away grey and white matter with a spatula to expose the relevant connections.^29^ More recently, tractography has been used to unravel white matter connections *in vivo* by extrapolating constrained diffusion of water within axons to indirectly calculate their trajectory.^30^ However, both blunt dissection and tractography have limitations, including the challenge of precise white matter pathway segmentation in conditions of crossing fibres,^31^ which can result in false positive^31^ and false negative^32,33^ trajectories biasing fibre evaluation. The use of different tractography algorithms can also result in substantial differences in reconstruction. Moreover, the variability and the disagreement in the identified projections of the IFOF adds to the uncertainty surrounding its anatomical connections.

Intraoperative direct electrical stimulation (DES) can provide invaluable information on the functional roles of white matter connections. This method involves applying a probe to electrically stimulate and causally disrupt cortical or white matter structures in awake patients while they are performing a task,^34,35^ and it is the gold standard for functional preservation during neurosurgery.^34^ In addition, cortico-cortical evoked potentials (CCEPs),^36^ an electrophysiological method that allows recording of electrical activity generated from a distant, connected cortex, can provide complementary information about connectivity between cortices and therefore map how the IFOF may be connected.

## Nomenclature

Besides the disagreement in the literature regarding the IFOF's anatomy, the nomenclature used to refer to this pathway is inconsistent. One issue may have stemmed from contention in the literature over the superior fronto-occipital fasciculus^3,4^ and its relation to the IFOF. Reappraisal of the former's connections to the caudate nucleus and thalamus, rather than a cortico-cortical connection, may have cast doubts on the monosynaptic nature of the latter.^5,37,38^ Another discrepancy has possibly followed different ways of describing this connection in the macaque tract-tracing literature—extreme capsule fascicle^14,39^—and human tractography literature—IFOF.^6,40^ These definitions put emphasis on different projections passing through the sagittal stratum: for the extreme capsule fascicle, emphasis is placed on fronto-temporal connections,^39,41^ while the term IFOF instead highlights inferior frontal and orbitofrontal connections to the parietal and occipital lobe.^3,40^ However, these may represent two layers of a continuous pathway, as proposed anatomically^42^—and functionally with direct white matter stimulation of both posterior temporal and parieto-occipital projections causing similar disruptions in semantics.^43^

In this review, we will discuss temporo-frontal (including the so-called extreme capsule fascicle) and parieto-occipito-frontal projections together as two layers of the IFOF, aligning with the terminology in the neurosurgical literature that considers both temporal and parieto-occipital components as segments of the same pathway.^44-48^

## Phylogeny of the IFOF in primates

### Tract-tracing evidence for the IFOF in macaques

While a description of the human IFOF has been established since Meynert and Curran, the evidence for a non-human primate equivalent is controversial. This issue has been highlighted by Schmahmann and Pandya in 2007, who could not reveal this connection using tract-tracing and wrote ‘There is no convincing support in the experimental literature for connections between inferior occipital regions and the orbitofrontal or ventrolateral prefrontal cortices, and our own observations in the monkey also do not document an ‘inferior FOF.’^5^ Instead, a direct temporo-frontal connection converging into the extreme capsule was identified by Petrides and Pandya in 1988,^14^ which was named the extreme capsule fascicle. This connection showed cortical terminations in area Ts2, TE3 and Tpt in the superior temporal gyrus and widespread connections with area 6, 8, 9, area 46 and 47 in the frontal lobe, with a pattern confirmed by the authors in their subsequent work.^13,39,49,50^ Further temporo-frontal connections were described by other groups: injections in the middle temporal area (MT) revealed projections to the ventrolateral prefrontal cortex and the frontal eye field.^51,52^ Therefore, the absence of occipito-frontal connections in macaques reported by the authors was used to imply the absence of an IFOF in non-human primates.

**Figure 2:**
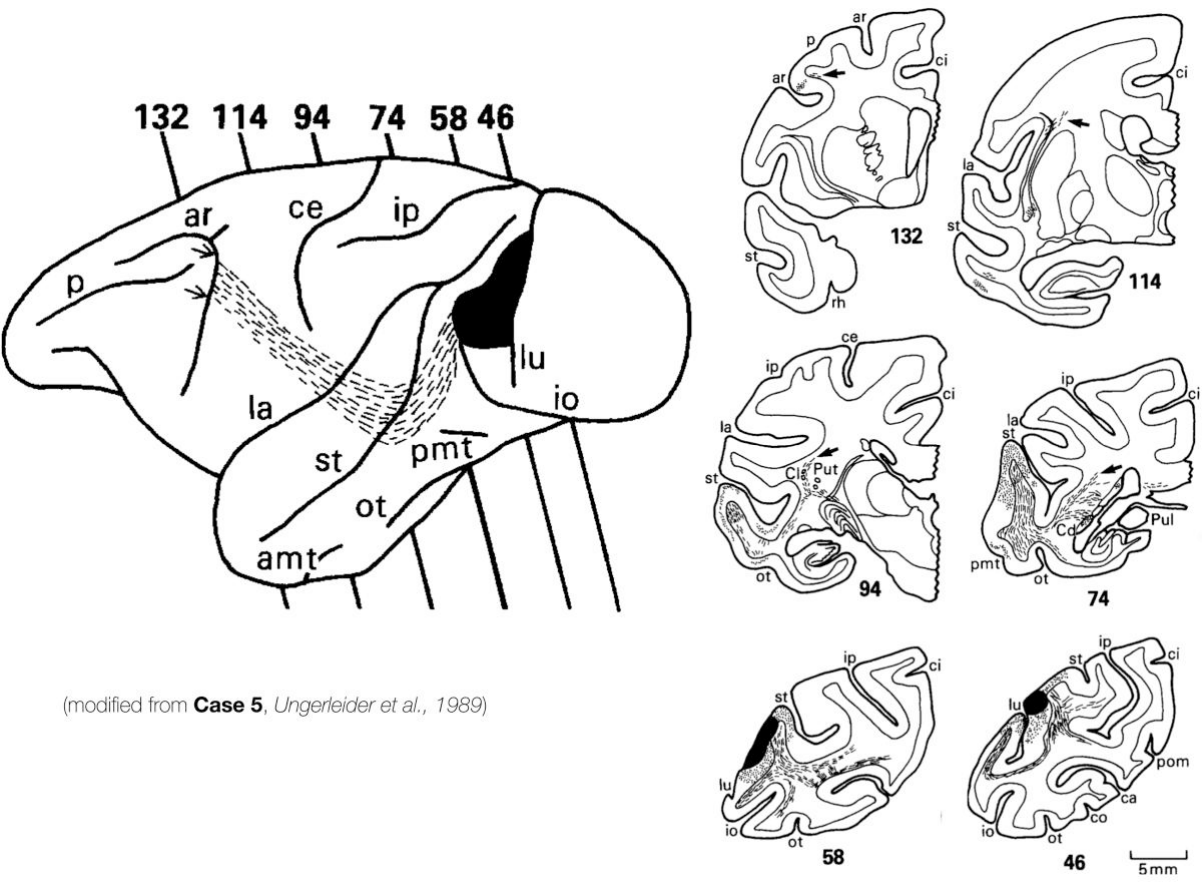
**Subcortical trajectory of occipito-frontal projections connecting V4 and the arcuate sulcus evidenced by tract-tracing in macaques**. A connection linking the arcuate sulcus and area V4 is evidenced with tract-tracing in macaques (modified from Ungerleider *et al*.^25^). This connection passes ‘beneath the superior temporal sulcus for approximately 5 mm, then turned dorsally to course through the claustrum and continued forward to their targets on the anterior bank of the arcuate sulcus’, resembling the inferior fronto-occipital fasciculus (IFOF) in the macaque. amt = anterior middle temporal sulcus; ar = arcuate sulcus; ce = central sulcus; io = inferior occipital sulcus; ip = intraparietal sulcus; la = lateral sulcus; lu = lunate sulcus; m.orb = medial orbital sulcus; ot = occipitotemporal sulcus; p = principal sulcus; pmt = posterior middle temporal sulcus; st = superior temporal sulcus.

Other groups, however, have shown occipito-frontal connections compatible with a macaque IFOF using tract-tracing with autoradiography.^25,53-55^ Occipito-frontal connections passing through the temporal stem and extreme capsule were described by Ungerleider in 1989: in Case 5 a connection projecting from V4 to the frontal eye field (FEF) is shown passing through the extreme capsule (‘Labelled fibers projecting to prefrontal cortex followed a path from the injection site that coursed rostrally beneath the superior temporal sulcus for approximately 5 mm, then turned dorsally to course through the claustrum and continued forward to their targets on the anterior bank of the arcuate sulcus’),^25^ with a clear resemblance to the IFOF (Fig. 2). Under this premise, projections resembling the IFOF using tract-tracing by Ungerleider may oppose claims that this pathway does not exist in macaques, and consequently that this likely does not exist in humans.

### Putative cortical terminations for the IFOF in non-human primates

Further evidence for monosynaptic connections between macaque regions that may resemble homologous cortical terminations of the human IFOF has been reported in tract-tracing literature.^51,56,57^ A larger occipital and basal temporal (V3d/V3v, V4, OT, TEO) anterograde projection to area 8/FEF have since been shown with tract tracing by Gerbella and colleagues.^54^ Further, inferotemporal connections (TEpv, TEav, TE, TEO) to ventral prefrontal (area 45b) and orbitofrontal cortices (area 13, area 12r, area 11),^58-60^ precuneal (7 m) to ventral (area 46) and dorsal prefrontal (area 8, 9)^61^ and occipital (V2) to orbitofrontal^62,63^ have also been identified with tract-tracing, which may align with cortical terminations of the IFOF in humans.

Quantitative evaluation of injections in the marmoset may also support the existence of connectivity at cortical terminations of the IFOF in this species. Reciprocal connections between V4 and 47, V4 and 45 have been identified in a comprehensive atlas of injections using fluorescent retrograde tracers.^64,65^ The authors further highlighted that TEO is reciprocally connected with 47 and 45. Reciprocal connections have also been identified between area 8 and V4 and TEO, with area 11 being connected with V2 and area 19m projecting to area 8a.^65^ As such, evidence from tract-tracing in the macaque and marmoset shows a pattern of cortico-cortical connections, which may resemble those that have been proposed for the IFOF in the human literature. Nevertheless, there is, to date, only a limited number of tract-tracing studies that document the axonal trajectory of these connections, and therefore a role for alternative direct cortico-cortical connections must be considered.

**Figure 3:**
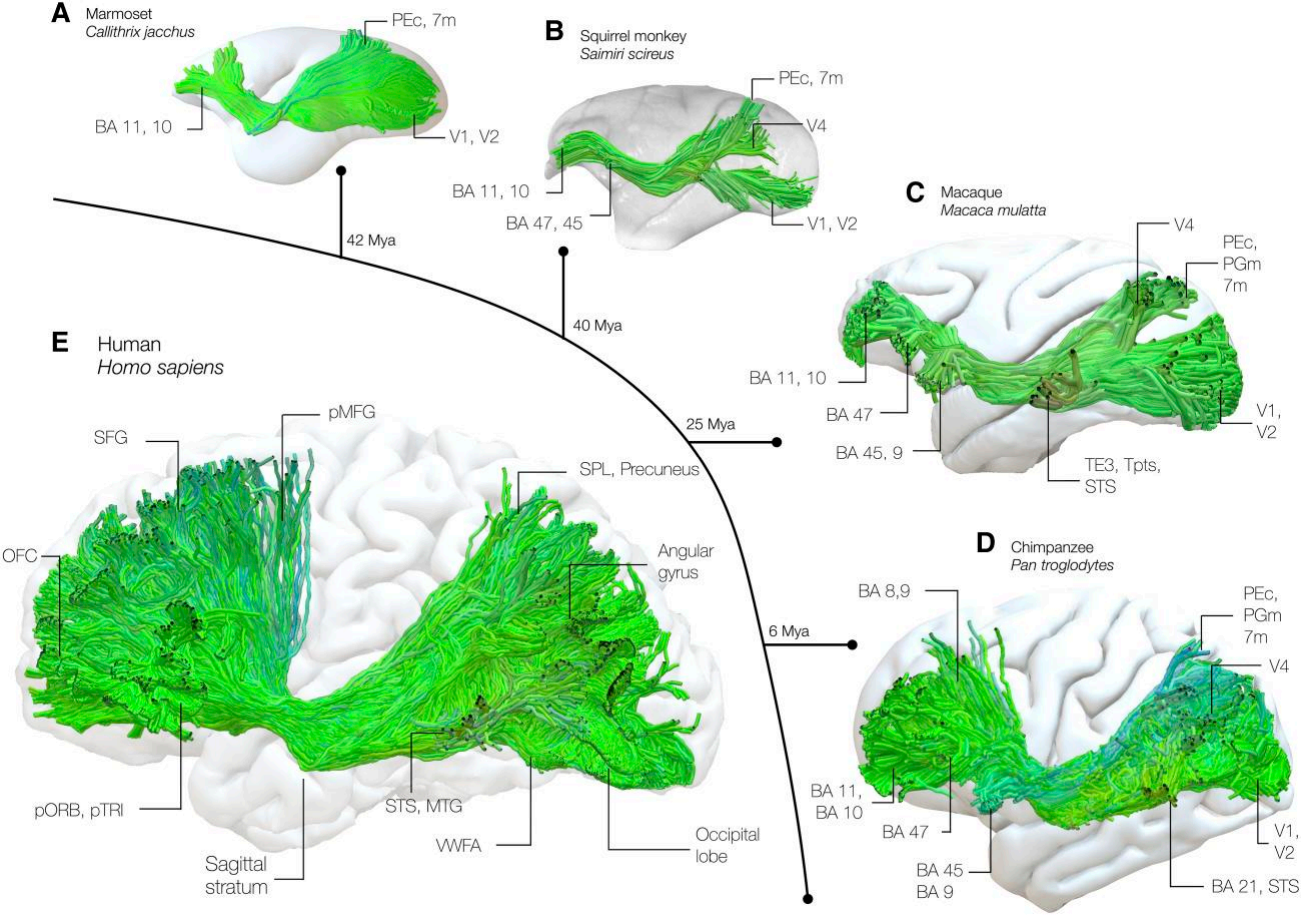
**Shared projections of the inferior fronto-occipital fasciculus along the primate lineage**. Homologues of the inferior fronto-occipital fasciculi (IFOF) are dissected using available primate databases. (**A**) Dissection of the IFOF in the Marmoset using *ex vivo* ultra-high resolution 150 μm dMRI (https://marmosetbrainmapping.org/data.html#exvivo).^72^ (**B**) Dissection of the IFOF in the squirrel monkey using *in vivo* ultra-high resolution 400 μm dMRI (http://saimiri.bcblab.com).^73^ (**C**) Dissection of the IFOF in the Macaque using *ex vivo* ultra-high resolution 600 μm dMRI (The University of Oxford WIN macaque post-mortem dataset, http://fcon_1000.projects.nitrc.org/indi/PRIME/oxford2.html).^74^ (**D**) A chimpanzee's IFOF dissected from published *ex vivo* diffusion data [500 μm dMRI; (https://edmond.mpg.de/dataset.xhtml?persistentId=doi:10.17617/3.O5XSI9)].^71^ (**E**) The human IFOF is reconstructed in a healthy subject using high-resolution *in vivo* 1.6 mm dMRI. OFC = orbitofrontal cortex; SFG = superior frontal gyrus; pMFG = posterior middle frontal gyrus; pTRI = pars triangularis; pORB = pars orbitalis; STG = superior temporal gyrus; MTG = middle temporal gyrus; VWFA = visual word form area; SOG = superior occipital gyrus; MOG = middle occipital gyrus; IOG = inferior occipital gyrus; SPL = superior parietal lobule.

Other techniques, such as simian blunt dissection or tractography, may offer insight on the subcortical trajectories of these pathways. Occipital connections to the frontal lobe passing within the extreme/external capsule have been described using combined blunt dissection in vervet monkeys and tractography in vervet monkeys and macaques connecting ventral prefrontal (47) and orbitofrontal (10, 11) cortices with V1 and V2 regions.^66^ Similarly, projections between the occipital lobe and the ventral frontal lobe have been identified with blunt dissection^67^ and tractography^68-70^ in macaques. It is important to note that, using tractography, Barrett and colleagues^47^ have showed a substantial expansion of IFOF's volume and projections in the frontal lobe along the primate lineage: while in the vervet monkey only orbitofrontal were dissected, these include also middle frontal and inferior frontal projections in humans. Similar results have been discussed by Roumazeilles and colleagues, who showed a progressive volume expansion of this tract between lemurs, vervet monkeys, macaques, gorilla and chimpanzee.^69,70^ Notably, the IFOF has been clearly delineated in a recent, high-resolution diffusion dataset acquired in chimpanzees.^71^ Therefore, a pattern of connections that may constitute the simian IFOF has been reproduced along primate phylogeny, which indicates that this pathway may not be exclusive to humans but may have evolved early along simian lineage—potentially before the prosimian-simian division as it is present in lemurs, which diverged from anthropoid monkeys ∼63 million years ago (Fig. 3).^69^

### Putative functional evidence for the IFOF in non-human primates

Being present along the primate phylogeny, the IFOF has likely anteceded the development of language,^75^ as opposed to other connections associated with language, such as the arcuate fasciculus.^28,75,76^ Regions connected by this network in the primate have been linked to non-spatial, feature-based selective attention in cluttered scenes.^77-80^ Early studies during attentional tasks showed that both FEF and V4 would contribute to tasks requiring selection of object qualities, such as shape or colour.^57^ Further, connections between V4 and the ventral prearcuate area have been shown to subserve non-spatial attention focused on objects’ characteristics,^77,78^ and connections between the antero-lateral field in macaque non-primary auditory cortex to the frontal pole (area 10), the rostral principal sulcus (area 46), the inferior convexity (areas 12 vl and 45) and the lateral orbital cortex (areas 11, 12o) have been linked to object-centred auditory processing.^81^ In a high-dimensional visual or acoustic environment, this may reflect a top-down signal that biases responses in favour of relevant features^77,82^ with the aim of resolving object competition for sensory processing.^81,83^ A similar form of executive control has been proposed for goal-directed motor behaviour,^58^ as regions at IFOF's cortical terminations are involved in processing of non-spatial information of object property or identity for selecting or controlling motor action,^58,84^ with object's features selection shaped by context or memory.^85,86^ Therefore, regions at putative cortical terminations of the simian IFOF may contribute to controlled selection of sensory features for both perception and action, potentially linking object awareness with motor cognition.

## Ontology of the IFOF in humans

The human IFOF can be identified in tractography as early as the 17th gestational week,^87^ and in blunt dissection after the 19th gestational week.^88^ In the fetal brain, the IFOF is strictly associated with the uncinate fasciculus, and these two pathways can be dissociated in blunt dissection only posterior to the cross-over between the limen insulae and the sagittal stratum. The uncinate/IFOF complex seems to mature before other cortico-cortical pathways, which start to be distinguished from the 30th gestational week, and especially the arcuate fasciculus/superior longitudinal fasciculus complex that can be identified only on a later stage from the 32nd to 35th week.^87,88^ Similarly, when considering values of fractional anisotropy as a proxy for tract maturation, the IFOF may be completely mature before the age of 7, which is in contrast with other fascicles that mature later, such as the arcuate fasciculus.^89^ It is important to note that not all components of the IFOF may mature at the same time: while more ventral projections starting from the inferior frontal gyrus are present at birth, others linking to them dorsolateral prefrontal seem to develop later during childhood, suggesting there may already be some differentiation within this fascicle during development.^90^

## Putative anatomy of the IFOF in humans

Historical blunt dissections of the IFOF did not disclose specific cortical terminations. At the beginning of the 2000s, the development of tractography provided a non-invasive method to track these *in vivo*. Capitalizing on previous work of Crosby and Nieuwenhuys,^91,92^ Catani and colleagues first discussed anterior terminations in orbitofrontal, inferior frontal and dorsolateral prefrontal cortices and posterior terminations within posterior temporal and inferior and medial occipital cortex.^40^ Since then, fronto-occipital connections linking orbitofrontal (area 10 and 11) and inferior frontal (area 45 and 47) with the occipital lobe (area 18 and 19) have been considered the core of IFOF's projections,^4,44^ which was clearly differentiated from another fronto-temporal connection, the uncinate fasciculus.^40,45,93^

The introduction of cortex-sparing blunt dissection at the beginning of 2010 revealed larger anterior and posterior projections.^45,46^ Martino and colleagues highlighted that from the level of the ventral external capsule, two components within this fascicle could be identified posteriorly: a more dorsal, projecting to the superior parietal (area 7), superior occipital (area 19) and middle occipital cortices (area 18), and a ventral portion, projecting instead basally to inferior occipital (area 19), inferior temporal and fusiform cortices (area 37).^45^ Sarubbo and colleagues suggested a similar division for anterior cortical terminations: a first, ventral component would start at the level of the ventral claustrum and terminate in the inferior frontal gyrus (area 47 and 45), and a second, dorsal component would occupy the ventral portion of the external capsule, further subdivided into posterior (middle and superior frontal gyri, area 8 and 9), middle (lateral orbitofrontal cortex) and anterior (frontal pole, area 10) components.^46^ This implies a dual-layer distribution within a continuous pathway: a ventral layer linking inferior frontal (area 45, 47) to inferior occipital, inferior temporal and fusiform cortices (area 19 and 37, ventral or superficial layer of the IFOF), and a dorsal layer linking orbitofrontal, superior and middle frontal projections (areas 8, 9, 10, 11) to superior, middle occipital and superior parietal projections (areas 7, 17, 18, dorsal or deep layer of the IFOF).^94^

An extended IFOF closer to cortex-sparing blunt dissection has been described since tractography algorithms capable of solving fibre crossing were developed in the late 2010.^95-97^ Together with a fronto-occipital core, additional frontal (middle frontal and superior frontal), parietal (superior parietal, angular) and medial occipital (cuneal, lingual) regions are now commonly highlighted.^95,98-100^ This also impacted IFOF layering. As an example, Wu and colleagues^98^ suggested five IFOF layers with anterior cortical termination in the frontal pole, orbitofrontal, superior, middle frontal and inferior frontal gyri and posterior in postcentral, angular, superior parietal and the whole occipital lobe. Conversely, Panesar and colleagues described three layers, in which orbitofrontal (ventromedial, area 10), inferior frontal (ventrolateral, area 47, 45, 44) and superior and middle frontal (dorsolateral, area 8, 9, 10) project to the occipitoparietal cortex (area 7, 17, 18, 19).^99^ These layers are, however, based on frontal cortical terminations: as posterior cortical terminations are largely shared among the proposed layers, these layer distinctions may be contentious. Importantly, the identification of posterior temporal cortical terminations in the middle temporal gyrus is recent,^42,94^ possibly because of these being intertwined with the arcuate fasciculus.^42^

The IFOF's asymmetry is another matter of debate. A rightward asymmetry for tract streamlines, but not tract volume, was initially suggested for the IFOF in a study of 40 right-handed healthy subjects.^101^ While a similar result was confirmed in a study of 60 healthy patients,^100^ this has not been confirmed in following studies.^98,99^ Similar analyses have been performed according to its layers: while Hau and colleagues suggested that the ventral layer may have rightward asymmetry and the dorsal, leftward asymmetry in volume,^100^ Vassal and colleagues suggested opposite asymmetry^102^ and asymmetrical patterns were not replicated by others.^98,99^

In summary, while early tractography studies described a fronto-occipital core for the IFOF linking orbitofrontal and inferior frontal to the lateral occipital cortex, results stemming from methodological advances in both blunt dissection (cortex-sparing Klingler dissection) and tractography (q-ball, spherical deconvolution tractography) suggest more extended projections, including superior and middle frontal, precuneal/superior parietal, posterior temporal, angular and medial occipital cortices. This extended IFOF is a continuous pathway composed of two layers: a ventral layer linking inferior frontal to the posterior temporal, basal temporal and inferior occipital gyri, and a dorsal layer connecting orbitofrontal and dorsolateral prefrontal with parietal and occipital regions. Nevertheless, as more complex layering patterns have also been described, subsegmental patterns and their asymmetry remain a matter of debate.

## Functional evidence for the IFOF in humans

Duffau and colleagues^6^ were the first to propose a role for the human IFOF in semantic language processing as direct white matter stimulation at trajectories of the IFOF induced semantic paraphasia during picture naming in awake surgery. While evidence for this is established,^9,34,103^ parallel lines of evidence have questioned a specific role in language, arguing for a wider contribution to visual processing.^40,104^ First, its pattern of cortical terminations largely exceeds language regions^42,45,46^ and evidence suggests it would also support functions, such as motor cognition,^16,18^ attention,^19,105^ theory of mind,^106,107^ face perception,^108^ emotion recognition^109^ and social behaviour.^110,111^ Second, stimulation, anatomical and lesional data indicate its role may be bilateral rather than left-lateralized—as it could be expected in case of a strict contribution to language.^48^ Finally, phylogenetic evidence suggesting that this tract may have evolved along with primates, combined with human developmental data indicating that this pathway may mature before speech acquisition, argues that its function likely extends beyond language.^87,88^

### A role for the human IFOF in top-down control of visual features

A reappraisal of IFOF's conflicting role in visual processing and language within cognitive control may provide a means to reconciliate existing literature. Classic semantic models for language rely on semantic representation: stored, multimodal conceptual knowledge about the world that can be generalized across items and context.^112,113^ Nevertheless, not all semantic disorders are disorders of representation. In everyday life, multiple representations of the same object coexist and compete, with one having to be retrieved and the others to be inhibited whenever behaviour is directed to a goal. For example, while a fork is generally used to hold (i.e. a piece of cheese), it could be used to scoop (i.e. jam), to cut (i.e. a piece of cake) but, depending on the context and goal, it could also be used for actions that are not classically associated with it but are allowed by some of its features (such as hammering a nail, opening a door, or even playing the drums if the fork's properties make it blunt enough). Further, actions to be performed with an object can change over time (i.e. when taking a train, train tickets sometimes need to be validated, and sometimes to be carried) meaning selection and inhibition of competing actions can be adaptive for the same item in a time-dependent manner, or may flexibly rely on conceptual (i.e. a crown or a sceptre may represent a king) or perceptual representations of an object (i.e. a chair is used for sitting, but in its absence one could sit on a box if this is rigid enough) according to context. This highlights that controlled selection of representations is critical for a task as the dominant representation is not always relevant to goal-directed behaviour, and poses a distinction within semantic cognition between stored semantic representations (semantic memory, which has been associated with a cortical hub at the level of the anterior ventral temporal lobe)^114^ and their regulated use according to context (semantic control, associated with other prefrontal, posterior temporal, infero-occipital and parietal regions).^115,116^

By connecting posterior temporal, parietal and occipital cortices with the prefrontal cortex, the IFOF is well placed to contribute to top-down control of semantic representation in the visual modality. Causal evidence from awake neurosurgery indicates that direct white matter stimulation in awake surgery induces semantic disorders in the visual domain, spanning comprehension and production, and occurring in both hemispheres.^43^ In the verbal modality, disorders of selection and/or inhibition occur at both word-level [lack of response (anomia),^117^ incorrect response (semantic paraphasia: i.e. ‘dog’ is uttered instead of target item ‘cat’),^6^ repetition of a previous item (verbal perseverations)]^20^ and sentence level [semantic jargonaphasia (i.e. ‘the scissor ran the ladder’ instead of ‘this is a dog’).^118^ In non-verbal modalities, its stimulation can affect visual semantics (PPTT), with patients being unable to correctly choose a picture to associate with a semantically related one.^7^

A role in top-down control of visual features may also account for deficits outside the semantic domain. Direct stimulation impacting face-based theory of mind [Reading the Mind behind the Eyes task (RMBE), where the patient cannot associate face features with the corresponding emotion],^119^ attention (with deviation in the line bisection task)^19^ and conscious awareness (with patients rating their responses with high confidence despite these being incorrect, or becoming unresponsive)^118,120^ may represent impaired selection or inhibition of perceptual features for action in line with a domain-general breakdown of goal-directed behaviour. Importantly, both semantic/conceptual and perceptual impairments of visual cognitive control are supported by lesion data: IFOF damage at its cortical terminations or along its subcortical trajectory impairs semantic control,^8,121^ visuospatial and object-centred attention,^19,105,122,123^ motor gestures and tool use,^16,18^ face perception, emotion recognition and theory of mind,^16,124-127^ with results often occurring in both hemispheres.^43,127^

In summary, while the IFOF has been associated with mapping acoustics to meaning,^128^ evidence from phylogeny, ontology, as well as behavioural literature outside language suggests it may play a larger involvement in context-dependent selection/inhibition of features for action. In line with current models of semantics (controlled semantic cognition)^114^ and causal evidence from direct stimulation,^6,48^ a role of the inferior fronto-occipital fasciculus in manipulation of visual features for goal-directed behaviour may reconcile discordant literature over the function of this fascicle in the field of language with attention, theory of mind, emotion perception and motor cognition, while also aligning with evidence from brain development and primate evolution.

### A graded dorso-ventral subdivision to guide perceptual versus conceptual cognitive control

Verbal semantics, non-verbal semantics, attentional, face-perception, emotion recognition, mentalizing and motor cognition deficits clustering along the trajectory of the IFOF may suggest a common mechanism for selection and manipulation of visual content according to context.^6^ However, accounts of semantic control indicate this to be graded, with a superior-inferior functional specialization^114^ in which damage to dorsal frontal regions predominantly impacts executive demands across multiple perceptual domains^129-131^ and ventral lesions within the inferior frontal gyrus impair manipulation of symbolic content^132^ or memory.^126^ Specifically, lesions of dorsal, middle frontal regions and their underlying white matter impacts multimodal cognitive control^129,130^ and overlap with sites associated with the multiple demand system, a fronto-parietal network active when perceptual control demands for action are high.^130,133,134^ Ventral lesions in the inferior frontal gyrus, in contrast, have been associated with conceptual deficits and are distinguished by defective control whenever the control demands are linked to abstract stimuli (i.e. words).^132^

We speculate the dorso-ventral distribution of IFOF layers may contribute to this perceptual versus conceptual gradient. On one hand, this aligns with functional MRI studies on cognitive control focusing on semantic versus perceptual hard tasks,^135^ showing a dorso-ventral distinction in semantic control versus multiple demand regions that may correspond with the IFOF's layers.^116,136^ Further, it aligns with stimulation data: in the left hemisphere verbal semantic deficits during picture naming occur at ventral regions while non-verbal semantic deficits tend to cluster in dorsal regions,^132,137^ in the right hemisphere visual semantic deficits induced by stimulation are usually ventral while mentalizing deficits are dorsal.^23,43^

It is important to stress that while the functional anatomy of the IFOF may be well suited to support cognitive control, this may not be the case for multimodal, stored, semantic representations. Recent models propose that conceptual knowledge—real world, multimodal context-independent representations that express conceptual similarity structure and can promote knowledge generalization across items and contexts—may be computed in a cortical hub in the ventral anterior temporal lobe.^114,138^ This is connected with modality-dependent, subordinate, cortical ‘spokes’, which can provide sensory-specific information regarding a concept.^114,138^ In this scenario, the pattern of connectivity and the functional literature reviewed for the IFOF may not support its direct involvement in semantic representations *per se*. First, anatomically, the IFOF does not reach the ventral anterior temporal lobe and therefore does not connect a most critical region for stored conceptual knowledge. Further, impairments induced by its direct stimulation are characterized by semantic paraphasia—potentially suggesting a deficit in feature selection—rather than agnosia, as would be expected for a loss of conceptual knowledge and demonstrated for stimulation of an adjacent fascicle linking the occipital lobe with the temporal pole, the inferior longitudinal fasciculus.^139^

In summary, we previously proposed that the IFOF may support top-down, stimulus-driven visual cognitive control. In the next paragraph, we will argue that within this overarching computation it may provide a graded, different contribution to cognitive control according to its layers, whether stimuli are conceptual (ventral layer, connecting inferior frontal to posterior temporal, temporobasal and inferior occipital cortices) or perceptual (dorsal layer, connecting orbitofrontal, middle frontal and potentially superior frontal regions with parietal and superior occipital cortices) in line with current models of semantic cognition, which envision a superior-inferior gradient within the semantic control network.^114^

### A ventral layer for conceptual semantic control

**Figure 4:**
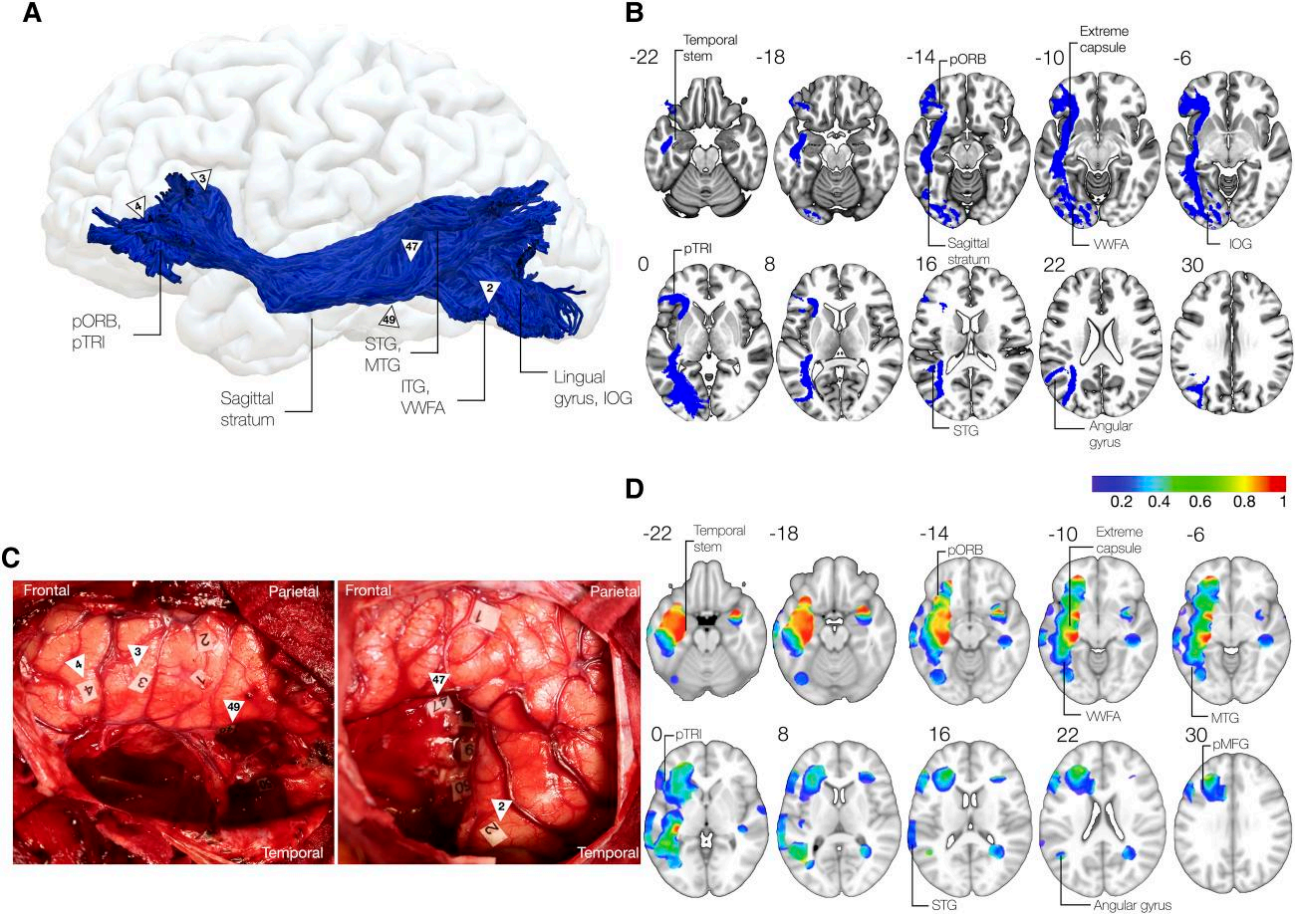
**Ventral component of the inferior fronto-occipital fasciculus**. (**A**) 3D reconstruction of ventral inferior fronto-occipital fasciculus (IFOF) projections from a high-resolution diffusion dataset (1.6 mm isotropic) in a healthy subject. (**B**) Sectional anatomy of ventral IFOF projections. (**C**) Intraoperative photos after resections showing cortical and subcortical stimulation at IFOF trajectory causing anomia and semantic paraphasia (arrowheads 2, 3, 4, 47, 49). (**D**) Distribution of non-verbal semantic disorders in a stimulation atlas of 256 awake patients showing bilateral distribution of induced errors.^43^ IOG = inferior occipital gyrus; ITG = inferior temporal gyrus; MTG = middle temporal gyrus; pORB = pars orbitalis; pTRI = pars triangularis; STG = superior temporal gyrus; VWFA = visual word form area.

The ventral subdivision of the inferior fronto-occipital fasciculus may be recruited whenever goal-directed behaviour relies on concepts (Fig. 4). Anatomically, the ventral layer connects the pars orbitalis, pars triangularis and potentially pars opercularis in the frontal lobe with the posterior temporal and inferior occipital cortices, including the posterior middle temporal gyrus, inferior temporal gyrus, inferior occipital gyrus and posterior fusiform gyrus, including the visual word form area. Critically, this layer may largely overlap with the extreme capsule fascicle/temporo-frontal-extreme capsule fascicle proposed in non-human primates^39^ and humans.^42^

As regions at its cortical terminations contribute to semantic control,^114^ this layer is well located to support top-down selection of semantic representations and semantic access.^115^ Semantic control deficits characterize semantic aphasia, a condition where poor comprehension for symbolic content^140^ is shared in verbal and non-verbal semantic tasks.^141^ Performance of patients with semantic aphasia is inconsistent along trials and improves whenever control demands in a task decrease (such as for low frequency items that have low contextual ambiguity or by using a cue).^115,141^ It is noteworthy that this system is important, especially in those cases when a stimulus-driven, automatic selection is not relevant for goal-driven behaviour—and therefore the task is ‘difficult’, meaning dominant but task-irrelevant stimuli have to be inhibited in favour of an unusual, task-relevant stimulus.^126,142^ As a result, deficits in semantic control not only impair distractors inhibition but are also characterized by disorders in selection or retrieval of more distant relationship between concepts or less frequent meaning dimensions.^113^ We speculate that damage to these ventral IFOF components, including posterior middle temporal gyrus and inferior temporal gyrus, would therefore impact selection of features in the verbal modality and contribute to classical comprehension syndromes in aphasia.^8^ These would include semantic aphasia,^115^ semantic jargonaphasia^118^ (in which selection of one or more inappropriate, dominant words, is an expression of disordered retrieval of symbolic features at word or sentence level)^43,115^ and pragmatics (in which meaning selected would not be context-appropriate).^143^ Finally, damage to the ventral IFOF may contribute to features of Wernicke's aphasia: while difficulties in phonological decoding may point to a damage to the arcuate fasciculus,^28^ impaired control of verbal representations after combined IFOF disconnection may produce both comprehension and production deficits.^144^

Damage to this ventral layer would also impact visual language. In reading, the IFOF has been proposed to underlie a ventral orthographical pathway for direct word access bypassing phonological conversion.^145^ Anatomically, damage to components from the visual word form area reaching the inferior frontal gyrus or even Exner area/area 55b in the posterior middle frontal gyrus may induce deregulated retrieval of written words causing substitution of words when reading or writing (semantic alexia and/or agraphia).^146,147^ In the same line, its neurodevelopmental damage may subtend deep dyslexia, a disorder where reading of words or sentences is marred by impaired word selection with semantic substitution of categorically similar words (i.e. semantic paralexia).^148^ Clinical evidence for a role of the IFOF in reading is accumulating as tractography analyses of IFOF maturation in children show this is associated with the acquisition of reading semantics^145,149^ and neurosurgical data that its damage may underlie lexico-semantic reading deficits.^147^ However, as data for subcortical mapping using word naming are limited when compared to picture naming, future work may expand this evidence.

In the right hemisphere, terminations of this component overlap both ‘core’ (occipital face area in the inferior occipital gyrus, fusiform face area in the posterior fusiform gyrus, and the superior temporal sulcus/middle temporal gyrus) and ‘extended’ (inferior frontal, orbitofrontal) regions of the so-called ‘face processing network’.^150^ Projections within this component have been linked with face processing^108,151^ especially in those cases where faces portray emotional valence.^152^ As a result, right IFOF damage can impact facial emotion recognition with specific impairments for sadness, anger and fear.^109^ Further, adaptive retrieval of conceptual knowledge is critical to emotional processing and semantic control deficits in the left hemisphere impair emotion perception (especially negative emotions),^152^ thus potentially suggesting a bilateral role for this layer in emotion processing. We speculate that this layer may support context-based retrieval of visual features with emotional valence (i.e. face expressions, but also actions), in line with evidence that patients with semantic control deficits and concomitant impairments of emotional perception benefit from word anchors and cue conditions that facilitate emotion perception by increasing access to relevant emotion concepts in ambiguous contexts.^152^ Critically, this is in agreement with evidence during awake surgery in which its stimulation impairs the attribution of affective mental states to images of eyes in the RMBE test,^119^ and lesion data showing that damage to this pathway impacts face-based theory of mind.^106,107,153^ Alternatively, as the RMBE carries high control demands, it is possible instead that these projections would underlie executive processes within the social/valence domain in line with recent models of social semantic control.^154^

Finally, by proposing a role in context-dependent, flexible manipulation of conceptual knowledge, it is important to clarify that the computation by this layer may be different from object recognition proposed for the visual ventral stream of vision. In Mishkin and colleagues,^155^ the ventral stream is devoted to recognition of high-resolution features of stimuli for object recognition—in line with retrieval of stored visual conceptual knowledge^114^—and is supported by connections between the occipital lobe and the ventral anterior temporal lobe. Even if a role for this IFOF component in the ventral stream may be considered, some evidence suggests the ventral IFOF may not support this. First, the reviewed evidence suggests it may not connect the ventral anterior temporal lobe. Second, it does not seem to be involved in recognition of context-independent, stored, conceptual representations (which has been linked to the inferior longitudinal fasciculus^139,156,157^), with reviewed literature suggesting, instead, it may support a supervisory executive role (semantic control) by adaptively selecting symbolic features in a context-dependent manner for goal-directed actions.

### A dorsal layer for perceptual executive control

**Figure 5:**
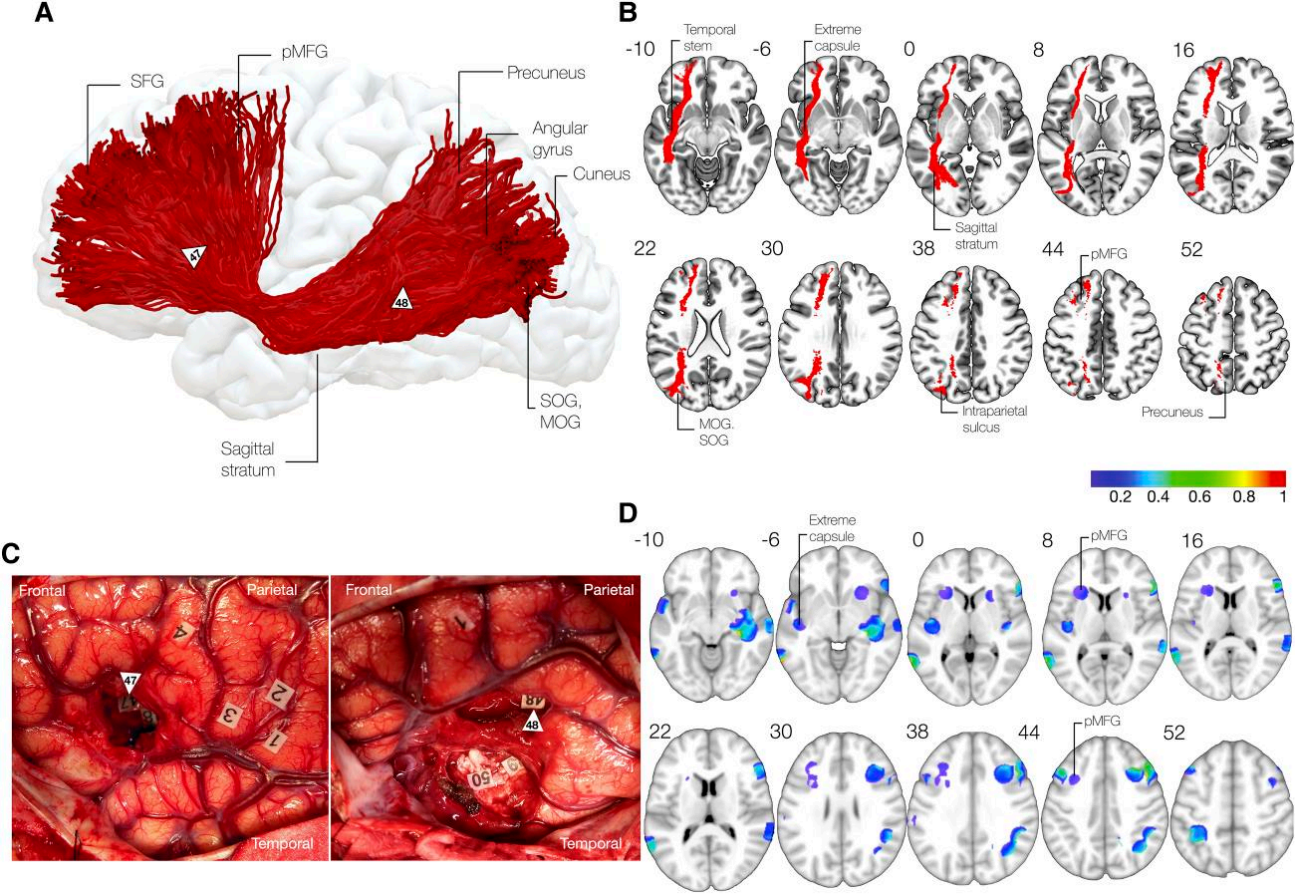
**Dorsal component of the inferior fronto-occipital fasciculus**. (**A**) 3D reconstruction of dorsal inferior fronto-occipital fasciculus (IFOF) projections from a high-resolution diffusion dataset (1.6 mm isotropic) in a healthy subject. (**B**) Sectional anatomy of dorsal IFOF projections. (**C**) Intraoperative photos after resections showing subcortical stimulation at IFOF trajectory causing disorders in the Pyramid and Palm tree task (arrowhead 47) or conscious awareness (arrowhead 48). (**D**) Distribution of non-verbal semantic disorders in a stimulation atlas of 256 awake patients showing bilateral distribution of induced errors.^43^ MOG = middle occipital gyrus; OFC = orbitofrontal cortex; pMFG = posterior middle frontal gyrus; SFG = superior frontal gyrus; SOG = superior occipital gyrus.

The dorsal subdivision of the inferior fronto-occipital fasciculus may be recruited, instead, when goal-directed behaviour relies on perception (Fig. 5). By connecting the middle frontal gyrus and potentially superior frontal regions^100,158^ with the parietal lobe—including the angular gyrus, superior parietal gyrus and the precuneus—and the occipital lobe, the dorsal layer of the IFOF joins areas within the multiple demand network.^134,159,160^ Regions at its cortical terminations are recruited, especially when a decision is perceptual or the strategy for task resolution relies on sensory features (‘non-semantic control’).^116,135^ This form of cognitive control is exemplified in tasks involving solving complex, meaningless pictures,^80^ which require selective attention of some object features when inhibiting others,^80,130^ or to perform combination of movements to achieve a goal according to object-features, as for object/tool manipulations^125,141^ or even mental rotation.^159^ In both hemispheres, direct stimulation of projections linking parietal to dorsolateral prefrontal can impact non-verbal semantics and is exemplified by impairments during the PPTT.^43^ While some overlapping of non-verbal semantic deficits with the ventral layer may suggest that contextual ambiguity may be resolved via semantic control,^43^ more dorsal components may leverage instead on perceptual features, as supported by tractography analysis suggesting that this layer may be critical to inhibition efficiency in difficult perceptually-guided decisions^136^ and lesion analysis that it may span visuospatial attention, alertness and inhibition within multiple demand network.^161^

In line with regions at IFOF's cortical terminations in non-human primates being linked with top-down control of visual perception,^77,78^ IFOF components have been suggested to underlie attention.^19,122,161,162^ In contrast to projections of the second branch of the superior longitudinal fasciculus supporting visuospatial attention,^163,164^ dorsal IFOF's projections to gaze-orienting fields in the posterior middle frontal gyrus (frontal eye field, FEF) and medial superior frontal gyrus [supplementary eye field (SEF)] from extrastriate cortices in the occipital lobe as well as the angular gyrus at the level of the posterior intraparietal sulcus and the precuneus in the medial superior parietal lobe may support attention to object features.^165,166^ In this vein, Urbanski and colleagues have shown that disconnection of the IFOF was associated with impaired object-based discrimination (bells cancellation task), but not spatial perception (line bisection task), therefore suggesting impaired object identification.^105^ A similar result linking the IFOF to chronic feature-based neglect was found using letter cancellation, bells cancellation and copying tasks in a larger cohort of 66 stroke patients.^122^ Therefore, it is possible that this layer may participate in executive control to direct object-centred attention,^167^ especially in those cases in which subordinate but goal-relevant objects or objects features have to be selected when competing with other predominant task-irrelevant visual representations.^129,161^ In line with this, evidence from univariate and multivariate voxel-lesion symptom mapping suggests that attentional and executive control deficits co-occur with semantic control deficits.^127^ Importantly, a failure in selecting task-appropriate or inhibiting predominant visual features according to context may underlie visual hallucinations, another phenomenon linked to the IFOF.^168^ However, as direct electrical stimulation of the right IFOF has been shown to induce left hemispatial neglect in the line bisection task,^19^ a broader role spanning both non-spatial and spatial attention may be considered for this layer.^122^

Disorders of feature selection can also affect motor cognition. By testing object use, Corbett *et al*.^125^ have shown impaired tool manipulations whenever this required selecting perceptual object features but disregarding predominant semantic representation (i.e. placing a needle with a shoe, which requires considering the large surface of the shoe to place and inhibit the predominant feature ‘walking’). Similar results were showed in Hoeren *et al*., who suggested that imitation of meaningless and meaningful gestures is impaired whenever the IFOF is disconnected.^16^ Considering this, we speculate that damage to these components linking the intraparietal sulcus and potentially the superior parietal cortex/precuneus to the dorsolateral prefrontal cortex may underlie ideational apraxia following deregulated control of transitive actions.^125,169^ Intriguingly, as both imitation and pantomime deficits were linked to lesion of projections of the IFOF,^16,18^ selection and retrieval of object-centred motor actions/prototypes^170^ may be graded, imitation relying on perceptual features and the dorsal IFOF, pantomime on conceptual/semantic representations and the ventral IFOF.

Finally, stimulation of the dorsal IFOF linking the precuneus with the prefrontal cortex may impact conscious visual awareness. Stimulation at IFOF trajectory overlapping the precuneus, running into the temporal lobe, reaching the dorsolateral prefrontal cortex impairs self-evaluative processes in both hemispheres, with patients not being able to rate their performance after a mistake or, in the left hemisphere, becoming unresponsive (‘disconnected’) for several seconds and unaware of this after regaining responsiveness.^120^ In line with theories of consciousness, such as the global neuronal workspace,^171^ it is possible that impaired conscious access may follow a disruption of top-down attentional amplification^172^ during stimulation of this layer. Importantly, as deficits in self-evaluation co-occurred at the same sites in the individual patient in which other disturbances linked to the IFOF occurred (i.e. troubles in the PPTT or RMBE tasks), computations between cognitive control and conscious visual awareness may be shared.^120^

### Alternative interpretations

In the model proposed, we leveraged on structural (cortex-sparing Klingler dissection and tractography) and functional evidence (direct white matter stimulation, lesion and neuroimaging studies) to suggest a continuous white matter pathway linking the frontal lobe with the posterior hemisphere layered in two components, a ventral, more superficial fronto-temporal component and a dorsal, deeper fronto-parieto-occipital component. Alternative interpretations warrant discussion. It has been proposed that all cortico-cortical fibres passing via the extreme capsule may constitute a continuous fibre system.^42^ This would encompass projections from the frontal lobe to the anterior temporal lobe via the uncinate fasciculus, posterior fronto-temporal connections via the extreme capsule fascicle (largely overlapping with the ventral IFOF) and fronto-parieto-occipital projections via the IFOF (similar to the dorsal IFOF).^42^ Even if some studies have divided these layers on the basis of different fibre trajectory,^45,46,100^ anatomically it remains possible that these three tracts form a continuous pathway.^87,88^

Functionally, however, the stimulation data gathered suggest these connections may differ. Direct white matter stimulation of the uncinate fasciculus does not cause impairments in semantic language processing,^173^ attention,^163^ face-based mentalizing^48^ or motor cognition, which have been associated with the IFOF.^34^ Duffau and colleagues^173^ investigated this systematically in 13 patients showing that stimulation of the IFOF caused semantic paraphasia, whereas stimulation or resection of the uncinate fasciculus did not cause semantic deficits. This has been confirmed in an atlas of cortical and subcortical stimulation,^43^ which combined 1162 cortical and 659 subcortical stimulation sites in 256 patients undergoing awake surgery and showed that semantic paraphasia and non-verbal comprehension disorders were linked to the IFOF but not to the uncinate fasciculus (Supplementary Fig. 1). Additionally, it is worth noting that beyond the results obtained from direct electrical stimulation, the existing literature using other methods does not suggest assimilable functions for these tracts: differently from the IFOF, the uncinate fasciculus has been associated with proper naming^174,175^ and has been proposed to mediate memory (i.e mnemonic associations) for valence-based decision-making.^176^ As a result, while projections through the extreme/external capsule may constitute a continuous fibre complex, current evidence indicates that cognitive processes supported by the IFOF may differ from those supported by the uncinate fasciculus.^34,43,173^ Nonetheless, future studies incorporating dedicated neuropsychological testing in executive control may refine this hypothesis, potentially demonstrating that the uncinate fasciculus contributes to the computations performed by this broader fibre system^177^ or may take over part of these to aid recovery after IFOF damage.^178^

Another aspect to be considered is the potential role in the two layers of the IFOF in visual processing within dorsal and ventral visual streams.^179^ A possible interpretation is that, by connecting early dorsal extrastriate visual cortex and early ventral extrastriate cortex they may be involved, on one hand, in perception of object movement in space within the dorsal stream and, on the other hand, in fine-grained recognition of object features with the ventral stream. Other white matter pathways sharing terminations in these dorsal and ventral extrastriate regions, however, may also be well placed to perform these computations based on anatomy and direct stimulation in humans. The superior longitudinal fasciculus may contribute to the dorsal visual stream as this has been linked to spatial attention^180,181^ as its stimulation causes spatial neglect during line bisection tasks.^163^ Conversely, the inferior longitudinal fasciculus may play a role in the ventral visual stream as it links the occipital lobe to the ventral anterior temporal lobe, as predicted from findings in non-human primates,^182^ with its stimulation causing visual agnosia^139^ and visual paraphasias^156^ in line with a deficit in object recognition.

## Surgical implications

The development of white matter stimulation during awake surgery has radically changed functional outcomes in resective neurosurgery, allowing for an incidence of permanent motor and language deficits in oncological resection of 3%,^183^ with a return to work in up to 97% in patients operated for low grade glioma.^184^ In the left hemisphere, the IFOF has long been considered a white matter tract for preservation as it has been shown to underlie verbal semantics.^6,34^ Our review, however, argues that it may support this via a more general top-down, context-based control of conceptual and perceptual content. This is not novel, as similar arguments aiming at including domain-general cognitive control in the neurobiology of language,^185^ as well as language models integrating executive control, have been proposed presently.^114,186^

**Figure 6:**
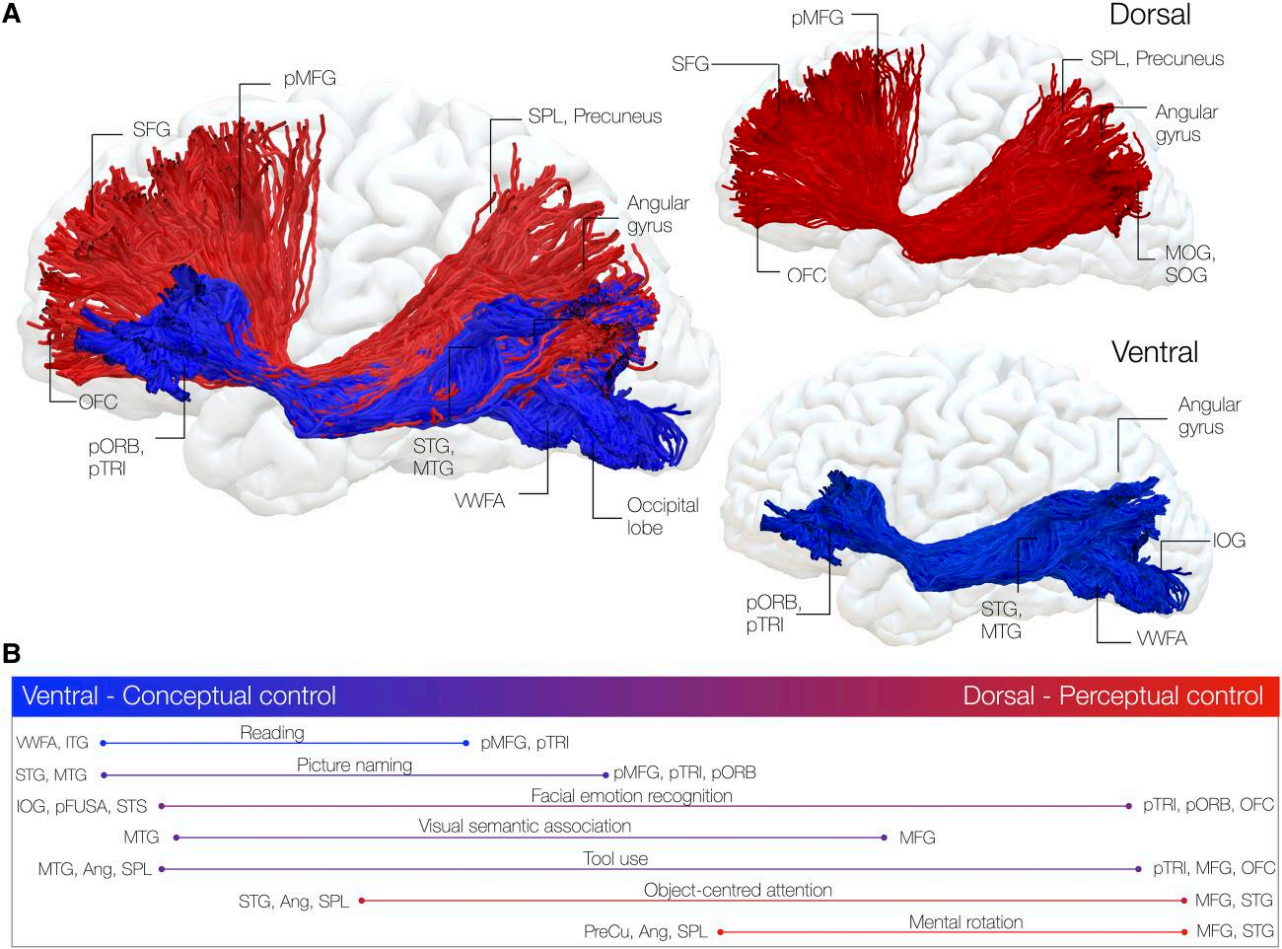
**Functional gradients for controlled behaviour within layers of the inferior fronto-occipital fasciculus**. A dorso-ventral subdivision of inferior fronto-occipital fasciculus (IFOF) is shown. *Left*: The two segments are shown together as a continuous pathway. *Right*: The two segments are shown separately: the dorsal IFOF may link superior, middle frontal and orbitofrontal to parieto-occipital regions to support perceptual control, the ventral IFOF may link temporal to inferior frontal regions to support conceptual control. *Bottom*: Putative cortico-cortical interactions within this graded model. Red: dorsal component of the IFOF; blue: ventral component of the IFOF. Ang = angular gyrus; IOG = inferior occipital gyrus; MOG: middle occipital gyrus; MTG = middle temporal gyrus; OFC = orbitofrontal cortex; pFUSA = posterior fusiform gyrus; pMFG = posterior middle frontal gyrus; pORB = pars orbitalis PreCu = precuneus; pTRI = pars triangularis; SFG = superior frontal gyrus; SOG = superior occipital gyrus; SPL = superior parietal lobule; STG = superior temporal gyrus; STS = superior temporal sulcus; VWFA = visual word form area.

As discussed, the IFOF may contribute to controlled retrieval of semantic representations rather than semantic memory. Accordingly, a complete transection of this fascicle (i.e. at the level of the sagittal stratum or temporal stem) may be associated with deficits in multimodal semantic control, with verbal semantic, non-verbal semantic, motor cognition, emotion recognition and attentional systems being affected.^115,187^ Selective disconnection of its layers, however, may account for more graded impairments, which are commonly reported in clinical practice. One such case is the co-occurrence of apraxia with aphasia after left temporoparietal damage.^188^ It is possible that disconnection of dorsal and ventral components of the left IFOF may contribute separately to this phenomenon by deregulating retrieval of conceptual (aphasia) and perceptual (apraxia) knowledge, although this will need to be tested (Fig. 6). Regardless, this review emphasizes that perceptual and conceptual cognitive control may dissociate within IFOF segments and therefore intraoperative testing of the IFOF should be flexibly adapted. Under this premise, newer, tailored intraoperative tests may reveal facets of cognitive control associated with the IFOF that would allow better prediction of, and therefore prevent, the risk to induce specific higher-order cognitive deficit after surgery.

If structural data on IFOF's lateralization are conflicting, stimulation data suggest this may have a bilateral distribution.^48^ This is in sharp contrast with other fascicles, such as the arcuate fasciculus, which appears strongly lateralized.^101^ While a more bilateral distribution for semantic processes was already postulated in Hickok and Poeppel^15^ for ventral stream functions, the gathered evidence suggests that these may also reflect bilateral distribution of cognitive control processes, in line with domain-general, language-aspecific computations recently proposed.^189^ Critically, these results may align with evidence from plastic reorganization. In an atlas of brain plasticity, Herbet and colleagues^190^ showed that the IFOF may have high plastic potential if one layer is preserved; it is possible that this form of recovery may be allowed by a more bilateral functional distribution of this tract, as suggested by stimulation and potentially by the absence of a clear lateralization pattern.

Above all aspects, data stressing a role for the right IFOF in cognitive control challenge the traditional surgical view of a non-dominant hemisphere. In current surgical practice, awake mapping for intraoperative functional preservation is advocated for the left hemisphere on the basis of language preservation.^191^ However, the IFOF's role in attention, theory of mind, face perception and emotion regulation in the right hemisphere suggests other aspects of social behaviour may need mapping to ensure their safeguarding. It is acknowledged that patients may change behaviour after surgery, but this is often difficult to quantify. Deficits in social behaviour are difficult to diagnose, with a discrepancy between a late clinical diagnosis and early family's report of inappropriate behaviour.^192^ As a system for controlled social semantic control in the right hemisphere mirroring computations of the left hemisphere has been proposed^193^ and abnormalities of IFOF's microstructure are robustly associated with neuropsychiatric disorders of social cognition, such as autism^194^ or schizophrenia,^195-199^ it is mandatory to re-evaluate in surgical practice the effect that its disconnection may have on patients’ behaviour. Indeed, the inability to inhibit predominant norms may determine cognitive inflexibility/perseveration (inability to appropriately update behaviour to context) and, on the other hand, impaired ability to perceive social cues and emotions of others in ambiguous context may underlie anti-social behaviour—with recent lesion network mappings linking structural damage to regions in the right hemisphere at cortical termination of the IFOF and deranged social behaviour (including criminality).^200,201^ Accordingly, we believe these results add evidence to the claim that the so-called non-dominant right hemisphere is eloquent,^202^ and awake surgery in right hemisphere procedures should be considered to preserve it and avoid impairing control on multiple facets of behaviour.^203^

## Limitations

In this review, we discussed evidence suggesting that the IFOF may contribute to aspects of visual executive control in both the left and right hemisphere. However, several caveats should be noted. First, alongside anatomical evidence for the IFOF in non-human primates, we have discussed putative functional computations. If it has been shown that cortices connected by the simian IFOF contribute to the same function (i.e. non-spatial attention),^77,78,84^ the attribution of these computations specifically to the IFOF remains inferential and will need validation. Second, models for semantic cognition are multimodal and control of semantic representation is suggested to span across sensory modalities. While a role for the IFOF in acoustic stimuli has also been proposed,^8^ tasks impaired in most of the studies reviewed relied on vision. As a result, we discussed the role within cognitive control supported by the IFOF as specific to vision, and further research is necessary to determine whether this fascicle is, instead, amodal or multimodal. Third, while direct stimulation suggests that this pathway may contribute to executive control, this does not encompass the entire cognitive control network. This wider network includes other regions linked by a larger system of cortico-cortical, cortico-striatal, cortico-thalamic and brainstem connections.^204,205^ As an example, the anterior insula is critical to cognitive control as it arbitrates (‘gatekeeper’)^206^ salience between competing external sensory information and internal states.^207^ Thus, the anterior insula may gate activity in regions connected by the IFOF: it may support their action (in case of goal-directed behaviours that require manipulation of external stimuli), but also inhibit them (e.g. in conditions in which internal states, such as interoception or memory need to be prioritized). Similarly, the matrix thalamus may direct arousal to support attention and working memory allocation,^204^ while hyperdirect pathways to the subthalamic nucleus may regulate fast inhibitory control to pause or stop action.^208-210^ These aspects of cognitive control likely operate independently of the computations performed by the IFOF. Fourth, while we propose that components of the IFOF serve top-down control of visual features, we cannot exclude that its cortical terminations at primary visual regions may also support bottom-up processes, as proposed for visual emotion or object recognition.^150^ Fifth, components of the IFOF at the level of the middle temporal gyrus are intertwined with projections of the arcuate fasciculus, which may put into question whether semantic deficits at this level are caused by stimulation of one or the other fascicle. If, when considering stimulation in a single site, it is not possible to discern whether deficits evoked are caused by one or the other tract, differences between these two tracts become evident when stimulating distant tract location in the same patient or when analysing stimulation at a group level, as stimulation of the arcuate fasciculus reliably causes phonological paraphasia or phonological distortions,^28,211^ while stimulation of the IFOF instead causes predominantly semantic paraphasia.^6^ Finally, this review relies heavily on stimulation data from awake surgery, as these can provide causal insights. Stimulation at cortical and subcortical level causing the same neuropsychological deficit, and stimulation atlases showing deficits clustering along the same pathway, point to a continuum from cortex to cortex in the same white matter pathway. Nevertheless, electrophysiological techniques using subcortical stimulation [ideally with stereoelectroencephalography (SEEG)] and recording at cortical terminations (subcortico-cortical/axono-cortical evoked potentials)^212,213^ as well as a collision technique test^214^ between the two sites are needed to provide definitive proof of an anatomical connection.

## Conclusions

Since its description with blunt dissection, the anatomy and function of the IFOF have been a matter of debate. By capitalizing on neuro-evolutionary literature, this review posits that this white matter pathway likely appeared early in primate evolution to facilitate the selection of visual features for action. In humans, we propose that the IFOF may underlie controlled manipulation of visual features spanning language, theory of mind, reading, attention, emotional processing and motor cognition. Furthermore, in line with its anatomical distinction in a dorsal and ventral layer, we propose that the dorsal IFOF is recruited for context-dependent cognitive control grounded in concrete, perceptual features, whereas the ventral IFOF is engaged when such control is reliant on abstract, symbolic features. This conceptual framework integrates findings across various cognitive domains and suggests a nuanced role for the IFOF in human cognition. Moreover, it advocates for a paradigm shift in current surgical practices, challenging the conventional view of a dominant hemisphere and predicating that brain mapping is required to preserve cognition in both sides.^202^

## Supplementary Material

awaf055_Supplementary_Data

## Data Availability

The data on the healthy subject can be obtained after reasonable request to the first author. *Ex vivo* ultra-high resolution 150 μm dMRI can be found at https://marmosetbrainmapping.org/data.html#exvivo.^72^*In vivo* ultra-high resolution 400 μm dMRI data for squirrel monkeys can be found at http://saimiri.bcblab.com.^73^*Ex vivo* ultra-high resolution 600 μm dMRI data from the University of Oxford WIN macaque post mortem dataset can be found at http://fcon_1000.projects.nitrc.org/indi/PRIME/oxford2.html.^74^*Ex vivo* ultra-high resolution 500 μm dMRI chimpanzee data can be found at https://edmond.mpg.de/dataset.xhtml?persistentId=doi:10.17617/3.O5XSI9.^71^
