# Supplementary material for: The inferior fronto-occipital fasciculus: bridging phylogeny, ontogeny and functional anatomy
Source: Brain. 2025 Feb 11;148(5):1507–25. doi: 10.1093/brain/awaf055 (PMC12074009; doi:10.1093/brain/awaf055)
Supplement: awaf055_Supplementary_Data [file awaf055_supplementary_data.pdf]

Supplementary material

Data Sources

Research was conducted from 2022 to 2024, with updated literature search until the 2nd of April 2024. The search was conducted in PubMed using the search term "inferior fronto occipital fasciculus" OR "inferior fronto occipital fascicle" OR "IFOF" OR "arcuate fascicle" OR "extreme capsule fascicle", which returned a total of 1041 articles.

Data screening and eligibility

Suppl. Fig. 1 summarises the following workflow. During the screening, review articles (48), non-brain studies (e.g. cranial nerves, spine, muscle, mechanics) (10), studies in which damage or cognitive performance reflected global/diffuse damage to white matter (52) and studies not available from PubMed (3) were excluded. 928 studies were included.

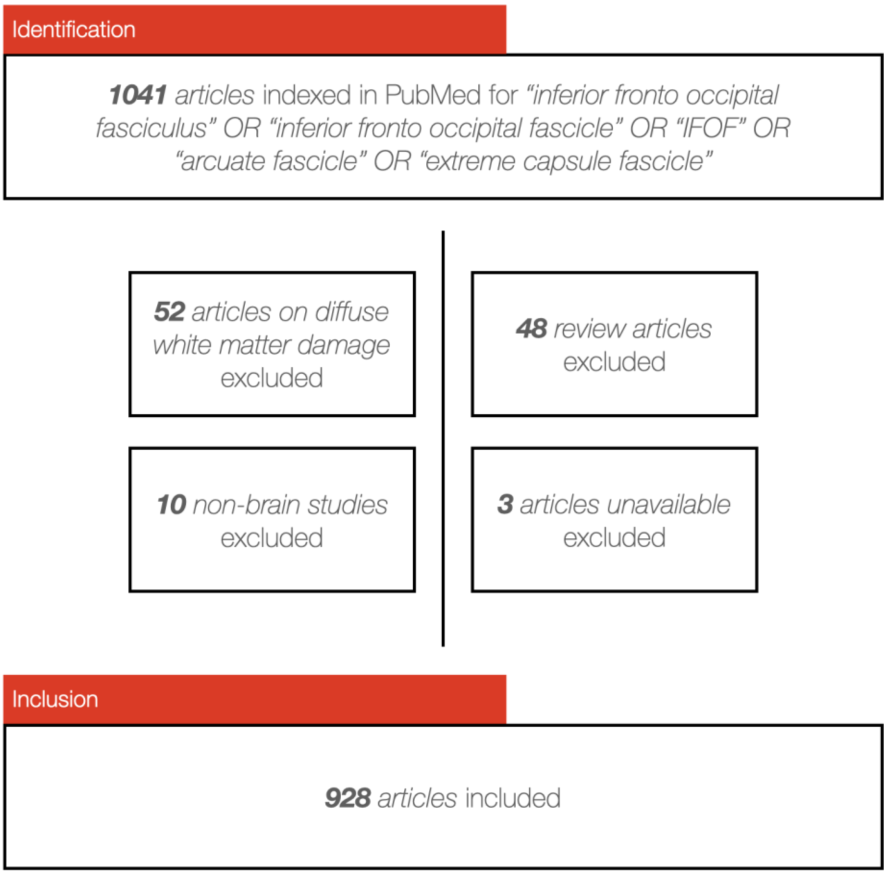

Supplementary Fig. 1 Flow-chart for inclusion

Data synthesis and analyses

Selected studies are reported in *Supplementary Table 1*.

Supplementary Table 1 Studies included for of the functional anatomy of the inferior fronto-occipital fasciculus along the primate lineage

No	Title	Authors	Journal/Book	Publication Year
1	Association fiber pathways to the frontal cortex from the superior temporal region in the rhesus monkey	Petrides M, Pandya DN.	J Comp Neurol	1988
2	Topography of the uncinate fascicle and adjacent temporal fiber tracts	Ebeling U, von Cramon D.	Acta Neurochir (Wien)	1992
3	Magnetic resonance imaging of cerebral associative white matter bundles employing fast-scan techniques	Cellerini M, Konze A, Caracchini G, Santoni M, Dal Pozzo G.	Acta Anat (Basel)	1997
4	Virtual in vivo interactive dissection of white matter fasciculi in the human brain	Catani M, Howard RJ, Pajevic S, Jones DK.	Neuroimage	2002
5	New insights into the anatomo-functional connectivity of the semantic system: a study using cortico-subcortical electrostimulations	Duffau H, Gatignol P, Mandonnet E, Peruzzi P, Tzourio-Mazoyer N, Capelle L.	Brain	2005
6	Efferent association pathways originating in the caudal prefrontal cortex in the macaque monkey	Petrides M, Pandya DN.	J Comp Neurol	2006
7	Age effects on diffusion tensor magnetic resonance imaging tractography measures of frontal cortex connections in schizophrenia	Jones DK, Catani M, Pierpaoli C, Reeves SJ, Shergill SS, O'Sullivan M, Golesworthy P, McGuire P, Horsfield MA, Simmons A, Williams SC, Howard RJ.	Hum Brain Mapp	2006
8	Diffusion tensor anisotropy in adolescents and adults	Schneiderman JS, Buchsbaum MS, Haznedar MM, Hazlett EA, Brickman AM, Shihabuddin L, Brand JG, Torosjan Y, Newmark RE, Tang C, Aronowitz J, Paul-Odouard R, Byne W, Hof PR.	Neuropsychobiology	2007
9	Association fibre pathways of the brain: parallel observations from diffusion	Schmahmann JD, Pandya DN, Wang R, Dai G, D'Arceuil HE, de Crespigny AJ, Wedeen VJ.	Brain	2007

	spectrum imaging and autoradiography			
**10**	Quantitative diffusion tensor tractography of association and projection fibers in normally developing children and adolescents	Eluvathingal TJ, Hasan KM, Kramer L, Fletcher JM, Ewing-Cobbs L.	Cereb Cortex	2007
**11**	Internal capsule, corpus callosum and long associative fibers in good and poor outcome schizophrenia: a diffusion tensor imaging survey	Mitelman SA, Torosjan Y, Newmark RE, Schneiderman JS, Chu KW, Brickman AM, Haznedar MM, Hazlett EA, Tang CY, Shihabuddin L, Buchsbaum MS.	Schizophr Res	2007
**12**	Diffusion tensor MR imaging reveals persistent white matter alteration after traumatic brain injury experienced during early childhood	Yuan W, Holland SK, Schmithorst VJ, Walz NC, Cecil KM, Jones BV, Karunanayaka P, Michaud L, Wade SL.	AJNR Am J Neuroradiol	2007
**13**	Thalamo-cortical connectivity in children born preterm mapped using probabilistic magnetic resonance tractography	Counsell SJ, Dyet LE, Larkman DJ, Nunes RG, Boardman JP, Allsop JM, Fitzpatrick J, Srinivasan L, Cowan FM, Hajnal JV, Rutherford MA, Edwards AD.	Neuroimage	2007
**14**	White matter integrity and cognition in chronic traumatic brain injury: a diffusion tensor imaging study	Kraus MF, Susmaras T, Caughlin BP, Walker CJ, Sweeney JA, Little DM.	Brain	2007
**15**	Brain networks of spatial awareness: evidence from diffusion tensor imaging tractography	Urbanski M, Thiebaut de Schotten M, Rodrigo S, Catani M, Oppenheim C, Touzé E, Chokron S, Méder JF, Lévy R, Dubois B, Bartolomeo P.	J Neurol Neurosurg Psychiatry	2008
**16**	Bipolar disorder and neurophysiologic mechanisms	McCrea SM.	Neuropsychiatr Dis Treat	2008
**17**	Clinical and neuropsychological correlates of white matter abnormalities in recent onset schizophrenia	Szeszko PR, Robinson DG, Ashtari M, Vogel J, Betensky J, Sevy S, Ardekani BA, Lencz T, Malhotra AK, McCormack J, Miller R, Lim KO, Gunduz-Bruce H, Kane JM, Bilder RM.	Neuropsychopharmacology	2008
**18**	White matter volume changes in people who develop psychosis	Walterfang M, McGuire PK, Yung AR, Phillips LJ, Velakoulis D, Wood SJ, Suckling J, Bullmore ET, Brewer W, Soulsby B, Desmond P, McGorry PD, Pantelis C.	Br J Psychiatry	2008
**19**	Diffusion tensor imaging correlates of memory and language impairments in temporal lobe epilepsy	McDonald CR, Ahmadi ME, Hagler DJ, Tecoma ES, Iragui VJ, Gharapetian L, Dale AM, Halgren E.	Neurology	2008
**20**	Functional relevant loss of long association fibre tracts integrity in early Alzheimer's disease	Fellgiebel A, Schermuly I, Gerhard A, Keller I, Albrecht J, Weibrich C, Müller MJ, Stoeter P.	Neuropsychologia	2008
**21**	Reduction in white matter connectivity, revealed by diffusion	Thomas C, Moya L, Avidan G, Humphreys K, Jung KJ, Peterson MA, Behrmann M.	J Cogn Neurosci	2008

	tensor imaging, may account for age-related changes in face perception			
**22**	Brain diffusion abnormalities in children with fetal alcohol spectrum disorder	Lebel C, Rasmussen C, Wyper K, Walker L, Andrew G, Yager J, Beaulieu C.	Alcohol Clin Exp Res	2008
**23**	A diffusion tensor imaging study of structural dysconnectivity in never-medicated, first-episode schizophrenia	Cheung V, Cheung C, McAlonan GM, Deng Y, Wong JG, Yip L, Tai KS, Khong PL, Sham P, Chua SE.	Psychol Med	2008
**24**	Elevating tensor rank increases anisotropy in brain areas associated with intra-voxel orientational heterogeneity (IVOH): a generalised DTI (GDTI) study	Minati L, Banasik T, Brzezinski J, Mandelli ML, Bizzi A, Bruzzone MG, Konopka M, Jasinski A.	NMR Biomed	2008
**25**	Regional white matter hyperintensity burden in automated segmentation distinguishes late-life depressed subjects from comparison subjects matched for vascular risk factors	Sheline YI, Price JL, Vaishnavi SN, Mintun MA, Barch DM, Epstein AA, Wilkins CH, Snyder AZ, Couture L, Schechtman K, McKinstry RC.	Am J Psychiatry	2008
**26**	Ventral and dorsal pathways for language	Saur D, Kreher BW, Schnell S, Kümmerer D, Kellmeyer P, Vry MS, Umarova R, Musso M, Glauche V, Abel S, Huber W, Rijntjes M, Hennig J, Weiller C.	Proc Natl Acad Sci U S A	2008
**27**	The extreme capsule in humans and rethinking of the language circuitry	Makris N, Pandya DN.	Brain Struct Funct	2009
**28**	Damage to association fiber tracts impairs recognition of the facial expression of emotion	Philippi CL, Mehta S, Grabowski T, Adolphs R, Rudrauf D.	J Neurosci	2009
**29**	White matter tracts associated with set-shifting in healthy aging	Perry ME, McDonald CR, Hagler DJ Jr, Gharapetian L, Kuperman JM, Koyama AK, Dale AM, McEvoy LK.	Neuropsychologia	2009
**30**	Localization of brain white matter hyperintensities and urinary incontinence in community-dwelling older adults	Kuchel GA, Moscufo N, Guttmann CR, Zeevi N, Wakefield D, Schmidt J, Dubeau CE, Wolfson L.	J Gerontol A Biol Sci Med Sci	2009
**31**	Neuroanatomical correlates of developmental dyscalculia: combined evidence from morphometry and tractography	Rykhlevskaia E, Uddin LQ, Kondos L, Menon V.	Front Hum Neurosci	2009
**32**	Damage to the right superior longitudinal fasciculus in the inferior parietal lobe	Shinoura N, Suzuki Y, Yamada R, Tabei Y, Saito K, Yagi K.	Neuropsychologia	2009

	plays a role in spatial neglect			
**33**	Abnormal integrity of association fiber tracts in amnestic mild cognitive impairment	Bai F, Zhang Z, Watson DR, Yu H, Shi Y, Yuan Y, Qian Y, Jia J.	J Neurol Sci	2009
**34**	White matter integrity predicts delay discounting behavior in 9- to 23-year-olds: a diffusion tensor imaging study	Olson EA, Collins PF, Hooper CJ, Muetzel R, Lim KO, Luciana M.	J Cogn Neurosci	2009
**35**	Widespread affections of large fiber tracts in postoperative temporal lobe epilepsy	Schoene-Bake JC, Faber J, Trautner P, Kaaden S, Tittgemeyer M, Elger CE, Weber B.	Neuroimage	2009
**36**	White matter integrity in adolescents with histories of marijuana use and binge drinking	Jacobus J, McQueeny T, Bava S, Schweinsburg BC, Frank LR, Yang TT, Tapert SF.	Neurotoxicol Teratol	2009
**37**	White matter abnormalities and executive function in children with very low birth weight	Skranes J, Lohaugen GC, Martinussen M, Indredavik MS, Dale AM, Haraldseth O, Vangberg TR, Brubakk AM.	Neuroreport	2009
**38**	Normal regional fractional anisotropy and apparent diffusion coefficient of the brain measured on a 3 T MR scanner	Lee CE, Danielian LE, Thomasson D, Baker EH.	Neuroradiology	2009
**39**	Altered white matter microstructure in adolescent substance users	Bava S, Frank LR, McQueeny T, Schweinsburg BC, Schweinsburg AD, Tapert SF.	Psychiatry Res	2009
**40**	Simple developmental dyslexia in children: alterations in diffusion-tensor metrics of white matter tracts at 3 T	Rollins NK, Vachha B, Srinivasan P, Chia J, Pickering J, Hughes CW, Gimi B.	Radiology	2009
**41**	Joint source based morphometry identifies linked gray and white matter group differences	Xu L, Pearlson G, Calhoun VD.	Neuroimage	2009
**42**	Anatomic dissection of the inferior fronto-occipital fasciculus revisited in the lights of brain stimulation data	Martino J, Brogna C, Robles SG, Vergani F, Duffau H.	Cortex	2010
**43**	New insights into the anatomic dissection of the temporal stem with special emphasis on the inferior fronto-occipital fasciculus: implications in surgical approach to left mesiotemporal and temporoinsular structures	Martino J, Vergani F, Robles SG, Duffau H.	Neurosurgery	2010
**44**	Disrupted white matter integrity of corticopontine-	Koch K, Wagner G, Dahnke R, Schachtzabel C, Schultz C, Roebel M, Güllmar D, Reichenbach JR, Sauer H, Schlösser RG.	Eur Arch Psychiatry Clin Neurosci	2010

	cerebellar circuitry in schizophrenia			
**45**	Assessment of white matter tract damage in mild cognitive impairment and Alzheimer's disease	Pievani M, Agosta F, Pagani E, Canu E, Sala S, Absinta M, Geroldi C, Ganzola R, Frisoni GB, Filippi M.	Hum Brain Mapp	2010
**46**	White matter integrity and cognitive impairment in first-episode psychosis	Pérez-Iglesias R, Tordesillas-Gutiérrez D, McGuire PK, Barker GJ, Roiz-Santiañez R, Mata I, de Lucas EM, Rodríguez-Sánchez JM, Ayesa-Arriola R, Vazquez-Barquero JL, Crespo-Facorro B.	Am J Psychiatry	2010
**47**	Changes in fiber tract integrity and visual fields after anterior temporal lobectomy	McDonald CR, Hagler DJ Jr, Girard HM, Pung C, Ahmadi ME, Holland D, Patel RH, Barba D, Tecoma ES, Iragui VJ, Halgren E, Dale AM.	Neurology	2010
**48**	The temporal stem in traumatic brain injury: preliminary findings	Bigler ED, McCauley SR, Wu TC, Yallampalli R, Shah S, MacLeod M, Chu Z, Hunter JV, Clifton GL, Levin HS, Wilde EA.	Brain Imaging Behav	2010
**49**	Disorganization of anatomical connectivity in obsessive compulsive disorder: a multi-parameter diffusion tensor imaging study in a subpopulation of patients	Garibotto V, Scifo P, Gorini A, Alonso CR, Brambati S, Bellodi L, Perani D.	Neurobiol Dis	2010
**50**	Deficits in the left inferior longitudinal fasciculus results in impairments in object naming	Shinoura N, Suzuki Y, Tsukada M, Yoshida M, Yamada R, Tabei Y, Saito K, Koizumi T, Yagi K.	Neurocase	2010
**51**	Assessment of white matter tract damage in patients with amyotrophic lateral sclerosis: a diffusion tensor MR imaging tractography study	Agosta F, Pagani E, Petrolini M, Caputo D, Perini M, Prelle A, Salvi F, Filippi M.	AJNR Am J Neuroradiol	2010
**52**	Regional distribution and clinical correlates of white matter structural damage in Huntington disease: a tract-based spatial statistics study	Della Nave R, Ginestroni A, Tessa C, Giannelli M, Piacentini S, Filippi M, Mascalchi M.	AJNR Am J Neuroradiol	2010
**53**	Quantification of the spatiotemporal microstructural organization of the human brain association, projection and commissural pathways across the lifespan using diffusion tensor tractography	Hasan KM, Kamali A, Abid H, Kramer LA, Fletcher JM, Ewing-Cobbs L.	Brain Struct Funct	2010
**54**	Microstructural brain differences predict functional hemodynamic responses in a reward processing task	Camara E, Rodriguez-Fornells A, Münte TF.	J Neurosci	2010
**55**	Longitudinal changes in fiber tract integrity in healthy aging and mild	Teipel SJ, Meindl T, Wagner M, Stieltjes B, Reuter S, Hauenstein KH, Filippi M, Ernemann U, Reiser MF, Hampel H.	J Alzheimers Dis	2010

	cognitive impairment: a DTI follow-up study			
**56**	Diffusion tensor imaging of Aicardi syndrome	Wahl M, Strominger ZA, Wakahiro M, Jeremy RJ, Mukherjee P, Sherr EH.	Pediatr Neurol	2010
**57**	Structural and functional brain correlates of subclinical psychotic symptoms in 11-13 year old schoolchildren	Jacobson S, Kelleher I, Harley M, Murtagh A, Clarke M, Blanchard M, Connolly C, O'Hanlon E, Garavan H, Cannon M.	Neuroimage	2010
**58**	Alterations in frontal lobe tracts and corpus callosum in young children with autism spectrum disorder	Kumar A, Sundaram SK, Sivaswamy L, Behen ME, Makki MI, Ager J, Janisse J, Chugani HT, Chugani DC.	Cereb Cortex	2010
**59**	Smoking and schizophrenia independently and additively reduce white matter integrity between striatum and frontal cortex	Zhang X, Stein EA, Hong LE.	Biol Psychiatry	2010
**60**	Tract-based spatial statistics on diffusion tensor imaging in systemic lupus erythematosus reveals localized involvement of white matter tracts	Emmer BJ, Veer IM, Steup-Beekman GM, Huizinga TW, van der Grond J, van Buchem MA.	Arthritis Rheum	2010
**61**	Structural and functional magnetic resonance imaging correlates of motor network dysfunction in primary progressive multiple sclerosis	Ceccarelli A, Rocca MA, Valsasina P, Rodegher M, Falini A, Comi G, Filippi M.	Eur J Neurosci	2010
**62**	Abnormal integrity of long association fiber tracts is associated with cognitive deficits in patients with remitted geriatric depression: a cross-sectional, case-control study	Yuan Y, Hou Z, Zhang Z, Bai F, Yu H, You J, Shi Y, Liu W, Jiang T.	J Clin Psychiatry	2010
**63**	Novel diffusion tensor imaging methodology to detect and quantify injured regions and affected brain pathways in traumatic brain injury	Singh M, Jeong J, Hwang D, Sungkarat W, Gruen P.	Magn Reson Imaging	2010
**64**	Genetic influences on brain asymmetry: a DTI study of 374 twins and siblings	Jahanshad N, Lee AD, Barysheva M, McMahon KL, de Zubicaray GI, Martin NG, Wright MJ, Toga AW, Thompson PM.	Neuroimage	2010
**65**	Combining functional and anatomical connectivity reveals brain networks for auditory language comprehension	Saur D, Schelter B, Schnell S, Kratochvil D, Küpper H, Kellmeyer P, Kümmerer D, Klöppel S, Glauche V, Lange R, Mader W, Feess D, Timmer J, Weiller C.	Neuroimage	2010
**66**	Diffusion tensor imaging in autism spectrum disorders:	Jou RJ, Jackowski AP, Papademetris X, Rajeevan N, Staib LH, Volkmar FR.	Aust N Z J Psychiatry	2011

	preliminary evidence of abnormal neural connectivity			
**67**	Selective frontal neurodegeneration of the inferior fronto-occipital fasciculus in progressive supranuclear palsy (PSP) demonstrated by diffusion tensor tractography	Kvickström P, Eriksson B, van Westen D, Lätt J, Elfgren C, Nilsson C.	BMC Neurol	2011
**68**	Alterations in white matter pathways in Angelman syndrome	Peters SU, Kaufmann WE, Bacino CA, Anderson AW, Adapa P, Chu Z, Yallampalli R, Traipe E, Hunter JV, Wilde EA.	Dev Med Child Neurol	2011
**69**	Abnormal language pathway in children with Angelman syndrome	Wilson BJ, Sundaram SK, Huq AH, Jeong JW, Halverson SR, Behen ME, Bui DQ, Chugani HT.	Pediatr Neurol	2011
**70**	The structure and connectivity of semantic memory in the healthy older adult brain	de Zubicaray GI, Rose SE, McMahon KL.	Neuroimage	2011
**71**	Cortex-sparing fiber dissection: an improved method for the study of white matter anatomy in the human brain	Martino J, De Witt Hamer PC, Vergani F, Brogna C, de Lucas EM, Vázquez-Barquero A, García-Porrero JA, Duffau H.	J Anat	2011
**72**	Is the human left middle longitudinal fascicle essential for language? A brain electrostimulation study	De Witt Hamer PC, Moritz-Gasser S, Gatignol P, Duffau H.	Hum Brain Mapp	2011
**73**	Covert face recognition without the fusiform-temporal pathways	Valdés-Sosa M, Bobes MA, Quiñones I, Garcia L, Valdes-Hernandez PA, Iturria Y, Melie-Garcia L, Lopera F, Asencio J.	Neuroimage	2011
**74**	Abnormal frontal cortex white matter connections in bipolar disorder: a DTI tractography study	Lin F, Weng S, Xie B, Wu G, Lei H.	J Affect Disord	2011
**75**	Reduplicative paramnesia after a right frontal lesion	Lee K, Shinbo M, Kanai H, Nagumo Y.	Cogn Behav Neurol	2011
**76**	Extensive abnormality of brain white matter integrity in pathological gambling	Joutsa J, Saunavaara J, Parkkola R, Niemelä S, Kaasinen V.	Psychiatry Res	2011
**77**	T2 lesion location really matters: a 10 year follow-up study in primary progressive multiple sclerosis	Bodini B, Battaglini M, De Stefano N, Khaleeli Z, Barkhof F, Chard D, Filippi M, Montalban X, Polman C, Rovaris M, Rovira A, Samson R, Miller D, Thompson A, Ciccarelli O.	J Neurol Neurosurg Psychiatry	2011
**78**	White matter in autism spectrum disorders - evidence of impaired fiber formation	Bode MK, Mattila ML, Kiviniemi V, Rahko J, Moilanen I, Ebeling H, Tervonen O, Nikkinen J.	Acta Radiol	2011
**79**	Principal eigenvector field segmentation for reproducible diffusion tensor tractography of	Rathore RK, Gupta RK, Agarwal S, Trivedi R, Tripathi RP, Awasthi R.	Magn Reson Imaging	2011

	white matter structures			
**80**	Structural neural phenotype of autism: preliminary evidence from a diffusion tensor imaging study using tract-based spatial statistics	Jou RJ, Mateljevic N, Kaiser MD, Sugrue DR, Volkmar FR, Pelphrey KA.	AJNR Am J Neuroradiol	2011
**81**	Comparison of diffusion tensor image study in association fiber tracts among normal, amnestic mild cognitive impairment, and Alzheimer's patients	Zhang YZ, Chang C, Wei XE, Fu JL, Li WB.	Neurol India	2011
**82**	Changes in gray matter volume and white matter microstructure in adolescents with obsessive-compulsive disorder	Zarei M, Mataix-Cols D, Heyman I, Hough M, Doherty J, Burge L, Winmill L, Nijhawan S, Matthews PM, James A.	Biol Psychiatry	2011
**83**	Diffusion tensor imaging findings in neurologically asymptomatic patients with end stage renal disease	Kim HS, Park JW, Bai DS, Jeong JY, Hong JH, Son SM, Jang SH.	NeuroRehabilitation	2011
**84**	Atlasing location, asymmetry and inter-subject variability of white matter tracts in the human brain with MR diffusion tractography	Thiebaut de Schotten M, Ffytche DH, Bizzi A, Dell'Acqua F, Allin M, Walshe M, Murray R, Williams SC, Murphy DG, Catani M.	Neuroimage	2011
**85**	Microstructural brain injury in post-concussion syndrome after minor head injury	Smits M, Houston GC, Dippel DW, Wielopolski PA, Vernooij MW, Koudstaal PJ, Hunink MG, van der Lugt A.	Neuroradiology	2011
**86**	Sex-linked white matter microstructure of the social and analytic brain	Chou KH, Cheng Y, Chen IY, Lin CP, Chu WC.	Neuroimage	2011
**87**	DTI-MR tractography of white matter damage in stroke patients with neglect	Urbanski M, Thiebaut de Schotten M, Rodrigo S, Oppenheim C, Touzé E, Méder JF, Moreau K, Loeper-Jeny C, Dubois B, Bartolomeo P.	Exp Brain Res	2011
**88**	Electrostimulation mapping of spatial neglect	Roux FE, Dufor O, Lauwers-Cances V, Boukhatem L, Brauge D, Draper L, Lotterie JA, Démonet JF.	Neurosurgery	2011
**89**	Positive symptoms and white matter microstructure in never-medicated first episode schizophrenia	Cheung V, Chiu CP, Law CW, Cheung C, Hui CL, Chan KK, Sham PC, Deng MY, Tai KS, Khong PL, McAlonan GM, Chua SE, Chen E.	Psychol Med	2011
**90**	Neuroanatomical changes due to hearing loss and chronic tinnitus: a combined VBM and DTI study	Husain FT, Medina RE, Davis CW, Szymko-Bennett Y, Simonyan K, Pajor NM, Horwitz B.	Brain Res	2011
**91**	Diffusion tensor imaging and white matter lesions at the subacute stage in mild traumatic brain injury	Messé A, Caplain S, Paradot G, Garrigue D, Mineo JF, Soto Ares G, Ducreux D, Vignaud F, Rozec G, Desal H, Pélégrini-Issac M, Montreuil M, Benali H, Lehéricy S.	Hum Brain Mapp	2011

	with persistent neurobehavioral impairment			
**92**	Tract-specific analyses of diffusion tensor imaging show widespread white matter compromise in autism spectrum disorder	Shukla DK, Keehn B, Müller RA.	J Child Psychol Psychiatry	2011
**93**	White matter microstructure in untreated first episode bipolar disorder with psychosis: comparison with schizophrenia	Lu LH, Zhou XJ, Keedy SK, Reilly JL, Sweeney JA.	Bipolar Disord	2011
**94**	Cerebral network disruption as a possible mechanism for impaired recovery after acute pontine stroke	Förster A, Griebe M, Ottomeyer C, Rossmanith C, Gass A, Kern R, Hennerici MG, Szabo K.	Cerebrovasc Dis	2011
**95**	Longitudinal changes of structural connectivity in traumatic axonal injury	Wang JY, Bakhadirov K, Abdi H, Devous MD Sr, Marquez de la Plata CD, Moore C, Madden CJ, Diaz-Arrastia R.	Neurology	2011
**96**	Voxel-based meta-analysis of regional white-matter volume differences in autism spectrum disorder versus healthy controls	Radua J, Via E, Catani M, Mataix-Cols D.	Psychol Med	2011
**97**	Mean diffusivity and fractional anisotropy as indicators of disease and genetic liability to schizophrenia	Clark KA, Nuechterlein KH, Asarnow RF, Hamilton LS, Phillips OR, Hageman NS, Woods RP, Alger JR, Toga AW, Narr KL.	J Psychiatr Res	2011
**98**	The cholinergic system in mild cognitive impairment and Alzheimer's disease: an in vivo MRI and DTI study	Teipel SJ, Meindl T, Grinberg L, Grothe M, Cantero JL, Reiser MF, Möller HJ, Heinsen H, Hampel H.	Hum Brain Mapp	2011
**99**	Dynamic processing in the human language system: synergy between the arcuate fascicle and extreme capsule	Rolheiser T, Stamatakis EA, Tyler LK.	J Neurosci	2011
**100**	BDNF gene effects on brain circuitry replicated in 455 twins	Chiang MC, Barysheva M, Toga AW, Medland SE, Hansell NK, James MR, McMahon KL, de Zubicaray GI, Martin NG, Wright MJ, Thompson PM.	Neuroimage	2011
**101**	The anatomy of the callosal and visual-association pathways in high-functioning autism: a DTI tractography study	Thomas C, Humphreys K, Jung KJ, Minshew N, Behrmann M.	Cortex	2011
**102**	Predictive value of inferior fronto-occipital fasciculus (IFO) DTI-fiber tracking for determining the extent of resection for	Bertani G, Carrabba G, Raneri F, Fava E, Castellano A, Falini A, Casarotti A, Gaini SM, Bello L.	J Neurosurg Sci	2012

	surgery of frontal and temporal gliomas preoperatively			
**103**	A qualitative and quantitative review of diffusion tensor imaging studies in reading and dyslexia	Vandermosten M, Boets B, Wouters J, Ghesquière P.	Neurosci Biobehav Rev	2012
**104**	A tractography study in dyslexia: neuroanatomic correlates of orthographic, phonological and speech processing	Vandermosten M, Boets B, Poelmans H, Sunaert S, Wouters J, Ghesquière P.	Brain	2012
**105**	Differences in white matter reflect atypical developmental trajectory in autism: A Tract-based Spatial Statistics study	Bakhtiari R, Zürcher NR, Rogier O, Russo B, Hippolyte L, Granziera C, Araabi BN, Nili Ahmadabadi M, Hadjikhani N.	Neuroimage Clin	2012
**106**	Ventral and dorsal visual streams in posterior cortical atrophy: a DT MRI study	Migliaccio R, Agosta F, Scola E, Magnani G, Cappa SF, Pagani E, Canu E, Comi G, Falini A, Gorno-Tempini ML, Bartolomeo P, Filippi M.	Neurobiol Aging	2012
**107**	The neural underpinings of simultanagnosia: disconnecting the visuospatial attention network	Chechlacz M, Rotshtein P, Hansen PC, Riddoch JM, Deb S, Humphreys GW.	J Cogn Neurosci	2012
**108**	White matter microstructural abnormalities in the frontal lobe of adults with antisocial personality disorder	Sundram F, Deeley Q, Sarkar S, Daly E, Latham R, Craig M, Raczek M, Fahy T, Picchioni M; UK AIMS Network; Barker GJ, Murphy DG.	Cortex	2012
**109**	White matter abnormalities in children and adolescents with obsessive-compulsive disorder: a diffusion tensor imaging study	Jayarajan RN, Venkatasubramanian G, Viswanath B, Janardhan Reddy YC, Srinath S, Vasudev MK, Chandrashekar CR.	Depress Anxiety	2012
**110**	Brain white matter organisation in adolescence is related to childhood cerebral responses to facial expressions and harm avoidance	Taddei M, Tettamanti M, Zanoni A, Cappa S, Battaglia M.	Neuroimage	2012
**111**	Correlation between cognitive function and the association fibers in patients with Alzheimer's disease using diffusion tensor imaging	Meng JZ, Guo LW, Cheng H, Chen YJ, Fang L, Qi M, Jia ZY, Mohammed W, Hong XN.	J Clin Neurosci	2012
**112**	Role of frontotemporal fiber tract integrity in task-switching performance of healthy controls and patients with temporal lobe epilepsy	Kucukboyaci NE, Girard HM, Hagler DJ Jr, Kuperman J, Tecoma ES, Iragui VJ, Halgren E, McDonald CR.	J Int Neuropsychol Soc	2012

**113**	Magnetic resonance imaging correlates of first-episode psychosis in young adult male patients: combined analysis of grey and white matter	Ruef A, Curtis L, Moy G, Bessero S, Badan Bâ M, Lazeyras F, Lövblad KO, Haller S, Malafosse A, Giannakopoulos P, Merlo M.	J Psychiatry Neurosci	2012
**114**	Neural tracts injuries in patients with hypoxic ischemic brain injury: diffusion tensor imaging study	Lee AY, Shin DG, Park JS, Hong GR, Chang PH, Seo JP, Jang SH.	Neurosci Lett	2012
**115**	Monkey to human comparative anatomy of the frontal lobe association tracts	Thiebaut de Schotten M, Dell'Acqua F, Valabregue R, Catani M.	Cortex	2012
**116**	The value of diffusion tensor imaging in the differential diagnosis of subcortical ischemic vascular dementia and Alzheimer's disease in patients with only mild white matter alterations on T2-weighted images	Fu JL, Zhang T, Chang C, Zhang YZ, Li WB.	Acta Radiol	2012
**117**	Quantitative evaluation of changes in the selected white matter tracts using diffusion tensor imaging in patients with Alzheimer's disease and mild cognitive impairment	Zimny A, Szewczyk P, Bladowska J, Trypka E, Wojtynska R, Leszek J, Sasiadek M.	Neuroradiol J	2012
**118**	White matter abnormalities in patients with focal cortical dysplasia revealed by diffusion tensor imaging analysis in a voxelwise approach	Fonseca Vde C, Yasuda CL, Tedeschi GG, Betting LE, Cendes F.	Front Neurol	2012
**119**	Reduced white matter fractional anisotropy and clinical symptoms in schizophrenia: a voxel-based diffusion tensor imaging study	Nakamura K, Kawasaki Y, Takahashi T, Furuichi A, Noguchi K, Seto H, Suzuki M.	Psychiatry Res	2012
**120**	White matter integrity and behavioral activation in healthy subjects	Xu J, Kober H, Carroll KM, Rounsaville BJ, Pearlson GD, Potenza MN.	Hum Brain Mapp	2012
**121**	Automated diffusion tensor tractography: implementation and comparison to user-driven tractography	Nucifora PG, Wu X, Melhem ER, Gur RE, Gur RC, Verma R.	Acad Radiol	2012
**122**	A multi-modal investigation of behavioral adjustment: post-error slowing is associated with white matter characteristics	Fjell AM, Westlye LT, Amlien IK, Walhovd KB.	Neuroimage	2012
**123**	Microstructural changes and atrophy in	Sala S, Agosta F, Pagani E, Copetti M, Comi G, Filippi M.	Neurobiol Aging	2012

	brain white matter tracts with aging			
**124**	Structural correlates of facial emotion recognition deficits in Parkinson's disease patients	Baggio HC, Segura B, Ibarretxe-Bilbao N, Valldeoriola F, Marti MJ, Compta Y, Tolosa E, Junqué C.	Neuropsychologia	2012
**125**	Sex differences in white matter development during adolescence: a DTI study	Wang Y, Adamson C, Yuan W, Altaye M, Rajagopal A, Byars AW, Holland SK.	Brain Res	2012
**126**	Relationship between aberrant brain connectivity and clinical features in Angelman Syndrome: a new method using tract based spatial statistics of DTI color-coded orientation maps	Tiwari VN, Jeong JW, Wilson BJ, Behen ME, Chugani HT, Sundaram SK.	Neuroimage	2012
**127**	Neuregulin-1 genotype is associated with structural differences in the normal human brain	Barnes A, Isohanni M, Barnett JH, Pietiläinen O, Veijola J, Miettunen J, Paunio T, Tanskanen P, Ridler K, Suckling J, Bullmore ET, Jones PB, Murray GK.	Neuroimage	2012
**128**	Effects of early-life adversity on white matter diffusivity changes in patients at risk for major depression	Frodl T, Carballedo A, Fagan AJ, Lisiecka D, Ferguson Y, Meaney JF.	J Psychiatry Neurosci	2012
**129**	Voxel-based morphometry and diffusion tensor imaging of the optic pathway in primary open-angle glaucoma: a preliminary study	Zikou AK, Kitsos G, Tzarouchi LC, Astrakas L, Alexiou GA, Argyropoulou MI.	AJNR Am J Neuroradiol	2012
**130**	White matter integrity, language, and childhood onset schizophrenia	Clark K, Narr KL, O'Neill J, Levitt J, Siddarth P, Phillips O, Toga A, Caplan R.	Schizophr Res	2012
**131**	White matter structure and symptom dimensions in obsessive-compulsive disorder	Koch K, Wagner G, Schachtzabel C, Schultz CC, Straube T, Güllmar D, Reichenbach JR, Peikert G, Sauer H, Schlösser RG.	J Psychiatr Res	2012
**132**	Cognitive processing speed in older adults: relationship with white matter integrity	Kerchner GA, Racine CA, Hale S, Wilheim R, Laluz V, Miller BL, Kramer JH.	PLoS One	2012
**133**	Neurotrophic tyrosine kinase polymorphism impacts white matter connections in patients with major depressive disorder	Murphy ML, Carballedo A, Fagan AJ, Morris D, Fahey C, Meaney J, Frodl T.	Biol Psychiatry	2012
**134**	Atlas-based white matter analysis in individuals with velo-cardio-facial syndrome (22q11.2 deletion syndrome) and unaffected siblings	Radoeva PD, Coman IL, Antshel KM, Fremont W, McCarthy CS, Kotkar A, Wang D, Shprintzen RJ, Kates WR.	Behav Brain Funct	2012

**135**	Relationship of a variant in the NTRK1 gene to white matter microstructure in young adults	Braskie MN, Jahanshad N, Stein JL, Barysheva M, Johnson K, McMahon KL, de Zubicaray GI, Martin NG, Wright MJ, Ringman JM, Toga AW, Thompson PM.	J Neurosci	2012
**136**	Abnormal white matter integrity in adolescents with internet addiction disorder: a tract-based spatial statistics study	Lin F, Zhou Y, Du Y, Qin L, Zhao Z, Xu J, Lei H.	PLoS One	2012
**137**	White matter integrity and vulnerability to Alzheimer's disease: preliminary findings and future directions	Gold BT, Johnson NF, Powell DK, Smith CD.	Biochim Biophys Acta	2012
**138**	White matter abnormalities in pediatric obsessive-compulsive disorder	Gruner P, Vo A, Ikuta T, Mahon K, Peters BD, Malhotra AK, Uluğ AM, Szeszko PR.	Neuropsychopharmacology	2012
**139**	Brain networks in posterior cortical atrophy: a single case tractography study and literature review	Migliaccio R, Agosta F, Toba MN, Samri D, Corlier F, de Souza LC, Chupin M, Sharman M, Gorno-Tempini ML, Dubois B, Filippi M, Bartolomeo P.	Cortex	2012
**140**	White matter disruptions in adolescents exposed to childhood maltreatment and vulnerability to psychopathology	Huang H, Gundapuneedi T, Rao U.	Neuropsychopharmacology	2012
**141**	Ventral and dorsal fiber systems for imagined and executed movement	Vry MS, Saur D, Rijntjes M, Umarova R, Kellmeyer P, Schnell S, Glauche V, Hamzei F, Weiller C.	Exp Brain Res	2012
**142**	Aphasia induced by gliomas growing in the ventrolateral frontal region: assessment with diffusion MR tractography, functional MR imaging and neuropsychology	Bizzi A, Nava S, Ferrè F, Castelli G, Aquino D, Ciaraffa F, Broggi G, DiMeco F, Piacentini S.	Cortex	2012
**143**	Frontal terminations for the inferior fronto-occipital fascicle: anatomical dissection, DTI study and functional considerations on a multi-component bundle	Sarubbo S, De Benedictis A, Maldonado IL, Basso G, Duffau H.	Brain Struct Funct	2013
**144**	Spatial analysis of diffusion tensor tractography statistics along the inferior fronto-occipital fasciculus with application in progressive supranuclear palsy	Mårtensson J, Nilsson M, Ståhlberg F, Sundgren PC, Nilsson C, van Westen D, Larsson EM, Lätt J.	MAGMA	2013
**145**	Tract-based diffusion tensor imaging in patients with	Boos HB, Mandl RC, van Haren NE, Cahn W, van Baal GC, Kahn RS, Hulshoff Pol HE.	Eur Neuropsychopharmacol	2013

	schizophrenia and their non-psychotic siblings			
**146**	Fronto-occipital fasciculus, corpus callosum and superior longitudinal fasciculus tract alterations of first-episode, medication-naïve and late-onset panic disorder patients	Lai CH, Wu YT.	J Affect Disord	2013
**147**	Dorsal and ventral pathways in language development	Brauer J, Anwander A, Perani D, Friederici AD.	Brain Lang	2013
**148**	White matter in aphasia: a historical review of the Dejerines' studies	Krestel H, Annoni JM, Jagella C.	Brain Lang	2013
**149**	White matter alteration in metabolic syndrome: diffusion tensor analysis	Shimoji K, Abe O, Uka T, Yasmin H, Kamagata K, Asahi K, Hori M, Nakanishi A, Tamura Y, Watada H, Kawamori R, Aoki S.	Diabetes Care	2013
**150**	White matter structural connectivity underlying semantic processing: evidence from brain damaged patients	Han Z, Ma Y, Gong G, He Y, Caramazza A, Bi Y.	Brain	2013
**151**	Double dissociation between visual recognition and picture naming: a study of the visual language connectivity using tractography and brain stimulation	Gil-Robles S, Carvallo A, Jimenez Mdel M, Gomez Caicoya A, Martinez R, Ruiz-Ocaña C, Duffau H.	Neurosurgery	2013
**152**	White matter microstructure in subjects with attention-deficit/hyperactivity disorder and their siblings	Lawrence KE, Levitt JG, Loo SK, Ly R, Yee V, O'Neill J, Alger J, Narr KL.	J Am Acad Child Adolesc Psychiatry	2013
**153**	Middle longitudinal fasciculus delineation within language pathways: a diffusion tensor imaging study in human	Menjot de Champfleur N, Lima Maldonado I, Moritz-Gasser S, Machi P, Le Bars E, Bonafé A, Duffau H.	Eur J Radiol	2013
**154**	Brain white matter microstructure is associated with susceptibility to motion-induced nausea	Napadow V, Sheehan J, Kim J, Dassatti A, Thurler AH, Surjanhata B, Vangel M, Makris N, Schaechter JD, Kuo B.	Neurogastroenterol Motil	2013
**155**	Mapping the connectivity underlying multimodal (verbal and non-verbal) semantic processing: a brain electrostimulation study	Moritz-Gasser S, Herbet G, Duffau H.	Neuropsychologia	2013
**156**	Evidence of a middle longitudinal fasciculus in the human brain from fiber dissection	Maldonado IL, de Champfleur NM, Velut S, Destrieux C, Zemmoura I, Duffau H.	J Anat	2013

**157**	Emotional prosody and diffusion tensor imaging in children after traumatic brain injury	Schmidt AT, Hanten G, Li X, Wilde EA, Ibarra AP, Chu ZD, Helbling AR, Shah S, Levin HS.	Brain Inj	2013
**158**	Fiber dissection and diffusion tensor imaging tractography study of the temporoparietal fiber intersection area	Martino J, da Silva-Freitas R, Caballero H, Marco de Lucas E, García-Porrero JA, Vázquez-Barquero A.	Neurosurgery	2013
**159**	White matter and visuospatial processing in autism: a constrained spherical deconvolution tractography study	McGrath J, Johnson K, O'Hanlon E, Garavan H, Gallagher L, Leemans A.	Autism Res	2013
**160**	Genetic contributions to changes of fiber tracts of ventral visual stream in 22q11.2 deletion syndrome	Kikinis Z, Makris N, Finn CT, Bouix S, Lucia D, Coleman MJ, Tworog-Dube E, Kikinis R, Kucherlapati R, Shenton ME, Kubicki M.	Brain Imaging Behav	2013
**161**	Evaluation of diffusion-tensor imaging-based global search and tractography for tumor surgery close to the language system	Richter M, Zolal A, Ganslandt O, Buchfelder M, Nimsky C, Merhof D.	PLoS One	2013
**162**	Increased brain white matter axial diffusivity associated with fatigue, pain and hyperalgesia in Gulf War illness	Rayhan RU, Stevens BW, Timbol CR, Adewuyi O, Walitt B, VanMeter JW, Baraniuk JN.	PLoS One	2013
**163**	Quantitative diffusion-tensor tractography of long association tracts in patients with traumatic brain injury without associated findings at routine MR imaging	Brandstack N, Kurki T, Tenovuo O.	Radiology	2013
**164**	Pathways to seeing music: enhanced structural connectivity in colored-music synesthesia	Zamm A, Schlaug G, Eagleman DM, Loui P.	Neuroimage	2013
**165**	Reduced white matter integrity and cognitive deficit in never-medicated chronic schizophrenia: a diffusion tensor study using TBSS	Liu X, Lai Y, Wang X, Hao C, Chen L, Zhou Z, Yu X, Hong N.	Behav Brain Res	2013
**166**	Pathophysiological concepts in mild traumatic brain injury: diffusion tensor imaging related to acute perfusion CT imaging	Metting Z, Cerliani L, Rödiger LA, van der Naalt J.	PLoS One	2013
**167**	Lithium and GSK3-β promoter gene variants influence white matter microstructure in bipolar disorder	Benedetti F, Bollettini I, Barberi I, Radaelli D, Poletti S, Locatelli C, Pirovano A, Lorenzi C, Falini A, Colombo C, Smeraldi E.	Neuropsychopharmacology	2013

**168**	Evaluation of early cerebral metabolic, perfusion and microstructural changes in HCV-positive patients: a pilot study	Bladowska J, Zimny A, Knysz B, Małyszczak K, Kołtowska A, Szewczyk P, Gąsiorowski J, Furdal M, Sąsiadek MJ.	J Hepatol	2013
**169**	Neurometabolic and microstructural alterations following a sports-related concussion in female athletes	Chamard E, Lassonde M, Henry L, Tremblay J, Boulanger Y, De Beaumont L, Théoret H.	Brain Inj	2013
**170**	Longitudinal MRI reveals altered trajectory of brain development during childhood and adolescence in fetal alcohol spectrum disorders	Treit S, Lebel C, Baugh L, Rasmussen C, Andrew G, Beaulieu C.	J Neurosci	2013
**171**	Brain structures associated with executive functions during everyday events in a non-clinical sample	Takeuchi H, Taki Y, Sassa Y, Hashizume H, Sekiguchi A, Fukushima A, Kawashima R.	Brain Struct Funct	2013
**172**	Neuropsychological evidence for the functional role of the uncinate fasciculus in semantic control	Harvey DY, Wei T, Ellmore TM, Hamilton AC, Schnur TT.	Neuropsychologia	2013
**173**	Individual structural differences in left inferior parietal area are associated with schoolchildrens' arithmetic scores	Li Y, Hu Y, Wang Y, Weng J, Chen F.	Front Hum Neurosci	2013
**174**	Individual classification of mild cognitive impairment subtypes by support vector machine analysis of white matter DTI	Haller S, Missonnier P, Herrmann FR, Rodriguez C, Deiber MP, Nguyen D, Gold G, Lovblad KO, Giannakopoulos P.	AJNR Am J Neuroradiol	2013
**175**	Decreased white matter integrity before the onset of delusions in patients with Alzheimer's disease: diffusion tensor imaging	Nakaaki S, Sato J, Torii K, Oka M, Negi A, Nakamae T, Narumoto J, Miyata J, Furukawa TA, Mimura M.	Neuropsychiatr Dis Treat	2013
**176**	Effect of antenatal growth on brain white matter maturation in preterm infants at term using tract-based spatial statistics	Lepomäki V, Matomäki J, Lapinleimu H, Lehtonen L, Haataja L, Komu M, Parkkola R; PIPARI Study Group.	Pediatr Radiol	2013
**177**	Evidence of frontotemporal structural hypoconnectivity in social anxiety disorder: A quantitative fiber tractography study	Baur V, Brühl AB, Herwig U, Eberle T, Rufer M, Delsignore A, Jäncke L, Hänggi J.	Hum Brain Mapp	2013
**178**	Usefulness of visual evaluation of the anterior thalamic	Niida A, Niida R, Kuniyoshi K, Motomura M, Uechi A.	Int J Gen Med	2013

	radiation by diffusion tensor tractography for differentiating between Alzheimer's disease and elderly major depressive disorder patients			
**179**	Damage to the left ventral, arcuate fasciculus and superior longitudinal fasciculus-related pathways induces deficits in object naming, phonological language function and writing, respectively	Shinoura N, Midorikawa A, Onodera T, Tsukada M, Yamada R, Tabei Y, Itoi C, Saito S, Yagi K.	Int J Neurosci	2013
**180**	Case series: fractional anisotropy along the trajectory of selected white matter tracts in adolescents born preterm with ventricular dilation	Myall NJ, Yeom KW, Yeatman JD, Gaman-Bean S, Feldman HM.	J Child Neurol	2013
**181**	Extensive white matter abnormalities in patients with first-episode schizophrenia: a Diffusion Tensor Iimaging (DTI) study	Lee SH, Kubicki M, Asami T, Seidman LJ, Goldstein JM, Mesholam-Gately RI, McCarley RW, Shenton ME.	Schizophr Res	2013
**182**	Neuroimaging changes in the brain in contact versus noncontact sport athletes using diffusion tensor imaging	Gajawelli N, Lao Y, Apuzzo ML, Romano R, Liu C, Tsao S, Hwang D, Wilkins B, Lepore N, Law M.	World Neurosurg	2013
**183**	Lifelong bilingualism contributes to cognitive reserve against white matter integrity declines in aging	Gold BT, Johnson NF, Powell DK.	Neuropsychologia	2013
**184**	Brain connectivity in body dysmorphic disorder compared with controls: a diffusion tensor imaging study	Buchanan BG, Rossell SL, Maller JJ, Toh WL, Brennan S, Castle DJ.	Psychol Med	2013
**185**	Cortical and subcortical mapping of language areas: correlation of functional MRI and tractography in a 3T scanner with intraoperative cortical and subcortical stimulation in patients with brain tumors located in eloquent areas	Jiménez de la Peña M, Gil Robles S, Recio Rodríguez M, Ruiz Ocaña C, Martínez de Vega V.	Radiologia	2013
**186**	Prediction of post-surgical seizure outcome in left mesial temporal lobe epilepsy	Feis DL, Schoene-Bake JC, Elger C, Wagner J, Tittgemeyer M, Weber B.	Neuroimage Clin	2013

**187**	Alterations in frontostriatal pathways in children born very preterm	Duerden EG, Card D, Lax ID, Donner EJ, Taylor MJ.	Dev Med Child Neurol	2013
**188**	White matter hyperintensities, exercise, and improvement in gait speed: does type of gait rehabilitation matter?	Nadkarni NK, Studenski SA, Perera S, Rosano C, Aizenstein HJ, Brach JS, Van Swearingen JM.	J Am Geriatr Soc	2013
**189**	Patterns of dysgraphia in primary progressive aphasia compared to post-stroke aphasia	Faria AV, Crinion J, Tsapkini K, Newhart M, Davis C, Cooley S, Mori S, Hillis AE.	Behav Neurol	2013
**190**	White matter microstructure in body dysmorphic disorder and its clinical correlates	Feusner JD, Arienzo D, Li W, Zhan L, Gadelkarim J, Thompson PM, Leow AD.	Psychiatry Res	2013
**191**	Disconnection mechanism and regional cortical atrophy contribute to impaired processing of facial expressions and theory of mind in multiple sclerosis: a structural MRI study	Mike A, Strammer E, Aradi M, Orsi G, Perlaki G, Hajnal A, Sandor J, Banati M, Illes E, Zaitsev A, Herold R, Guttmann CR, Illes Z.	PLoS One	2013
**192**	Unusual compensatory neural connections following disruption of corpus callosum fibers in a patient with corpus callosum hemorrhage	Jang SH, Yeo SS, Chang MC.	Int J Neurosci	2013
**193**	White matter integrity in physically fit older adults	Tseng BY, Gundapuneedi T, Khan MA, Diaz-Arrastia R, Levine BD, Lu H, Huang H, Zhang R.	Neuroimage	2013
**194**	Neuroanatomical pattern classification in a population-based sample of first-episode schizophrenia	Zanetti MV, Schaufelberger MS, Doshi J, Ou Y, Ferreira LK, Menezes PR, Scazufca M, Davatzikos C, Busatto GF.	Prog Neuropsychopharmacol Biol Psychiatry	2013
**195**	White matter integrity is reduced in bulimia nervosa	Mettler LN, Shott ME, Pryor T, Yang TT, Frank GK.	Int J Eat Disord	2013
**196**	Fronto-parietal dorsal and ventral pathways in the context of different linguistic manipulations	Kellmeyer P, Ziegler W, Peschke C, Juliane E, Schnell S, Baumgaertner A, Weiller C, Saur D.	Brain Lang	2013
**197**	Abnormal temporal lobe white matter as a biomarker for genetic risk of bipolar disorder	Mahon K, Burdick KE, Ikuta T, Braga RJ, Gruner P, Malhotra AK, Szeszko PR.	Biol Psychiatry	2013
**198**	Fiber tract-specific white matter lesion severity Findings in late-life depression and by AGTR1 A1166C genotype	Taylor WD, Zhao Z, Ashley-Koch A, Payne ME, Steffens DC, Krishnan RR, Hauser E, MacFall JR.	Hum Brain Mapp	2013
**199**	Action semantics and movement characteristics engage distinct processing	Hoeren M, Kaller CP, Glauche V, Vry MS, Rijntjes M, Hamzei F, Weiller C.	Exp Brain Res	2013

	streams during the observation of tool use			
**200**	Repeating with the right hemisphere: reduced interactions between phonological and lexical-semantic systems in crossed aphasia?	De-Torres I, Dávila G, Berthier ML, Walsh SF, Moreno-Torres I, Ruiz-Cruces R.	Front Hum Neurosci	2013
**201**	Anatomical substrates of cognitive and clinical dimensions in first episode schizophrenia	Rigucci S, Rossi-Espagnet C, Ferracuti S, De Carolis A, Corigliano V, Carducci F, Mancinelli I, Cicone F, Tatarelli R, Bozzao A, Girardi P, Comparelli A.	Acta Psychiatr Scand	2013
**202**	Q-ball of inferior fronto-occipital fasciculus and beyond	Caverzasi E, Papinutto N, Amirbekian B, Berger MS, Henry RG.	PLoS One	2014
**203**	The role of left inferior fronto-occipital fascicle in verbal perseveration: a brain electrostimulation mapping study	Khan OH, Herbet G, Moritz-Gasser S, Duffau H.	Brain Topogr	2014
**204**	Multi-modal MRI of mild traumatic brain injury	Narayana PA, Yu X, Hasan KM, Wilde EA, Levin HS, Hunter JV, Miller ER, Patel VK, Robertson CS, McCarthy JJ.	Neuroimage Clin	2014
**205**	The anatomy of fronto-occipital connections from early blunt dissections to contemporary tractography	Forkel SJ, Thiebaut de Schotten M, Kawadler JM, Dell'Acqua F, Danek A, Catani M.	Cortex	2014
**206**	Decreased white matter integrity in fronto-occipital fasciculus bundles: relation to visual information processing in alcohol-dependent subjects	Bagga D, Sharma A, Kumari A, Kaur P, Bhattacharya D, Garg ML, Khushu S, Singh N.	Alcohol	2014
**207**	Independent contribution of individual white matter pathways to language function in pediatric epilepsy patients	Paldino MJ, Hedges K, Zhang W.	Neuroimage Clin	2014
**208**	White matter abnormalities and cognitive impairment in early-onset schizophrenia-spectrum disorders	Epstein KA, Cullen KR, Mueller BA, Robinson P, Lee S, Kumra S.	J Am Acad Child Adolesc Psychiatry	2014
**209**	Cerebral correlates of visuospatial neglect: a direct cerebral stimulation study	Vallar G, Bello L, Bricolo E, Castellano A, Casarotti A, Falini A, Riva M, Fava E, Papagno C.	Hum Brain Mapp	2014
**210**	Effect of clozapine on white matter integrity in patients with schizophrenia: a diffusion tensor imaging study	Ozcelik-Eroglu E, Ertugrul A, Oguz KK, Has AC, Karahan S, Yazici MK.	Psychiatry Res	2014
**211**	Microstructural white matter correlates of emotion recognition impairment in	Crespi C, Cerami C, Dodich A, Canessa N, Arpone M, Iannaccone S, Corbo M, Lunetta C, Scola E, Falini A, Cappa SF.	Cortex	2014

	Amyotrophic Lateral Sclerosis			
**212**	Fiber tract associated with autistic traits in healthy adults	Hirose K, Miyata J, Sugihara G, Kubota M, Sasamoto A, Aso T, Fukuyama H, Murai T, Takahashi H.	J Psychiatr Res	2014
**213**	White matter water diffusion changes in primary Sjögren syndrome	Tzarouchi LC, Zikou AK, Tsifetaki N, Astrakas LG, Konitsiotis S, Voulgari P, Drosos A, Argyropoulou MI.	AJNR Am J Neuroradiol	2014
**214**	Impaired empathic abilities and reduced white matter integrity in schizophrenia	Fujino J, Takahashi H, Miyata J, Sugihara G, Kubota M, Sasamoto A, Fujiwara H, Aso T, Fukuyama H, Murai T.	Prog Neuropsychopharmacol Biol Psychiatry	2014
**215**	Relations between white matter maturation and reaction time in childhood	Scantlebury N, Cunningham T, Dockstader C, Laughlin S, Gaetz W, Rockel C, Dickson J, Mabbott D.	J Int Neuropsychol Soc	2014
**216**	Emerging structure-function relations in the developing face processing system	Scherf KS, Thomas C, Doyle J, Behrmann M.	Cereb Cortex	2014
**217**	Separate parts of occipito-temporal white matter fibers are associated with recognition of faces and places	Tavor I, Yablonski M, Mezer A, Rom S, Assaf Y, Yovel G.	Neuroimage	2014
**218**	Neuronal substrates of Corsi Block span: Lesion symptom mapping analyses in relation to attentional competition and spatial bias	Chechlacz M, Rotshtein P, Humphreys GW.	Neuropsychologia	2014
**219**	Clinical correlations of microstructural changes in progressive supranuclear palsy	Tessitore A, Giordano A, Caiazzo G, Corbo D, De Micco R, Russo A, Liguori S, Cirillo M, Esposito F, Tedeschi G.	Neurobiol Aging	2014
**220**	Individual differences in white matter anatomy predict dissociable components of reading skill in adults	Welcome SE, Joanisse MF.	Neuroimage	2014
**221**	A combined DTI and structural MRI study in medicated-naïve chronic schizophrenia	Liu X, Lai Y, Wang X, Hao C, Chen L, Zhou Z, Yu X, Hong N.	Magn Reson Imaging	2014
**222**	Reading skill in adult survivors of childhood brain tumor: a theory-based neurocognitive model	Smith KM, King TZ, Jayakar R, Morris RD.	Neuropsychology	2014
**223**	White matter abnormalities in adolescents with generalized anxiety disorder: a diffusion tensor imaging study	Liao M, Yang F, Zhang Y, He Z, Su L, Li L.	BMC Psychiatry	2014
**224**	Interpersonal competence in young adulthood and right laterality in white matter	De Pisapia N, Serra M, Rigo P, Jager J, Papinutto N, Esposito G, Venuti P, Bornstein MH.	J Cogn Neurosci	2014

**225**	Anatomo-functional study of the temporo-parieto-occipital region: dissection, tractographic and brain mapping evidence from a neurosurgical perspective	De Benedictis A, Duffau H, Paradiso B, Grandi E, Balbi S, Granieri E, Colarusso E, Chioffi F, Marras CE, Sarubbo S.	J Anat	2014
**226**	Altered microstructure within social-cognitive brain networks during childhood in Williams syndrome	Haas BW, Barnea-Goraly N, Sheau KE, Yamagata B, Ullas S, Reiss AL.	Cereb Cortex	2014
**227**	Use of diffusion tensor imaging for evaluating changes in the microstructural integrity of white matter over 3 years in patients with amnesic-type mild cognitive impairment converting to Alzheimer's disease	Fu JL, Liu Y, Li YM, Chang C, Li WB.	J Neuroimaging	2014
**228**	Involuntary switching into the native language induced by electrocortical stimulation of the superior temporal gyrus: a multimodal mapping study	Tomasino B, Marin D, Canderan C, Maieron M, Budai R, Fabbro F, Skrap M.	Neuropsychologia	2014
**229**	Structural connectivity in a single case of progressive prosopagnosia: the role of the right inferior longitudinal fasciculus	Grossi D, Soricelli A, Ponari M, Salvatore E, Quarantelli M, Prinster A, Trojano L.	Cortex	2014
**230**	Neuroanatomical abnormalities and cognitive impairments are shared by adults with attention-deficit/hyperactivity disorder and their unaffected first-degree relatives	Pironti VA, Lai MC, Müller U, Dodds CM, Suckling J, Bullmore ET, Sahakian BJ.	Biol Psychiatry	2014
**231**	Repeatability of quantitative metrics derived from MR diffusion tractography in paediatric patients with epilepsy	Paldino MJ, Hedges K, Rodrigues KM, Barboriak DP.	Br J Radiol	2014
**232**	Neural correlates of progressive reduction of bradykinesia in de novo Parkinson's disease	Lee E, Lee JE, Yoo K, Hong JY, Oh J, Sunwoo MK, Kim JS, Jeong Y, Lee PH, Sohn YH, Kang SY.	Parkinsonism Relat Disord	2014
**233**	White matter connectivity and aerobic fitness in male adolescents	Herting MM, Colby JB, Sowell ER, Nagel BJ.	Dev Cogn Neurosci	2014
**234**	Adverse childhood experiences influence white matter	Benedetti F, Bollettini I, Radaelli D, Poletti S, Locatelli C, Falini A, Smeraldi E, Colombo C.	Psychol Med	2014

	microstructure in patients with bipolar disorder			
**235**	Genome-wide schizophrenia variant at MIR137 does not impact white matter microstructure in healthy participants	Kelly S, Morris DW, Mothersill O, Rose EJ, Fahey C, O'Brien C, O'Hanlon E, Gill M, Corvin AP, Donohoe G.	Neurosci Lett	2014
**236**	Multivariate pattern analysis of DTI reveals differential white matter in individuals with obsessive-compulsive disorder	Li F, Huang X, Tang W, Yang Y, Li B, Kemp GJ, Mechelli A, Gong Q.	Hum Brain Mapp	2014
**237**	Altered white matter microstructure is associated with social cognition and psychotic symptoms in 22q11.2 microdeletion syndrome	Jalbrzikowski M, Villalon-Reina JE, Karlsgodt KH, Senturk D, Chow C, Thompson PM, Bearden CE.	Front Behav Neurosci	2014
**238**	Language-general and - specific white matter microstructural bases for reading	Zhang M, Chen C, Xue G, Lu ZL, Mei L, Xue H, Wei M, He Q, Li J, Dong Q.	Neuroimage	2014
**239**	Delineation of early and later adult onset depression by diffusion tensor imaging	Cheng Y, Xu J, Yu H, Nie B, Li N, Luo C, Li H, Liu F, Bai Y, Shan B, Xu L, Xu X.	PLoS One	2014
**240**	Left hemisphere fractional anisotropy increase in noise-induced tinnitus: a diffusion tensor imaging (DTI) study of white matter tracts in the brain	Benson RR, Gattu R, Cacace AT.	Hear Res	2014
**241**	Brain morphometry of Dravet syndrome	Pérez A, García-Pentón L, Canales-Rodríguez EJ, Lerma-Usabiaga G, Iturria-Medina Y, Román FJ, Davidson D, Alemán-Gómez Y, Acha J, Carreiras M.	Epilepsy Res	2014
**242**	Diffusion tensor tractography in hypothyroidism and its correlation with memory function	Singh S, Trivedi R, Singh K, Kumar P, Shankar LR, Khushu S.	J Neuroendocrinol	2014
**243**	A longitudinal diffusion tensor imaging study assessing white matter fiber tracts after sports-related concussion	Murugavel M, Cubon V, Putukian M, Echemendia R, Cabrera J, Osherson D, Dettwiler A.	J Neurotrauma	2014
**244**	Early frontal structural and functional changes in mild white matter lesions relevant to cognitive decline	Sun X, Liang Y, Wang J, Chen K, Chen Y, Zhou X, Jia J, Zhang Z.	J Alzheimers Dis	2014
**245**	Abnormalities of cortical thickness, subcortical shapes, and white matter integrity in subcortical vascular cognitive impairment	Thong JY, Du J, Ratnarajah N, Dong Y, Soon HW, Saini M, Tan MZ, Ta AT, Chen C, Qiu A.	Hum Brain Mapp	2014
**246**	White matter integrity is associated with	Gold BT, Zhu Z, Brown CA, Andersen AH, LaDu MJ, Tai L, Jicha GA, Kryscio RJ, Estus S, Nelson PT,	Neurobiol Aging	2014

	cerebrospinal fluid markers of Alzheimer's disease in normal adults	Scheff SW, Abner E, Schmitt FA, Van Eldik LJ, Smith CD.		
**247**	White matter changes in preclinical Alzheimer's disease: a magnetic resonance imaging-diffusion tensor imaging study on cognitively normal older people with positive amyloid β protein 42 levels	Molinuevo JL, Ripolles P, Simó M, Lladó A, Olives J, Balasa M, Antonell A, Rodriguez-Fornells A, Rami L.	Neurobiol Aging	2014
**248**	Association between white matter fiber structure and reward-related reactivity of the ventral striatum	Koch K, Wagner G, Schachtzabel C, Schultz CC, Güllmar D, Reichenbach JR, Sauer H, Zimmer C, Schlösser RG.	Hum Brain Mapp	2014
**249**	DTI-measured white matter abnormalities in adolescents with Conduct Disorder	Haney-Caron E, Caprihan A, Stevens MC.	J Psychiatr Res	2014
**250**	Regional neuronal network failure and cognition in late-onset sporadic Alzheimer disease	Carter SF, Embleton KV, Anton-Rodriguez JM, Burns A, Ralph MA, Herholz K.	AJNR Am J Neuroradiol	2014
**251**	FIBRASCAN: a novel method for 3D white matter tract reconstruction in MR space from cadaveric dissection	Zemmoura I, Serres B, Andersson F, Barantin L, Tauber C, Filipiak I, Cottier JP, Venturini G, Destrieux C.	Neuroimage	2014
**252**	Fiber pathways connecting cortical areas relevant for spatial orienting and exploration	Suchan J, Umarova R, Schnell S, Himmelbach M, Weiller C, Karnath HO, Saur D.	Hum Brain Mapp	2014
**253**	The multimodal connectivity of the hippocampal complex in auditory and visual hallucinations	Amad A, Cachia A, Gorwood P, Pins D, Delmaire C, Rolland B, Mondino M, Thomas P, Jardri R.	Mol Psychiatry	2014
**254**	Road work on memory lane--functional and structural alterations to the learning and memory circuit in adults born very preterm	Salvan P, Froudist Walsh S, Allin MP, Walshe M, Murray RM, Bhattacharyya S, McGuire PK, Williams SC, Nosarti C.	Neuroimage	2014
**255**	Regional but not global brain damage contributes to fatigue in multiple sclerosis	Rocca MA, Parisi L, Pagani E, Copetti M, Rodegher M, Colombo B, Comi G, Falini A, Filippi M.	Radiology	2014
**256**	Association of dorsal inferior frontooccipital fasciculus fibers in the deep parietal lobe with both reading and writing processes: a brain mapping study	Motomura K, Fujii M, Maesawa S, Kuramitsu S, Natsume A, Wakabayashi T.	J Neurosurg	2014
**257**	Age-related differences in white matter tract microstructure are	Peters BD, Ikuta T, DeRosse P, John M, Burdick KE, Gruner P, Prendergast DM, Szeszko PR, Malhotra AK.	Biol Psychiatry	2014

	associated with cognitive performance from childhood to adulthood			
**258**	Structural brain changes related to bilingualism: does immersion make a difference?	Stein M, Winkler C, Kaiser A, Dierks T.	Front Psychol	2014
**259**	The left inferior fronto-occipital fasciculus subserves language semantics: a multilevel lesion study	Almairac F, Herbet G, Moritz-Gasser S, de Champfleur NM, Duffau H.	Brain Struct Funct	2015
**260**	Not on speaking terms: hallucinations and structural network disconnectivity in schizophrenia	Ćurčić-Blake B, Nanetti L, van der Meer L, Cerliani L, Renken R, Pijnenborg GH, Aleman A.	Brain Struct Funct	2015
**261**	In vivo tractography of fetal association fibers	Mitter C, Prayer D, Brugger PC, Weber M, Kasprian G.	PLoS One	2015
**262**	Structural alterations of the language connectome in children with specific language impairment	Vydrova R, Komarek V, Sanda J, Sterbova K, Jahodova A, Maulisova A, Zackova J, Reissigova J, Krsek P, Kyncl M.	Brain Lang	2015
**263**	Higher integrity of the motor and visual pathways in long-term video game players	Zhang Y, Du G, Yang Y, Qin W, Li X, Zhang Q.	Front Hum Neurosci	2015
**264**	Gray- and white-matter anatomy of absolute pitch possessors	Dohn A, Garza-Villarreal EA, Chakravarty MM, Hansen M, Lerch JP, Vuust P.	Cereb Cortex	2015
**265**	White matter fractional anisotropy over two time points in early onset schizophrenia and adolescent cannabis use disorder: A naturalistic diffusion tensor imaging study	Epstein KA, Kumra S.	Psychiatry Res	2015
**266**	Location of brain tumor intersecting white matter tracts predicts patient prognosis	Mickevicius NJ, Carle AB, Bluemel T, Santarriaga S, Schloemer F, Shumate D, Connelly J, Schmainda KM, LaViolette PS.	J Neurooncol	2015
**267**	Abnormal white matter integrity in chronic users of codeine-containing cough syrups: a tract-based spatial statistics study	Qiu YW, Su HH, Lv XF, Jiang GH.	AJNR Am J Neuroradiol	2015
**268**	Towards a functional atlas of human white matter	Sarubbo S, De Benedictis A, Merler S, Mandonnet E, Balbi S, Granieri E, Duffau H.	Hum Brain Mapp	2015
**269**	Alterations in white matter integrity in first-episode, treatment-naive patients with somatization disorder	Zhang J, Jiang M, Yao D, Dai Y, Long L, Yu M, Liu J, Zhang Z, Xiao C, Guo W.	Neurosci Lett	2015
**270**	White matter and reading deficits after	Johnson CP, Juranek J, Swank PR, Kramer L, Cox CS Jr, Ewing-Cobbs L.	Neuroimage Clin	2015

	pediatric traumatic brain injury: A diffusion tensor imaging study			
**271**	Tractography of Association Fibers Associated with Language Processing	Egger K, Yang S, Reisert M, Kaller C, Mader I, Beume L, Weiller C, Urbach H.	Clin Neuroradiol	2015
**272**	A disconnection account of subjective empathy impairments in diffuse low-grade glioma patients	Herbet G, Lafargue G, Moritz-Gasser S, Menjot de Champfleur N, Costi E, Bonnetblanc F, Duffau H.	Neuropsychologia	2015
**273**	Decreased and Increased Anisotropy along Major Cerebral White Matter Tracts in Preterm Children and Adolescents	Travis KE, Adams JN, Ben-Shachar M, Feldman HM.	PLoS One	2015
**274**	Subcortical anatomy as an anatomical and functional landmark in insulo-opercular gliomas: implications for surgical approach to the insular region	Martino J, Mato D, de Lucas EM, García-Porrero JA, Gabarrós A, Fernández-Coello A, Vázquez-Barquero A.	J Neurosurg	2015
**275**	The course and the anatomo-functional relationships of the optic radiation: a combined study with 'post mortem' dissections and 'in vivo' direct electrical mapping	Sarubbo S, De Benedictis A, Milani P, Paradiso B, Barbareschi M, Rozzanigo U, Colarusso E, Tugnoli V, Farneti M, Granieri E, Duffau H, Chioffi F.	J Anat	2015
**276**	Tract-based spatial statistics analysis of white matter changes in children with anisometropic amblyopia	Li Q, Zhai L, Jiang Q, Qin W, Li Q, Yin X, Guo M.	Neurosci Lett	2015
**277**	Investigating the ventral-lexical, dorsal-sublexical model of basic reading processes using diffusion tensor imaging	Cummine J, Dai W, Borowsky R, Gould L, Rollans C, Boliek C.	Brain Struct Funct	2015
**278**	Asymmetrical white matter networks for attending to global versus local features	Chechlacz M, Mantini D, Gillebert CR, Humphreys GW.	Cortex	2015
**279**	White Matter Differences Among Adolescents Reporting Psychotic Experiences: A Population-Based Diffusion Magnetic Resonance Imaging Study	O'Hanlon E, Leemans A, Kelleher I, Clarke MC, Roddy S, Coughlan H, Harley M, Amico F, Hoscheit MJ, Tiedt L, Tabish J, McGettigan A, Frodl T, Cannon M.	JAMA Psychiatry	2015
**280**	Tract specific analysis in patients with sickle cell disease	Chai Y, Coloigner J, Qu X, Choi S, Bush A, Borzage M, Vu C, Lepore N, Wood J.	Proc SPIE Int Soc Opt Eng	2015
**281**	Applying a free-water correction to diffusion imaging data uncovers stress-related neural	Bergamino M, Pasternak O, Farmer M, Shenton ME, Hamilton JP.	Neuroimage Clin	2015

	pathology in depression			
**282**	Facial affect recognition linked to damage in specific white matter tracts in traumatic brain injury	Genova HM, Rajagopalan V, Chiaravalloti N, Binder A, Deluca J, Lengenfelder J.	Soc Neurosci	2015
**283**	White matter microstructure and the variable adult outcome of childhood attention deficit hyperactivity disorder	Shaw P, Sudre G, Wharton A, Weingart D, Sharp W, Sarlls J.	Neuropsychopharmacology	2015
**284**	Regional vulnerability of longitudinal cortical association connectivity: Associated with structural network topology alterations in preterm children with cerebral palsy	Ceschin R, Lee VK, Schmithorst V, Panigrahy A.	Neuroimage Clin	2015
**285**	Local but not long-range microstructural differences of the ventral temporal cortex in developmental prosopagnosia	Song S, Garrido L, Nagy Z, Mohammadi S, Steel A, Driver J, Dolan RJ, Duchaine B, Furl N.	Neuropsychologia	2015
**286**	Comparison of different stimulation parameters of repetitive transcranial magnetic stimulation for unilateral spatial neglect in stroke patients	Yang W, Liu TT, Song XB, Zhang Y, Li ZH, Cui ZH, Hao Q, Liu HL, Lei CL, Liu J.	J Neurol Sci	2015
**287**	Structural Variability within Frontoparietal Networks and Individual Differences in Attentional Functions: An Approach Using the Theory of Visual Attention	Chechlacz M, Gillebert CR, Vangkilde SA, Petersen A, Humphreys GW.	J Neurosci	2015
**288**	Secure attachment status is associated with white matter integrity in healthy young adults	Serra M, De Pisapia N, Rigo P, Papinutto N, Jager J, Bornstein MH, Venuti P.	Neuroreport	2015
**289**	Sterol Regulatory Element Binding Transcription Factor-1 Gene Variation and Medication Load Influence White Matter Structure in Schizophrenia	Bollettini I, Barberi I, Poletti S, Radaelli D, Pirovano A, Lorenzi C, Falini A, Cavallaro R, Smeraldi E, Benedetti F.	Neuropsychobiology	2015
**290**	Double-letter processing in surface dyslexia and dysgraphia following a left temporal lesion: A multimodal neuroimaging study	Tomasino B, Marin D, Maieron M, D'Agostini S, Fabbro F, Skrap M, Luzzatti C.	Cortex	2015

**291**	Constrained spherical deconvolution-based tractography and tract-based spatial statistics show abnormal microstructural organization in Asperger syndrome	Roine U, Salmi J, Roine T, Wendt TN, Leppämäki S, Rintahaka P, Tani P, Leemans A, Sams M.	Mol Autism	2015
**292**	White matter abnormalities in major depressive disorder with melancholic and atypical features: A diffusion tensor imaging study	Ota M, Noda T, Sato N, Hattori K, Hori H, Sasayama D, Teraishi T, Nagashima A, Obu S, Higuchi T, Kunugi H.	Psychiatry Clin Neurosci	2015
**293**	White matter microstructure in bipolar disorder is influenced by the serotonin transporter gene polymorphism 5-HTTLPR	Benedetti F, Bollettini I, Poletti S, Locatelli C, Lorenzi C, Pirovano A, Smeraldi E, Colombo C.	Genes Brain Behav	2015
**294**	Cognitive performances associate with measures of white matter integrity in bipolar disorder	Poletti S, Bollettini I, Mazza E, Locatelli C, Radaelli D, Vai B, Smeraldi E, Colombo C, Benedetti F.	J Affect Disord	2015
**295**	Distinct loci of lexical and semantic access deficits in aphasia: Evidence from voxel-based lesion-symptom mapping and diffusion tensor imaging	Harvey DY, Schnur TT.	Cortex	2015
**296**	Adverse childhood experiences influence white matter microstructure in patients with schizophrenia	Poletti S, Mazza E, Bollettini I, Locatelli C, Cavallaro R, Smeraldi E, Benedetti F.	Psychiatry Res	2015
**297**	The ins and outs of meaning: Behavioral and neuroanatomical dissociation of semantically-driven word retrieval and multimodal semantic recognition in aphasia	Mirman D, Zhang Y, Wang Z, Coslett HB, Schwartz MF.	Neuropsychologia	2015
**298**	Asymmetry of White Matter Pathways in Developing Human Brains	Song JW, Mitchell PD, Kolasinski J, Ellen Grant P, Galaburda AM, Takahashi E.	Cereb Cortex	2015
**299**	Diffuse alterations in grey and white matter associated with cognitive impairment in Shwachman-Diamond syndrome: evidence from a multimodal approach	Perobelli S, Alessandrini F, Zoccatelli G, Nicolis E, Beltramello A, Assael BM, Cipolli M.	Neuroimage Clin	2015
**300**	Widespread white matter tract aberrations in youth with familial risk for bipolar disorder	Roybal DJ, Barnea-Goraly N, Kelley R, Bararpour L, Howe ME, Reiss AL, Chang KD.	Psychiatry Res	2015

**301**	Brain white matter microstructure in deficit and non-deficit subtypes of schizophrenia	Spalletta G, De Rossi P, Piras F, Iorio M, Dacquino C, Scanu F, Girardi P, Caltagirone C, Kirkpatrick B, Chiapponi C.	Psychiatry Res	2015
**302**	White matter changes in first episode psychosis and their relation to the size of sample studied: a DTI study	Melicher T, Horacek J, Hlinka J, Spaniel F, Tintera J, Ibrahim I, Mikolas P, Novak T, Mohr P, Hoschl C.	Schizophr Res	2015
**303**	Differential susceptibility of white matter tracts to inflammatory mediators in schizophrenia: an integrated DTI study	Prasad KM, Upton CH, Nimgaonkar VL, Keshavan MS.	Schizophr Res	2015
**304**	White matter integrity and depressive symptoms in cerebral small vessel disease: The RUN DMC study	van Uden IW, Tuladhar AM, de Laat KF, van Norden AG, Norris DG, van Dijk EJ, Tendolkar I, de Leeuw FE.	Am J Geriatr Psychiatry	2015
**305**	Mapping pathological changes in brain structure by combining T1- and T2-weighted MR imaging data	Ganzetti M, Wenderoth N, Mantini D.	Neuroradiology	2015
**306**	Evidence from structural and diffusion tensor imaging for frontotemporal deficits in psychometric schizotypy	DeRosse P, Nitzburg GC, Ikuta T, Peters BD, Malhotra AK, Szeszko PR.	Schizophr Bull	2015
**307**	White matter microstructure among youth with perinatally acquired HIV is associated with disease severity	Uban KA, Herting MM, Williams PL, Ajmera T, Gautam P, Huo Y, Malee KM, Yogev R, Csernansky JG, Wang L, Nichols SL, Sowell ER; Pediatric HIVAIDS Cohort and the Pediatric Imaging, Neurocognition, and Genetics Studies.	AIDS	2015
**308**	Functionally relevant white matter degradation in multiple sclerosis: a tract-based spatial meta-analysis	Welton T, Kent D, Constantinescu CS, Auer DP, Dineen RA.	Radiology	2015
**309**	Connectivity-based whole brain dual parcellation by group ICA reveals tract structures and decreased connectivity in schizophrenia	Wu L, Calhoun VD, Jung RE, Caprihan A.	Hum Brain Mapp	2015
**310**	Disruption of white matter integrity marks poor antidepressant response in bipolar disorder	Bollettini I, Poletti S, Locatelli C, Vai B, Smeraldi E, Colombo C, Benedetti F.	J Affect Disord	2015
**311**	Altered Structural and Functional Connectivity in Late Preterm Preadolescence: An Anatomic Seed-Based Study of Resting State Networks Related to	Degnan AJ, Wisnowski JL, Choi S, Ceschin R, Bhushan C, Leahy RM, Corby P, Schmithorst VJ, Panigrahy A.	PLoS One	2015

	the Posteromedial and Lateral Parietal Cortex			
**312**	Diffusion tensor imaging and tract-based spatial statistics analysis in Friedreich's ataxia patients	Vieira Karuta SC, Raskin S, de Carvalho Neto A, Gasparetto EL, Doring T, Teive HA.	Parkinsonism Relat Disord	2015
**313**	White Matter Microstructural Integrity Is Associated with Executive Function and Processing Speed in Older Adults with Coronary Artery Disease	Santiago C, Herrmann N, Swardfager W, Saleem M, Oh PI, Black SE, Lanctôt KL.	Am J Geriatr Psychiatry	2015
**314**	Visual-motor deficits relate to altered gray and white matter in young adults born preterm with very low birth weight	Sripada K, Løhaugen GC, Eikenes L, Bjørlykke KM, Håberg AK, Skranes J, Rimol LM.	Neuroimage	2015
**315**	Effects of a Balanced Translocation between Chromosomes 1 and 11 Disrupting the DISC1 Locus on White Matter Integrity	Whalley HC, Dimitrova R, Sprooten E, Dauvermann MR, Romaniuk L, Duff B, Watson AR, Moorhead B, Bastin M, Semple SI, Giles S, Hall J, Thomson P, Roberts N, Hughes ZA, Brandon NJ, Dunlop J, Whitcher B, Blackwood DH, McIntosh AM, Lawrie SM.	PLoS One	2015
**316**	Evaluation of the Degradation of the Selected Projectile, Commissural and Association White Matter Tracts Within Normal Appearing White Matter in Patients with Multiple Sclerosis Using Diffusion Tensor MR Imaging - a Preliminary Study	Banaszek A, Bladowska J, Pokryszko-Dragan A, Podemski R, Sąsiadek MJ.	Pol J Radiol	2015
**317**	Intraoperative subcortical mapping of a language-associated deep frontal tract connecting the superior frontal gyrus to Broca's area in the dominant hemisphere of patients with glioma	Fujii M, Maesawa S, Motomura K, Futamura M, Hayashi Y, Koba I, Wakabayashi T.	J Neurosurg	2015
**318**	Reduced white matter integrity and verbal fluency impairment in young adults with bipolar disorder: a diffusion tensor imaging study	Bauer IE, Ouyang A, Mwangi B, Sanches M, Zunta-Soares GB, Keefe RS, Huang H, Soares JC.	J Psychiatr Res	2015
**319**	Subcomponents and Connectivity of the Inferior Fronto-Occipital Fasciculus Revealed by Diffusion Spectrum Imaging Fiber Tracking	Wu Y, Sun D, Wang Y, Wang Y.	Front Neuroanat	2016

**320**	Phenotypic and Genetic Correlations Between the Lobar Segments of the Inferior Fronto-occipital Fasciculus and Attention	Leng Y, Shi Y, Yu Q, Van Horn JD, Tang H, Li J, Xu W, Ge X, Tang Y, Han Y, Zhang D, Xiao M, Zhang H, Pang Z, Toga AW, Liu S.	Sci Rep	2016
**321**	Cortical Terminations of the Inferior Fronto-Occipital and Uncinate Fasciculi: Anatomical Stem-Based Virtual Dissection	Hau J, Sarubbo S, Perchey G, Crivello F, Zago L, Mellet E, Jobard G, Joliot M, Mazoyer BM, Tzourio-Mazoyer N, Petit L.	Front Neuroanat	2016
**322**	Inferior fronto-temporo-occipital connectivity: a missing link between maltreated girls and neglectful mothers	Rodrigo MJ, León I, Góngora D, Hernández-Cabrera JA, Byrne S, Bobes MA.	Soc Cogn Affect Neurosci	2016
**323**	Quantitative Mapping of Human Brain Vertical-Occipital Fasciculus	Keser Z, Ucisik-Keser FE, Hasan KM.	J Neuroimaging	2016
**324**	Individualized Map of White Matter Pathways: Connectivity-Based Paradigm for Neurosurgical Planning	Tunç B, Ingalhalikar M, Parker D, Lecoeur J, Singh N, Wolf RL, Macyszyn L, Brem S, Verma R.	Neurosurgery	2016
**325**	Individual differences in white matter microstructure predict semantic control	Nugiel T, Alm KH, Olson IR.	Cogn Affect Behav Neurosci	2016
**326**	White Matter Abnormalities in Autism and Unaffected Siblings	Jou RJ, Reed HE, Kaiser MD, Voos AC, Volkmar FR, Pelphrey KA.	J Neuropsychiatry Clin Neurosci	2016
**327**	Abnormal white matter integrity as a structural endophenotype for bipolar disorder	Sarıçiçek A, Zorlu N, Yalın N, Hıdıroğlu C, Çavuşoğlu B, Ceylan D, Ada E, Tunca Z, Özerdem A.	Psychol Med	2016
**328**	White matter connectivity and Internet gaming disorder	Jeong BS, Han DH, Kim SM, Lee SW, Renshaw PF.	Addict Biol	2016
**329**	Decreased integrity of the fronto-temporal fibers of the left inferior occipito-frontal fasciculus associated with auditory verbal hallucinations in schizophrenia	Oestreich LK, McCarthy-Jones S; Australian Schizophrenia Research Bank; Whitford TJ.	Brain Imaging Behav	2016
**330**	Abnormal white matter integrity in synthetic cannabinoid users	Zorlu N, Angelique Di Biase M, Kalaycı ÇÇ, Zalesky A, Bağcı B, Oğuz N, Gelal F, Beşiroğlu L, Gülseren Ş, Sarıçiçek A, Bora E, Pantelis C.	Eur Neuropsychopharmacol	2016
**331**	Impact of reading habit on white matter structure: Cross-sectional and longitudinal analyses	Takeuchi H, Taki Y, Hashizume H, Asano K, Asano M, Sassa Y, Yokota S, Kotozaki Y, Nouchi R, Kawashima R.	Neuroimage	2016
**332**	Characterizing White Matter Tract	Madhavan A, Schwarz CG, Duffy JR, Strand EA, Machulda MM, Drubach DA, Kantarci K,	J Alzheimers Dis	2016

	Degeneration in Syndromic Variants of Alzheimer's Disease: A Diffusion Tensor Imaging Study	Przybelski SA, Reid RI, Senjem ML, Gunter JL, Apostolova LG, Lowe VJ, Petersen RC, Jack CR, Josephs KA, Whitwell JL.		
**333**	Development of human white matter fiber pathways: From newborn to adult ages	Cohen AH, Wang R, Wilkinson M, MacDonald P, Lim AR, Takahashi E.	Int J Dev Neurosci	2016
**334**	Intertemporal choice behavior is constrained by brain structure in healthy participants and pathological gamblers	Mohammadi B, Hammer A, Miedl SF, Wiswede D, Marco-Pallarés J, Herrmann M, Münte TF.	Brain Struct Funct	2016
**335**	The White Matter Microintegrity Alterations of Neocortical and Limbic Association Fibers in Major Depressive Disorder and Panic Disorder: The Comparison	Lai CH, Wu YT.	Medicine (Baltimore)	2016
**336**	Combined DTI Tractography and Functional MRI Study of the Language Connectome in Healthy Volunteers: Extensive Mapping of White Matter Fascicles and Cortical Activations	Vassal F, Schneider F, Boutet C, Jean B, Sontheimer A, Lemaire JJ.	PLoS One	2016
**337**	Brain white matter plasticity and functional reorganization underlying the central pathogenesis of trigeminal neuralgia	Tian T, Guo L, Xu J, Zhang S, Shi J, Liu C, Qin Y, Zhu W.	Sci Rep	2016
**338**	Association of abnormal white matter integrity in the acute phase of motor vehicle accidents with post-traumatic stress disorder	Hu H, Zhou Y, Wang Q, Su S, Qiu Y, Ge J, Wang Z, Xiao Z.	J Affect Disord	2016
**339**	Altered hemispheric lateralization of white matter pathways in developmental dyslexia: Evidence from spherical deconvolution tractography	Zhao J, Thiebaut de Schotten M, Altarelli I, Dubois J, Ramus F.	Cortex	2016
**340**	Blindness alters the microstructure of the ventral but not the dorsal visual stream	Reislev NL, Kupers R, Siebner HR, Ptito M, Dyrby TB.	Brain Struct Funct	2016
**341**	Progressive brain changes in patients with chronic fatigue syndrome: A longitudinal MRI study	Shan ZY, Kwiatek R, Burnet R, Del Fante P, Staines DR, Marshall-Gradisnik SM, Barnden LR.	J Magn Reson Imaging	2016
**342**	Variation in White Matter Connectivity	Unger A, Alm KH, Collins JA, O'Leary JM, Olson IR.	J Int Neuropsychol Soc	2016

	Predicts the Ability to Remember Faces and Discriminate Their Emotions			
**343**	Dysfunctional decision-making related to white matter alterations in bipolar I disorder	Scholz V, Houenou J, Kollmann B, Duclap D, Poupon C, Wessa M.	J Affect Disord	2016
**344**	Aberrant structural and functional connectivity in the salience network and central executive network circuit in schizophrenia	Chen Q, Chen X, He X, Wang L, Wang K, Qiu B.	Neurosci Lett	2016
**345**	Right Cortical and Axonal Structures Eliciting Ocular Deviation During Electrical Stimulation Mapping in Awake Patients	Montemurro N, Herbet G, Duffau H.	Brain Topogr	2016
**346**	Subconcussive Head Impact Exposure and White Matter Tract Changes over a Single Season of Youth Football	Bahrami N, Sharma D, Rosenthal S, Davenport EM, Urban JE, Wagner B, Jung Y, Vaughan CG, Gioia GA, Stitzel JD, Whitlow CT, Maldjian JA.	Radiology	2016
**347**	Relationship between white matter integrity and serum cortisol levels in drug-naive patients with major depressive disorder: diffusion tensor imaging study using tract-based spatial statistics	Liu X, Watanabe K, Kakeda S, Yoshimura R, Abe O, Ide S, Hayashi K, Katsuki A, Umene-Nakano W, Watanabe R, Ueda I, Nakamura J, Korogi Y.	Br J Psychiatry	2016
**348**	Structural white-matter connections mediating distinct behavioral components of spatial neglect in right brain-damaged patients	Vaessen MJ, Saj A, Lovblad KO, Gschwind M, Vuilleumier P.	Cortex	2016
**349**	Testing the connections within face processing circuitry in Capgras delusion with diffusion imaging tractography	Bobes MA, Góngora D, Valdes A, Santos Y, Acosta Y, Fernandez Garcia Y, Lage A, Valdés-Sosa M.	Neuroimage Clin	2016
**350**	White matter microstructure in ultra-high risk and first episode schizophrenia: A prospective study	Rigucci S, Santi G, Corigliano V, Imola A, Rossi-Espagnet C, Mancinelli I, De Pisa E, Manfredi G, Bozzao A, Carducci F, Girardi P, Comparelli A.	Psychiatry Res Neuroimaging	2016
**351**	The Dorsal Rather than Ventral Pathway Better Reflects Individual Syntactic Abilities in Second Language	Yamamoto K, Sakai KL.	Front Hum Neurosci	2016
**352**	Connectomic Insights into Topologically Centralized Network	Xia M, Lin Q, Bi Y, He Y.	Front Hum Neurosci	2016

	Edges and Relevant Motifs in the Human Brain			
**353**	Investigating Connectivity of Orbitomedial Prefrontal Region in a Patient with Traumatic Brain Injury	Hepdurgun C, Özalay Ö, Eroğlu S, Polat Nazlı I, Kitiş Ö, Gönül AS.	Turk Psikiyatri Derg	2016
**354**	Microstructure and Cerebral Blood Flow within White Matter of the Human Brain: A TBSS Analysis	Giezendanner S, Fisler MS, Soravia LM, Andreotti J, Walther S, Wiest R, Dierks T, Federspiel A.	PLoS One	2016
**355**	Abnormal white matter integrity in rapists as indicated by diffusion tensor imaging	Chen CY, Raine A, Chou KH, Chen IY, Hung D, Lin CP.	BMC Neurosci	2016
**356**	Diffusion tensor imaging in children with tuberous sclerosis complex: tract-based spatial statistics assessment of brain microstructural changes	Zikou AK, Xydis VG, Astrakas LG, Nakou I, Tzarouchi LC, Tzoufi M, Argyropoulou MI.	Pediatr Radiol	2016
**357**	Similar white matter but opposite grey matter changes in schizophrenia and high-functioning autism	Katz J, d'Albis MA, Boisgontier J, Poupon C, Mangin JF, Guevara P, Duclap D, Hamdani N, Petit J, Monnet D, Le Corvoisier P, Leboyer M, Delorme R, Houenou J.	Acta Psychiatr Scand	2016
**358**	The right hemisphere is dominant in organization of visual search-A study in stroke patients	Ten Brink AF, Biesbroek JM, Kuijf HJ, Van der Stigchel S, Oort Q, Visser-Meily JM, Nijboer TC.	Behav Brain Res	2016
**359**	The COMT Val158Met polymorphism moderates the association between cognitive functions and white matter microstructure in schizophrenia	Poletti S, Mazza E, Bollettini I, Falini A, Smeraldi E, Cavallaro R, Benedetti F.	Psychiatr Genet	2016
**360**	Neuroanatomical correlates of verbal fluency in early Alzheimer's disease and normal aging	Rodríguez-Aranda C, Waterloo K, Johnsen SH, Eldevik P, Sparr S, Wikran GC, Herder M, Vangberg TR.	Brain Lang	2016
**361**	Frontotemporal networks and behavioral symptoms in primary progressive aphasia	D'Anna L, Mesulam MM, Thiebaut de Schotten M, Dell'Acqua F, Murphy D, Wieneke C, Martersteck A, Cobia D, Rogalski E, Catani M.	Neurology	2016
**362**	Diffusion tensor imaging of idiopathic normal-pressure hydrocephalus and the cerebrospinal fluid tap test	Kang K, Yoon U, Choi W, Lee HW.	J Neurol Sci	2016
**363**	Disrupted white matter connectivity underlying developmental	Cui Z, Xia Z, Su M, Shu H, Gong G.	Hum Brain Mapp	2016

	dyslexia: A machine learning approach			
**364**	Alterations of functional and structural connectivity of freezing of gait in Parkinson's disease	Wang M, Jiang S, Yuan Y, Zhang L, Ding J, Wang J, Zhang J, Zhang K, Wang J.	J Neurol	2016
**365**	Delayed early developmental trajectories of white matter tracts of functional pathways in preterm-born infants: Longitudinal diffusion tensor imaging data	Chang L, Akazawa K, Yamakawa R, Hayama S, Buchthal S, Alicata D, Andres T, Castillo D, Oishi K, Skranes J, Ernst T, Oishi K.	Data Brief	2016
**366**	Gray and white matter imaging: A biomarker for cognitive impairment in early Parkinson's disease?	Duncan GW, Firbank MJ, Yarnall AJ, Khoo TK, Brooks DJ, Barker RA, Burn DJ, O'Brien JT.	Mov Disord	2016
**367**	Fatigue in multiple sclerosis: The contribution of occult white matter damage	Bisecco A, Caiazzo G, d'Ambrosio A, Sacco R, Bonavita S, Docimo R, Cirillo M, Pagani E, Filippi M, Esposito F, Tedeschi G, Gallo A.	Mult Scler	2016
**368**	Early cerebral volume reductions and their associations with reduced lupus disease activity in patients with newly-diagnosed systemic lupus erythematosus	Mak A, Ho RC, Tng HY, Koh HL, Chong JS, Zhou J.	Sci Rep	2016
**369**	Exploratory analysis of diffusion tensor imaging in children with attention deficit hyperactivity disorder: evidence of abnormal white matter structure	Pastura G, Doering T, Gasparetto EL, Mattos P, Araújo AP.	Atten Defic Hyperact Disord	2016
**370**	Age and Sex Effects on White Matter Tracts in Psychosis from Adolescence through Middle Adulthood	Schwehm A, Robinson DG, Gallego JA, Karlsgodt KH, Ikuta T, Peters BD, Malhotra AK, Szeszko PR.	Neuropsychopharmacology	2016
**371**	Concurrent white and gray matter degeneration of disease-specific networks in early-stage Alzheimer's disease and behavioral variant frontotemporal dementia	Steketee RM, Meijboom R, de Groot M, Bron EE, Niessen WJ, van der Lugt A, van Swieten JC, Smits M.	Neurobiol Aging	2016
**372**	The Association Between Retinal Neuronal Layer and Brain Structure is Disrupted in Patients with Cognitive Impairment and Alzheimer's Disease	Liu S, Ong YT, Hilal S, Loke YM, Wong TY, Chen CL, Cheung CY, Zhou J.	J Alzheimers Dis	2016
**373**	Cerebral White Matter and Slow Gait: Contribution of Hyperintensities and	Rosario BL, Rosso AL, Aizenstein HJ, Harris T, Newman AB, Satterfield S, Studenski SA, Yaffe K, Rosano C; Health ABC Study.	J Gerontol A Biol Sci Med Sci	2016

	Normal-appearing Parenchyma			
**374**	Structural white matter differences underlying heterogeneous learning abilities after TBI	Chiou KS, Genova HM, Chiaravalloti ND.	Brain Imaging Behav	2016
**375**	Distinct white-matter aberrations in 22q11.2 deletion syndrome and patients at ultra-high risk for psychosis	Bakker G, Caan MW, Schluter RS, Bloemen OJ, da Silva-Alves F, de Koning MB, Boot E, Vingerhoets WA, Nieman DH, de Haan L, Booij J, van Amelsvoort TA.	Psychol Med	2016
**376**	Abnormal white matter microstructure in drug-naive first episode schizophrenia patients before and after eight weeks of antipsychotic treatment	Zeng B, Ardekani BA, Tang Y, Zhang T, Zhao S, Cui H, Fan X, Zhuo K, Li C, Xu Y, Goff DC, Wang J.	Schizophr Res	2016
**377**	Shared white matter alterations across emotional disorders: A voxel-based meta-analysis of fractional anisotropy	Jenkins LM, Barba A, Campbell M, Lamar M, Shankman SA, Leow AD, Ajilore O, Langenecker SA.	Neuroimage Clin	2016
**378**	Evaluation of white matter integrity in systemic lupus erythematosus by diffusion tensor magnetic resonance imaging: a study using tract-based spatial statistics	Corrêa DG, Zimmermann N, Pereira DB, Doring TM, Netto TM, Ventura N, Fonseca RP, Gasparetto EL.	Neuroradiology	2016
**379**	Altered tract-specific white matter microstructure is related to poorer cognitive performance: The Rotterdam Study	Cremers LG, de Groot M, Hofman A, Krestin GP, van der Lugt A, Niessen WJ, Vernooij MW, Ikram MA.	Neurobiol Aging	2016
**380**	Alterations in white matter volume and integrity in obesity and type 2 diabetes	van Bloemendaal L, Ijzerman RG, Ten Kulve JS, Barkhof F, Diamant M, Veltman DJ, van Duinkerken E.	Metab Brain Dis	2016
**381**	Nε-(carboxymethyl)-lysine, White Matter, and Cognitive Function in Diabetes Patients	Zhang JH, Xu HZ, Shen QF, Lin YZ, Sun CK, Sha L, Ge YS, Liu Y, Wang C.	Can J Neurol Sci	2016
**382**	Probabilistic maps of the white matter tracts with known associated functions on the neonatal brain atlas: Application to evaluate longitudinal developmental trajectories in term-born and preterm-born infants	Akazawa K, Chang L, Yamakawa R, Hayama S, Buchthal S, Alicata D, Andres T, Castillo D, Oishi K, Skranes J, Ernst T, Oishi K.	Neuroimage	2016
**383**	Longitudinal changes in microstructural white matter metrics in Alzheimer's disease	Mayo CD, Mazerolle EL, Ritchie L, Fisk JD, Gawryluk JR; Alzheimer's Disease Neuroimaging Initiative.	Neuroimage Clin	2016

**384**	Differing Connectivity of Exner's Area for Numbers and Letters	Klein E, Willmes K, Jung S, Huber S, Braga LW, Moeller K.	Front Hum Neurosci	2016
**385**	Cardiorespiratory fitness and white matter integrity in Alzheimer's disease	Perea RD, Vidoni ED, Morris JK, Graves RS, Burns JM, Honea RA.	Brain Imaging Behav	2016
**386**	White matter microstructure in a genetically defined group at increased risk of autism symptoms, and a comparison with idiopathic autism: an exploratory study	Goddard MN, van Rijn S, Rombouts SA, Swaab H.	Brain Imaging Behav	2016
**387**	Loss of Consciousness Is Related to White Matter Injury in Mild Traumatic Brain Injury	Wilde EA, Li X, Hunter JV, Narayana PA, Hasan K, Biekman B, Swank P, Robertson C, Miller E, McCauley SR, Chu ZD, Faber J, McCarthy J, Levin HS.	J Neurotrauma	2016
**388**	Diffusion-tensor imaging of major white matter tracts and their role in language processing in aphasia	Ivanova MV, Isaev DY, Dragoy OV, Akinina YS, Petrushevskiy AG, Fedina ON, Shklovsky VM, Dronkers NF.	Cortex	2016
**389**	Discrepancy between perceived pain and cortical processing: A voxel-based morphometry and contact heat evoked potential study	Kramer JLK, Jutzeler CR, Haefeli J, Curt A, Freund P.	Clin Neurophysiol	2016
**390**	Fiber tracts of the dorsal language stream in the human brain	Yagmurlu K, Middlebrooks EH, Tanriover N, Rhoton AL Jr.	J Neurosurg	2016
**391**	Identifying preoperative language tracts and predicting postoperative functional recovery using HARDI q-ball fiber tractography in patients with gliomas	Caverzasi E, Hervey-Jumper SL, Jordan KM, Lobach IV, Li J, Panara V, Racine CA, Sankaranarayanan V, Amirbekian B, Papinutto N, Berger MS, Henry RG.	J Neurosurg	2016
**392**	Left Spatial Neglect Evoked by Electrostimulation of the Right Inferior Fronto-occipital Fasciculus	Herbet G, Yordanova YN, Duffau H.	Brain Topogr	2017
**393**	A diffusion spectrum imaging-based tractographic study into the anatomical subdivision and cortical connectivity of the ventral external capsule: uncinate and inferior fronto-occipital fascicles	Panesar SS, Yeh FC, Deibert CP, Fernandes-Cabral D, Rowthu V, Celtikci P, Celtikci E, Hula WD, Pathak S, Fernández-Miranda JC.	Neuroradiology	2017
**394**	Pathways of the inferior frontal occipital fasciculus in overt speech and reading	Rollans C, Cheema K, Georgiou GK, Cummine J.	Neuroscience	2017
**395**	Direct evidence for the contributive role of the right inferior	Herbet G, Moritz-Gasser S, Duffau H.	Brain Struct Funct	2017

	fronto-occipital fasciculus in non-verbal semantic cognition			
**396**	Neural pathways subserving face-based mentalizing	Yordanova YN, Duffau H, Herbet G.	Brain Struct Funct	2017
**397**	Strength of Temporal White Matter Pathways Predicts Semantic Learning	Ripollés P, Biel D, Peñaloza C, Kaufmann J, Marco-Pallarés J, Noesselt T, Rodríguez-Fornells A.	J Neurosci	2017
**398**	Occipital White Matter Tracts in Human and Macaque	Takemura H, Pestilli F, Weiner KS, Keliris GA, Landi SM, Sliwa J, Ye FQ, Barnett MA, Leopold DA, Freiwald WA, Logothetis NK, Wandell BA.	Cereb Cortex	2017
**399**	Machine-learning classification of 22q11.2 deletion syndrome: A diffusion tensor imaging study	Tylee DS, Kikinis Z, Quinn TP, Antshel KM, Fremont W, Tahir MA, Zhu A, Gong X, Glatt SJ, Coman IL, Shenton ME, Kates WR, Makris N.	Neuroimage Clin	2017
**400**	Bilingualism modulates the white matter structure of language-related pathways	Hämäläinen S, Sairanen V, Leminen A, Lehtonen M.	Neuroimage	2017
**401**	Clinical and Imaging Evaluation of Transuncus Selective Amygdalohippocampectomy	Ghizoni E, Matias RN, Lieber S, de Campos BM, Yasuda CL, de Souza JPSAS, Pereira PC, Amato Filho ACS, Joaquim AF, Lopes TM, Tedeschi H, Cendes F.	World Neurosurg	2017
**402**	White matter abnormalities in long-term anabolic-androgenic steroid users: A pilot study	Seitz J, Lyall AE, Kanayama G, Makris N, Hudson JI, Kubicki M, Pope HG Jr, Kaufman MJ.	Psychiatry Res Neuroimaging	2017
**403**	Tracting the neural basis of music: Deficient structural connectivity underlying acquired amusia	Sihvonen AJ, Ripollés P, Särkämö T, Leo V, Rodríguez-Fornells A, Saunavaara J, Parkkola R, Soinila S.	Cortex	2017
**404**	Early detection of neonatal hypoxic-ischemic white matter injury: an MR diffusion tensor imaging study	Seo Y, Kim GT, Choi JW.	Neuroreport	2017
**405**	The social brain network in 22q11.2 deletion syndrome: a diffusion tensor imaging study	Olszewski AK, Kikinis Z, Gonzalez CS, Coman IL, Makris N, Gong X, Rathi Y, Zhu A, Antshel KM, Fremont W, Kubicki MR, Bouix S, Shenton ME, Kates WR.	Behav Brain Funct	2017
**406**	State-dependent microstructural white matter changes in drug-naïve patients with first-episode psychosis	Serpa MH, Doshi J, Erus G, Chaim-Avancini TM, Cavallet M, van de Bilt MT, Sallet PC, Gattaz WF, Davatzikos C, Busatto GF, Zanetti MV.	Psychol Med	2017
**407**	Toward a functional neuroanatomy of semantic aphasia: A history and ten new cases	Dragoy O, Akinina Y, Dronkers N.	Cortex	2017
**408**	DTI measures identify mild and moderate TBI cases among patients with complex health problems: A receiver operating characteristic analysis of U.S. veterans	Main KL, Soman S, Pestilli F, Furst A, Noda A, Hernandez B, Kong J, Cheng J, Fairchild JK, Taylor J, Yesavage J, Wesson Ashford J, Kraemer H, Adamson MM.	Neuroimage Clin	2017

**409**	Beyond the Arcuate Fasciculus: Damage to Ventral and Dorsal Language Pathways in Aphasia	Yang M, Li Y, Li J, Yao D, Liao W, Chen H.	Brain Topogr	2017
**410**	White Matter Abnormalities in Children with HIV Infection and Exposure	Jankiewicz M, Holmes MJ, Taylor PA, Cotton MF, Laughton B, van der Kouwe AJW, Meintjes EM.	Front Neuroanat	2017
**411**	Abnormalities of brain neural circuits related to obesity: A Diffusion Tensor Imaging study	Papageorgiou I, Astrakas LG, Xydis V, Alexiou GA, Bargiotas P, Tzarouchi L, Zikou AK, Kiortsis DN, Argyropoulou MI.	Magn Reson Imaging	2017
**412**	Long association tracts of the human white matter: an analysis of 18 hemisphere dissections and in vivo HARDI-CSD tractography	Goryainov SA, Kondrashov AV, Gol'dberg MF, Batalov AI, Sufianov RA, Zakharova NE, Pronin IN, Gol'bin DA, Zhukov VY, Dobrovol'sky GF, Shelyakin SY, Vorob'ev VN, Dadykin SS, Potapov AA.	Zh Vopr Neirokhir Im N N Burdenko	2017
**413**	Is Congenital Amusia a Disconnection Syndrome? A Study Combining Tract- and Network-Based Analysis	Wang J, Zhang C, Wan S, Peng G.	Front Hum Neurosci	2017
**414**	Tract-specific white matter hyperintensities disrupt neural network function in Alzheimer's disease	Taylor ANW, Kambeitz-Ilankovic L, Gesierich B, Simon-Vermot L, Franzmeier N, Araque Caballero MÁ, Müller S, Hesheng L, Ertl-Wagner B, Bürger K, Weiner MW, Dichgans M, Duering M, Ewers M; Alzheimer's Disease Neuroimaging Initiative (ADNI).	Alzheimers Dement	2017
**415**	Design fluency and neuroanatomical correlates in 54 neurosurgical patients with lesions to the right hemisphere	Marin D, Madotto E, Fabbro F, Skrap M, Tomasino B.	J Neurooncol	2017
**416**	Age-related changes of white matter association tracts in normal children throughout adulthood: a diffusion tensor tractography study	Mohammad SA, Nashaat NH.	Neuroradiology	2017
**417**	Structural connectivity subserving verbal fluency revealed by lesion-behavior mapping in stroke patients	Li M, Zhang Y, Song L, Huang R, Ding J, Fang Y, Xu Y, Han Z.	Neuropsychologia	2017
**418**	Changes in White-Matter Connectivity in Late Second Language Learners: Evidence from Diffusion Tensor Imaging	Rossi E, Cheng H, Kroll JF, Diaz MT, Newman SD.	Front Psychol	2017
**419**	Impact of Gradient Number and Voxel Size on Diffusion Tensor Imaging Tractography for Resective Brain Surgery	Hoefnagels FWA, de Witt Hamer PC, Pouwels PJW, Barkhof F, Vandertop WP.	World Neurosurg	2017
**420**	White matter microstructure	Nikki Arrington C, Kulesz PA, Juranek J, Cirino PT, Fletcher JM.	Brain Lang	2017

	integrity in relation to reading proficiency☆			
**421**	Differential Signatures of Second Language Syntactic Performance and Age on the Structural Properties of the Left Dorsal Pathway	Yamamoto K, Sakai KL.	Front Psychol	2017
**422**	Reduced white matter integrity and facial emotion perception in never-medicated patients with first-episode schizophrenia: A diffusion tensor imaging study	Zhao X, Sui Y, Yao J, Lv Y, Zhang X, Jin Z, Chen L, Zhang X.	Prog Neuropsychopharmacol Biol Psychiatry	2017
**423**	Three-Dimensional Anatomy of the White Matter Fibers of the Temporal Lobe: Surgical Implications	Pescatori L, Tropeano MP, Manfreda A, Delfini R, Santoro A.	World Neurosurg	2017
**424**	Estimating the Heritability of Structural and Functional Brain Connectivity in Families Affected by Attention-Deficit/Hyperactivity Disorder	Sudre G, Choudhuri S, Szekely E, Bonner T, Goduni E, Sharp W, Shaw P.	JAMA Psychiatry	2017
**425**	A Simplified Method of Accurate Postprocessing of Diffusion Tensor Imaging for Use in Brain Tumor Resection	Bonney PA, Conner AK, Boettcher LB, Cheema AA, Glenn CA, Smitherman AD, Pittman NA, Sughrue ME.	Oper Neurosurg (Hagerstown)	2017
**426**	Comparison of two different analysis approaches for DTI free-water corrected and uncorrected maps in the study of white matter microstructural integrity in individuals with depression	Bergamino M, Kuplicki R, Victor TA, Cha YH, Paulus MP.	Hum Brain Mapp	2017
**427**	Medial prefrontal cortex deficits correlate with unrefreshing sleep in patients with chronic fatigue syndrome	Shan ZY, Kwiatek R, Burnet R, Del Fante P, Staines DR, Marshall-Gradisnik SM, Barnden LR.	NMR Biomed	2017
**428**	Visualization of subcortical language pathways by diffusion tensor imaging fiber tracking based on rTMS language mapping	Negwer C, Ille S, Hauck T, Sollmann N, Maurer S, Kirschke JS, Ringel F, Meyer B, Krieg SM.	Brain Imaging Behav	2017
**429**	Cognitive Variability during Middle-Age: Possible Association with Neurodegeneration and Cognitive Reserve	Ferreira D, Machado A, Molina Y, Nieto A, Correia R, Westman E, Barroso J.	Front Aging Neurosci	2017

**430**	White Matter Correlates of Auditory Comprehension Outcomes in Chronic Post-Stroke Aphasia	Xing S, Lacey EH, Skipper-Kallal LM, Zeng J, Turkeltaub PE.	Front Neurol	2017
**431**	White matter microstructure and volitional motor activity in schizophrenia: A diffusion kurtosis imaging study	Docx L, Emsell L, Van Hecke W, De Bondt T, Parizel PM, Sabbe B, Morrens M.	Psychiatry Res Neuroimaging	2017
**432**	"Can touch this": Cross-modal shape categorization performance is associated with microstructural characteristics of white matter association pathways	Lee Masson H, Wallraven C, Petit L.	Hum Brain Mapp	2017
**433**	White Matter Abnormalities Associated With Subsyndromal Psychotic-Like Symptoms Predict Later Social Competence in Children and Adolescents	DeRosse P, Ikuta T, Karlsgodt KH, Peters BD, Gopin CB, Szeszko PR, Malhotra AK.	Schizophr Bull	2017
**434**	Diffusion tensor imaging of normal-appearing white matter in patients with neuromyelitis optica spectrum disorder and multiple sclerosis	Kim SH, Kwak K, Hyun JW, Joung A, Lee SH, Choi YH, Lee JM, Kim HJ.	Eur J Neurol	2017
**435**	Structural changes in Parkinson's disease: voxel-based morphometry and diffusion tensor imaging analyses based on (123)I-MIBG uptake	Kikuchi K, Hiwatashi A, Togao O, Yamashita K, Somehara R, Kamei R, Baba S, Yamaguchi H, Kira JI, Honda H.	Eur Radiol	2017
**436**	A longitudinal study of changes in Diffusion Tensor Value and their association with cognitive sequelae among patients with mild head injury	Munivenkatappa A, Bhagavatula ID, Shukla DP, Rajeswaran J.	J Neurosurg Sci	2017
**437**	Abnormal white matter microstructure among early adulthood smokers: a tract-based spatial statistics study	Wang S, Zuo L, Jiang T, Peng P, Chu S, Xiao D.	Neurol Res	2017
**438**	White Matter Tract Integrity in Alzheimer's Disease vs. Late Onset Bipolar Disorder and Its Correlation with Systemic Inflammation and Oxidative Stress Biomarkers	Besga A, Chyzhyk D, Gonzalez-Ortega I, Echeveste J, Graña-Lecuona M, Graña M, Gonzalez-Pinto A.	Front Aging Neurosci	2017

**439**	Neuronavigated Fiber Dissection with Pial Preservation: Laboratory Model to Simulate Opercular Approaches to Insular Tumors	Mandonnet E, Martino J, Sarubbo S, Corrivetti F, Bouazza S, Bresson D, Duffau H, Froelich S.	World Neurosurg	2017
**440**	Neuroanatomical correlates of stroke-associated infection and stroke-induced immunodepression	Urra X, Laredo C, Zhao Y, Amaro S, Rudilosso S, Renú A, Prats-Galino A, Planas AM, Oleaga L, Chamorro Á.	Brain Behav Immun	2017
**441**	Disrupted White Matter Microstructure and Mood Disorders after Traumatic Brain Injury	Spitz G, Alway Y, Gould KR, Ponsford JL.	J Neurotrauma	2017
**442**	Fibre-specific white matter changes in multiple sclerosis patients with optic neuritis	Gajamange S, Raffelt D, Dhollander T, Lui E, van der Walt A, Kilpatrick T, Fielding J, Connelly A, Kolbe S.	Neuroimage Clin	2017
**443**	Voluntary saccade inhibition deficits correlate with extended white-matter cortico-basal atrophy in Huntington's disease	Vaca-Palomares I, Coe BC, Brien DC, Campos-Romo A, Munoz DP, Fernandez-Ruiz J.	Neuroimage Clin	2017
**444**	Brain Arteriovenous Malformations Located in Language Area: Surgical Outcomes and Risk Factors for Postoperative Language Deficits	Jiao Y, Lin F, Wu J, Li H, Chen X, Li Z, Ma J, Cao Y, Wang S, Zhao J.	World Neurosurg	2017
**445**	Sex differences in associations between white matter microstructure and gonadal hormones in children and adolescents with prenatal alcohol exposure	Uban KA, Herting MM, Wozniak JR, Sowell ER; CIFASD.	Psychoneuroendocrinology	2017
**446**	Disconnection as a mechanism for social cognition impairment in multiple sclerosis	Batista S, Alves C, d'Almeida OC, Afonso A, Félix-Morais R, Pereira J, Macário C, Sousa L, Castelo-Branco M, Santana I, Cunha L.	Neurology	2017
**447**	Statistical differences in the white matter tracts in subjects with depression by using different skeletonized voxel-wise analysis approaches and DTI fitting procedures	Bergamino M, Farmer M, Yeh HW, Paul E, Hamilton JP.	Brain Res	2017
**448**	Cross-Modal Recruitment of Auditory and Orofacial Areas During Sign Language in a Deaf Subject	Martino J, Velasquez C, Vázquez-Bourgon J, de Lucas EM, Gomez E.	World Neurosurg	2017
**449**	Night sleep influences white matter microstructure in bipolar depression	Benedetti F, Melloni EMT, Dallaspezia S, Bollettini I, Locatelli C, Poletti S, Colombo C.	J Affect Disord	2017

**450**	Microstructural abnormalities in white and gray matter in obese adolescents with and without type 2 diabetes	Nouwen A, Chambers A, Chechlacz M, Higgs S, Blissett J, Barrett TG, Allen HA.	Neuroimage Clin	2017
**451**	Changes to white matter microstructure in transient ischemic attack: A longitudinal diffusion tensor imaging study	Ferris JK, Edwards JD, Ma JA, Boyd LA.	Hum Brain Mapp	2017
**452**	Identification and individualized prediction of clinical phenotypes in bipolar disorders using neurocognitive data, neuroimaging scans and machine learning	Wu MJ, Mwangi B, Bauer IE, Passos IC, Sanches M, Zunta-Soares GB, Meyer TD, Hasan KM, Soares JC.	Neuroimage	2017
**453**	Disrupted white matter structural networks in healthy older adult APOE ε4 carriers - An international multicenter DTI study	Cavedo E, Lista S, Rojkova K, Chiesa PA, Houot M, Brueggen K, Blautzik J, Bokde ALW, Dubois B, Barkhof F, Pouwels PJW, Teipel S, Hampel H; Alzheimer Precision Medicine Initiative (APMI).	Neuroscience	2017
**454**	Revisiting the human uncinate fasciculus, its subcomponents and asymmetries with stem-based tractography and microdissection validation	Hau J, Sarubbo S, Houde JC, Corsini F, Girard G, Deledalle C, Crivello F, Zago L, Mellet E, Jobard G, Joliot M, Mazoyer B, Tzourio-Mazoyer N, Descoteaux M, Petit L.	Brain Struct Funct	2017
**455**	Early dynamics of white matter deficits in children developing dyslexia	Vanderauwera J, Wouters J, Vandermosten M, Ghesquière P.	Dev Cogn Neurosci	2017
**456**	Brain pathway differences between Parkinson's disease patients with and without REM sleep behavior disorder	Ansari M, Rahmani F, Dolatshahi M, Pooyan A, Aarabi MH.	Sleep Breath	2017
**457**	Microstructural white matter tract alteration in Prader-Willi syndrome: A diffusion tensor imaging study	Rice LJ, Lagopoulos J, Brammer M, Einfeld SL.	Am J Med Genet C Semin Med Genet	2017
**458**	Analysis of an ATP-induced conformational transition of ABC transporter MsbA using a coarse-grained model	Arai N, Furuta T, Sakurai M.	Biophys Physicobiol	2017
**459**	Exploring conformational equilibria of a heterodimeric ABC transporter	Timachi MH, Hutter CA, Hohl M, Assafa T, Böhm S, Mittal A, Seeger MA, Bordignon E.	Elife	2017
**460**	Sequential language learning and language immersion in bilingualism: diffusion	Rahmani F, Sobhani S, Aarabi MH.	Exp Brain Res	2017

	MRI connectometry reveals microstructural evidence			
**461**	The white matter tracts of the cerebrum in ventricular surgery and hydrocephalus	Güngör A, Baydin S, Middlebrooks EH, Tanriover N, Isler C, Rhoton AL Jr.	J Neurosurg	2017
**462**	Language outcomes after resection of dominant inferior parietal lobule gliomas	Southwell DG, Riva M, Jordan K, Caverzasi E, Li J, Perry DW, Henry RG, Berger MS.	J Neurosurg	2017
**463**	Microsurgical anatomy of the central core of the brain	Ribas EC, Yağmurlu K, de Oliveira E, Ribas GC, Rhoton A.	J Neurosurg	2018
**464**	Inter-individual variations and hemispheric asymmetries in structural connectivity patterns of the inferior fronto-occipital fascicle: a diffusion tensor imaging tractography study	Vassal F, Pommier B, Sontheimer A, Lemaire JJ.	Surg Radiol Anat	2018
**465**	A Connectomic Atlas of the Human Cerebrum-Chapter 13: Tractographic Description of the Inferior Fronto-Occipital Fasciculus	Conner AK, Briggs RG, Sali G, Rahimi M, Baker CM, Burks JD, Glenn CA, Battiste JD, Sughrue ME.	Oper Neurosurg (Hagerstown)	2018
**466**	Anterior Temporal Lobe Morphometry Predicts Categorization Ability	Garcin B, Urbanski M, Thiebaut de Schotten M, Levy R, Volle E.	Front Hum Neurosci	2018
**467**	The Relationship between White Matter and Reading Acquisition, Refinement and Maintenance	Cheema K, Cummine J;<br>for the Pediatric Imaging, Neurocognition, and Ge netics Study.	Dev Neurosci	2018
**468**	Relationship between white matter integrity and serum inflammatory cytokine levels in drug-naive patients with major depressive disorder: diffusion tensor imaging study using tract-based spatial statistics	Sugimoto K, Kakeda S, Watanabe K, Katsuki A, Ueda I, Igata N, Igata R, Abe O, Yoshimura R, Korogi Y.	Transl Psychiatry	2018
**469**	The allocentric neglect due to injury of the inferior fronto-occipital fasciculus in a stroke patient: A case report	Jang SH, Jang WH.	Medicine (Baltimore)	2018
**470**	Structure tensor informed fibre tractography at 3T	Chan KS, Norris DG, Marques JP.	Hum Brain Mapp	2018
**471**	Structural connectivity in spatial attention network: reconstruction from left hemispatial neglect	Hattori T, Ito K, Nakazawa C, Numasawa Y, Watanabe M, Aoki S, Mizusawa H, Ishiai S, Yokota T.	Brain Imaging Behav	2018

**472**	Anatomy and white matter connections of the orbitofrontal gyrus	Burks JD, Conner AK, Bonney PA, Glenn CA, Baker CM, Boettcher LB, Briggs RG, O'Donoghue DL, Wu DH, Sughrue ME.	J Neurosurg	2018
**473**	The auditory cortex network in the posterior superior temporal area	Suzuki Y, Enatsu R, Kanno A, Ochi S, Mikuni N.	Clin Neurophysiol	2018
**474**	Altered White-Matter Microstructure in Conduct Disorder Is Specifically Associated with Elevated Callous-Unemotional Traits	Puzzo I, Seunarine K, Sully K, Darekar A, Clark C, Sonuga-Barke EJS, Fairchild G.	J Abnorm Child Psychol	2018
**475**	Common brain networks for distinct deficits in visual neglect. A combined structural and tractography MRI approach	Toba MN, Migliaccio R, Batrancourt B, Bourlon C, Duret C, Pradat-Diehl P, Dubois B, Bartolomeo P.	Neuropsychologia	2018
**476**	Formal thought disorder is related to aberrations in language-related white matter tracts in patients with schizophrenia	Cavelti M, Winkelbeiner S, Federspiel A, Walther S, Stegmayer K, Giezendanner S, Laimböck K, Dierks T, Strik W, Horn H, Homan P.	Psychiatry Res Neuroimaging	2018
**477**	Analysis of alterations in white matter integrity of adult patients with comitant exotropia	Li D, Li S, Zeng X.	J Int Med Res	2018
**478**	Advanced diffusion imaging for assessing normal white matter development in neonates and characterizing aberrant development in congenital heart disease	Karmacharya S, Gagoski B, Ning L, Vyas R, Cheng HH, Soul J, Newberger JW, Shenton ME, Rathi Y, Grant PE.	Neuroimage Clin	2018
**479**	Alterations in white matter pathways underlying phonological and morphological processing in Chinese developmental dyslexia	Su M, Zhao J, Thiebaut de Schotten M, Zhou W, Gong G, Ramus F, Shu H.	Dev Cogn Neurosci	2018
**480**	Analysis of white matter characteristics with tract-based spatial statistics according to diffusion tensor imaging in early Parkinson's disease	Li XR, Ren YD, Cao B, Huang XL.	Neurosci Lett	2018
**481**	Diffusion Tensor Imaging With Tract-Based Spatial Statistics Reveals White Matter Abnormalities in Patients With Vascular Cognitive Impairment	Chen HJ, Gao YQ, Che CH, Lin H, Ruan XL.	Front Neuroanat	2018
**482**	Longitudinal structural gray matter and white matter MRI changes in presymptomatic	Olm CA, McMillan CT, Irwin DJ, Van Deerlin VM, Cook PA, Gee JC, Grossman M.	Neuroimage Clin	2018

	progranulin mutation carriers			
**483**	Impaired White Matter Integrity and Social Cognition in High-Function Autism: Diffusion Tensor Imaging Study	Im WY, Ha JH, Kim EJ, Cheon KA, Cho J, Song DH.	Psychiatry Investig	2018
**484**	Neural organization of ventral white matter tracts parallels the initial steps of reading development: A DTI tractography study	Vanderauwera J, De Vos A, Forkel SJ, Catani M, Wouters J, Vandermosten M, Ghesquière P.	Brain Lang	2018
**485**	Plasticity in deep and superficial white matter: a DTI study in world class gymnasts	Deng F, Zhao L, Liu C, Lu M, Zhang S, Huang H, Chen L, Wu X, Niu C, He Y, Wang J, Huang R.	Brain Struct Funct	2018
**486**	Development of white matter microstructure in relation to verbal and visuospatial working memory-A longitudinal study	Krogsrud SK, Fjell AM, Tamnes CK, Grydeland H, Due-Tønnessen P, Bjørnerud A, Sampaio-Baptista C, Andersson J, Johansen-Berg H, Walhovd KB.	PLoS One	2018
**487**	Multifunctional Roles of the Ventral Stream in Language Models: Advanced Segmental Quantification in Post-Stroke Aphasic Patients	Zhang J, Wei X, Xie S, Zhou Z, Shang D, Ji R, Yu Y, He F, Du Y, Ye X, Luo B.	Front Neurol	2018
**488**	Language-Eloquent White Matter Pathway Tractography and the Course of Language Function in Glioma Patients	Ille S, Engel L, Kelm A, Meyer B, Krieg SM.	Front Oncol	2018
**489**	Distributed performance of white matter properties in chess players: A DWI study using automated fiber quantification	Zhou S, Jin L, He J, Zeng Q, Wu Y, Cao Z, Feng Y.	Brain Res	2018
**490**	Awake Surgery for Gliomas within the Right Inferior Parietal Lobule: New Insights into the Functional Connectivity Gained from Stimulation Mapping and Surgical Implications	Rolland A, Herbet G, Duffau H.	World Neurosurg	2018
**491**	The Influence of Myelin Oligodendrocyte Glycoprotein on White Matter Abnormalities in Different Onset Age of Drug-Naïve Depression	Wu F, Kong L, Zhu Y, Zhou Q, Jiang X, Chang M, Zhou Y, Cao Y, Xu K, Wang F, Tang Y.	Front Psychiatry	2018
**492**	Endoscopic Approach of the Insula Through the Anterior Middle Temporal Gyrus: A Feasibility Study in the Laboratory	Corrivetti F, Froelich S, Mandonnet E.	Oper Neurosurg (Hagerstown)	2018

**493**	Diffusion tensor imaging and tractography of the white matter in normal aging: The rate-of-change differs between segments within tracts	Mårtensson J, Lätt J, Åhs F, Fredrikson M, Söderlund H, Schiöth HB, Kok J, Kremer B, van Westen D, Larsson EM, Nilsson M.	Magn Reson Imaging	2018
**494**	Cerebral White Matter Changes in Young Healthy Individuals With High Trait Anxiety: A Tract-Based Spatial Statistics Study	Lu M, Yang C, Chu T, Wu S.	Front Neurol	2018
**495**	Neuropsychological evidence for the crucial role of the right arcuate fasciculus in the face-based mentalizing network: A disconnection analysis	Nakajima R, Yordanova YN, Duffau H, Herbet G.	Neuropsychologia	2018
**496**	Awake resection of a left operculo-insular low-grade glioma guided by cortico-subcortical mapping	Picart T, Duffau H.	Neurosurg Focus	2018
**497**	The different association of allocentric and egocentric neglect with dorsal and ventral pathways: A case report	Jang SH, Jang WH.	Medicine (Baltimore)	2018
**498**	Juvenile Myoclonic Epilepsy Shows Potential Structural White Matter Abnormalities: A TBSS Study	Domin M, Bartels S, Geithner J, Wang ZI, Runge U, Grothe M, Langner S, von Podewils F.	Front Neurol	2018
**499**	Structural Connections of Functionally Defined Human Insular Subdivisions	Nomi JS, Schettini E, Broce I, Dick AS, Uddin LQ.	Cereb Cortex	2018
**500**	Impaired long contact white matter fibers integrity is related to depression in Parkinson's disease	Wu JY, Zhang Y, Wu WB, Hu G, Xu Y.	CNS Neurosci Ther	2018
**501**	Symmetric tract-based spatial statistics of patients with left versus right mesial temporal lobe epilepsy with hippocampal sclerosis	Mennecke AB, Rösch J, Kasper BS, Schmidt MA, Laun FB, Rössler K, Coras R, Hamer HM, Dörfler A.	Neuroreport	2018
**502**	Physical neglect during childhood alters white matter connectivity in healthy young males	Tendolkar I, Mårtensson J, Kühn S, Klumpers F, Fernández G.	Hum Brain Mapp	2018
**503**	Diffusion-weighted MRI measures suggest increased white-matter integrity in Internet gaming disorder: Evidence from the comparison with	Dong G, Wu L, Wang Z, Wang Y, Du X, Potenza MN.	Addict Behav	2018

	recreational Internet game users			
**504**	Neuroanatomical variations as a function of experience in a complex daily task: A VBM and DTI study on driving experience	Megías A, Petrova D, Navas JF, Cándido A, Maldonado A, Catena A.	Brain Imaging Behav	2018
**505**	Latent class analysis of attention and white matter correlation in children with attention-deficit/hyperactivity disorder	Rossi ASU, Moura LM, Miranda MC, Muszkat M, Mello CB, Bueno OFA.	Braz J Med Biol Res	2018
**506**	Multisensory integration processing during olfactory-visual stimulation-An fMRI graph theoretical network analysis	Ripp I, Zur Nieden AN, Blankenagel S, Franzmeier N, Lundström JN, Freiherr J.	Hum Brain Mapp	2018
**507**	Elevated body-mass index is associated with reduced white matter integrity in two large independent cohorts	Repple J, Opel N, Meinert S, Redlich R, Hahn T, Winter NR, Kaehler C, Emden D, Leenings R, Grotegerd D, Zaremba D, Bürger C, Förster K, Dohm K, Enneking V, Leehr EJ, Böhnlein J, Karliczek G, Heindel W, Kugel H, Bauer J, Arolt V, Dannlowski U.	Psychoneuroendocrinology	2018
**508**	Preliminary evidence from a prospective DTI study suggests a posterior-to-anterior pattern of recovery in college athletes with sports-related concussion	Cubon VA, Murugavel M, Holmes KW, Dettwiler A.	Brain Behav	2018
**509**	White-matter integrity in patients with systemic lupus erythematosus and memory deficits	Corrêa DG, Zimmermann N, Borges RS, Pereira DB, Doring TM, Tukamoto G, Fonseca RP, Gasparetto EL.	Neuroradiol J	2018
**510**	Reduced white matter connectivity associated with auditory verbal hallucinations in first-episode and chronic schizophrenia: A diffusion tensor imaging study	Zhang X, Gao J, Zhu F, Wang W, Fan Y, Ma Q, Ma X, Yang J.	Psychiatry Res Neuroimaging	2018
**511**	Left Transsylvian Transcisternal and Transinferior Insular Sulcus Approach for Resection of Uncohippocampal Tumor: 3-Dimensional Operative Video	Fernandez-Miranda JC.	Oper Neurosurg (Hagerstown)	2018
**512**	White matter aberrations and age-related trajectories in patients with schizophrenia and bipolar disorder revealed by diffusion tensor imaging	Tønnesen S, Kaufmann T, Doan NT, Alnæs D, Córdova-Palomera A, Meer DV, Rokicki J, Moberget T, Gurholt TP, Haukvik UK, Ueland T, Lagerberg TV, Agartz I, Andreassen OA, Westlye LT.	Sci Rep	2018

**513**	White matter measures correlate with essential tremor severity-A pilot diffusion tensor imaging study	Nestrasil I, Svatkova A, Rudser KD, Chityala R, Wakumoto A, Mueller BA, Bednařík P, Tuite P, Wu X, Bushara K.	Brain Behav	2018
**514**	Altered white matter development in children born very preterm	Young JM, Vandewouw MM, Morgan BR, Smith ML, Sled JG, Taylor MJ.	Brain Struct Funct	2018
**515**	White matter microstructural alterations in clinically isolated syndrome and multiple sclerosis	Huang J, Liu Y, Zhao T, Shu N, Duan Y, Ren Z, Sun Z, Liu Z, Chen H, Dong H, Li K.	J Clin Neurosci	2018
**516**	Childhood adversity associated with white matter alteration in the corpus callosum, corona radiata, and uncinate fasciculus of psychiatrically healthy adults	McCarthy-Jones S, Oestreich LKL, Lyall AE, Kikinis Z, Newell DT, Savadjiev P, Shenton ME, Kubicki M, Pasternak O, Whitford TJ; Australian Schizophrenia Research Bank.	Brain Imaging Behav	2018
**517**	Longitudinal structural and molecular neuroimaging in agrammatic primary progressive aphasia	Tetzloff KA, Duffy JR, Clark HM, Strand EA, Machulda MM, Schwarz CG, Senjem ML, Reid RI, Spychalla AJ, Tosakulwong N, Lowe VJ, Jack CR Jr, Josephs KA, Whitwell JL.	Brain	2018
**518**	Prefronto-temporal white matter microstructural alterations 20 years after the diagnosis of type 1 diabetes mellitus	Yoon S, Kim J, Musen G, Renshaw PF, Hwang J, Bolo NR, Kim JE, Simonson DC, Weinger K, Ryan CM, Lyoo IK, Jacobson AM.	Pediatr Diabetes	2018
**519**	Gray and white matter changes and their relation to illness trajectory in first episode psychosis	Keymer-Gausset A, Alonso-Solís A, Corripio I, Sauras-Quetcuti RB, Pomarol-Clotet E, Canales-Rodriguez EJ, Grasa-Bello E, Álvarez E, Portella MJ.	Eur Neuropsychopharmacol	2018
**520**	An investigation of white matter integrity and attention deficits following traumatic brain injury	Owens JA, Spitz G, Ponsford JL, Dymowski AR, Willmott C.	Brain Inj	2018
**521**	Critical brain regions related to post-stroke aphasia severity identified by early diffusion imaging are not the same when predicting short- and long-term outcome	Zavanone C, Samson Y, Arbizu C, Dupont S, Dormont D, Rosso C.	Brain Lang	2018
**522**	Brain Structural Correlates of Subclinical Obsessive-Compulsive Symptoms in Healthy Children	Suñol M, Contreras-Rodríguez O, Macià D, Martínez-Vilavella G, Martínez-Zalacaín I, Subirà M, Pujol J, Sunyer J, Soriano-Mas C.	J Am Acad Child Adolesc Psychiatry	2018
**523**	Comprehensive Investigation of White Matter Tracts in Professional Chess Players and Relation to Expertise: Region of Interest and DMRI Connectometry	Mayeli M, Rahmani F, Aarabi MH.	Front Neurosci	2018

**524**	Aerobic Exercise Intervention Alters Executive Function and White Matter Integrity in Deaf Children: A Randomized Controlled Study	Xiong X, Zhu LN, Dong XX, Wang W, Yan J, Chen AG.	Neural Plast	2018
**525**	A purely functional Imaging based approach for transcortical resection of lesion involving the dominant atrium: Towards safer, imaging-guided, tailored cortico-leucotomies	Frati A, Pesce A, D'Andrea G, Fraschetti F, Salvati M, Cimatti M, Esposito V, Raco A.	J Clin Neurosci	2018
**526**	Reduced white matter integrity in borderline personality disorder: A diffusion tensor imaging study	Ninomiya T, Oshita H, Kawano Y, Goto C, Matsuhashi M, Masuda K, Takita F, Izumi T, Inoue A, Higuma H, Kanehisa M, Akiyoshi J.	J Affect Disord	2018
**527**	Changes in language white matter tract microarchitecture associated with cognitive deficits in patients with presumed low-grade glioma	Incekara F, Satoer D, Visch-Brink E, Vincent A, Smits M.	J Neurosurg	2018
**528**	Periinsular anterior quadrantotomy: technical note	Cossu G, Lebon S, Seeck M, Pralong E, Messerer M, Roulet-Perez E, Daniel RT.	J Neurosurg Pediatr	2018
**529**	Method for temporal keyhole lobectomies in resection of low- and high-grade gliomas	Conner AK, Burks JD, Baker CM, Smitherman AD, Pryor DP, Glenn CA, Briggs RG, Bonney PA, Sughrue ME.	J Neurosurg	2018
**530**	Inferior Fronto-Occipital fascicle anatomy in brain tumor surgeries: From anatomy lab to surgical theater	Altieri R, Melcarne A, Junemann C, Zeppa P, Zenga F, Garbossa D, Certo F, Barbagallo G.	J Clin Neurosci	2019
**531**	Uncovering the inferior fronto-occipital fascicle and its topological organization in non-human primates: the missing connection for language evolution	Sarubbo S, Petit L, De Benedictis A, Chioffi F, Ptito M, Dyrby TB.	Brain Struct Funct	2019
**532**	Fiber pathways supporting early literacy development in 5-8-year-old children	Broce IJ, Bernal B, Altman N, Bradley C, Baez N, Cabrera L, Hernandez G, De Feria A, Dick AS.	Brain Cogn	2019
**533**	Microsurgical anatomy of the sagittal stratum	Di Carlo DT, Benedetto N, Duffau H, Cagnazzo F, Weiss A, Castagna M, Cosottini M, Perrini P.	Acta Neurochir (Wien)	2019
**534**	Cognitive correlates of abnormal myelination in psychosis	Vanes LD, Mouchlianitis E, Barry E, Patel K, Wong K, Shergill SS.	Sci Rep	2019
**535**	Associative white matter connecting the dorsal and ventral posterior human cortex	Bullock D, Takemura H, Caiafa CF, Kitchell L, McPherson B, Caron B, Pestilli F.	Brain Struct Funct	2019

**536**	Patient MW: transient visual hemi-agnosia	Decramer T, Premereur E, Lagae L, van Loon J, Janssen P, Sunaert S, Theys T.	J Neurol	2019
**537**	Periventricular White Matter Abnormalities on Diffusion Tensor Imaging of Postural Instability Gait Disorder Parkinsonism	Tan SYZ, Keong NCH, Selvan RMP, Li H, Ooi LQR, Tan EK, Chan LL.	AJNR Am J Neuroradiol	2019
**538**	Anterior peri-insular quadrantotomy: a cadaveric white matter dissection study	Gonzalez-Lopez P, Cossu G, Pralong E, Baldoncini M, Messerer M, Daniel RT.	J Neurosurg Pediatr	2019
**539**	Childhood Trauma Associated White Matter Abnormalities in First-Episode Schizophrenia	Asmal L, Kilian S, du Plessis S, Scheffler F, Chiliza B, Fouche JP, Seedat S, Dazzan P, Emsley R.	Schizophr Bull	2019
**540**	Brain white matter changes in asymptomatic carriers of Leber's hereditary optic neuropathy	Long M, Wang L, Tian Q, Ding H, Qin W, Shi D, Yu C.	J Neurol	2019
**541**	White-matter pathways and semantic processing: intrasurgical and lesion-symptom mapping evidence	Sierpowska J, Gabarrós A, Fernández-Coello A, Camins À, Castañer S, Juncadella M, François C, Rodríguez-Fornells A.	Neuroimage Clin	2019
**542**	Altered White Matter Organization in the TUBB3 E410K Syndrome	Grant PE, Im K, Ahtam B, Laurentys CT, Chan WM, Brainard M, Chew S, Drottar M, Robson CD, Drmic I, Engle EC.	Cereb Cortex	2019
**543**	Inward versus reward: white matter pathways in extraversion	Leshem R, Paoletti P, Piervincenzi C, Carducci F, Mallio CA, Errante Y, Quattrocchi CC, Ben-Soussan TD.	Personal Neurosci	2019
**544**	Consequences of brain tumour resection on emotion recognition	Mattavelli G, Pisoni A, Casarotti A, Comi A, Sera G, Riva M, Bizzi A, Rossi M, Bello L, Papagno C.	J Neuropsychol	2019
**545**	Anatomy and white matter connections of the inferior frontal gyrus	Briggs RG, Chakraborty AR, Anderson CD, Abraham CJ, Palejwala AH, Conner AK, Pelargos PE, O'Donoghue DL, Glenn CA, Sughrue ME.	Clin Anat	2019
**546**	Fronto-limbic white matter microstructure, behavior, and emotion regulation in survivors of pediatric brain tumor	Wier R, Aleksonis HA, Pearson MM, Cannistraci CJ, Anderson AW, Kuttesch JF Jr, Compas BE, Hoskinson KR.	J Neurooncol	2019
**547**	Endoscopic Transanterior Middle Temporal Approach to the Atrium-An Anatomical Feasibility Study	Lau R, Rodriguez Rubio R, Martino J, Sanmillán JL, Benet A, Tayebi Meybodi A, Gandhi S, Kournoutas I, Gabarrós A.	World Neurosurg	2019
**548**	White matter alterations and tract lateralization in children with dyslexia and isolated spelling deficits	Banfi C, Koschutnig K, Moll K, Schulte-Körne G, Fink A, Landerl K.	Hum Brain Mapp	2019
**549**	Alterations in the Structural and Functional Connectivity of the Visuomotor Network of Children With	Bauer CM, Papadelis C.	Semin Pediatr Neurol	2019

	Periventricular Leukomalacia			
**550**	Association Between White Matter Microstructure and Verbal Fluency in Patients With Multiple Sclerosis	Blecher T, Miron S, Schneider GG, Achiron A, Ben-Shachar M.	Front Psychol	2019
**551**	White Matter Language Pathways and Language Performance in Healthy Adults Across Ages	Houston J, Allendorfer J, Nenert R, Goodman AM, Szaflarski JP.	Front Neurosci	2019
**552**	Fronto-temporal vulnerability to disconnection in paediatric moderate and severe traumatic brain injury	Molteni E, Pagani E, Strazzer S, Arrigoni F, Beretta E, Boffa G, Galbiati S, Filippi M, Rocca MA.	Eur J Neurol	2019
**553**	Understanding the link between childhood trauma and schizophrenia: A systematic review of neuroimaging studies	Cancel A, Dallel S, Zine A, El-Hage W, Fakra E.	Neurosci Biobehav Rev	2019
**554**	Mathematical expertise modulates the architecture of dorsal and cortico-thalamic white matter tracts	Jeon HA, Kuhl U, Friederici AD.	Sci Rep	2019
**555**	The interplay of socio-economic status represented by paternal educational level, white matter structure and reading	Vanderauwera J, van Setten ERH, Maurits NM, Maassen BAM.	PLoS One	2019
**556**	Altered structural brain connectivity involving the dorsal and ventral language pathways in 16p11.2 deletion syndrome	Ahtam B, Link N, Hoff E, Ellen Grant P, Im K.	Brain Imaging Behav	2019
**557**	Pre- and Post-therapy Assessment of Clinical Outcomes and White Matter Integrity in Autism Spectrum Disorder: Pilot Study	Saaybi S, AlArab N, Hannoun S, Saade M, Tutunji R, Zeeni C, Shbarou R, Hourani R, Boustany RM.	Front Neurol	2019
**558**	White Matter Plasticity in Reading-Related Pathways Differs in Children Born Preterm and at Term: A Longitudinal Analysis	Bruckert L, Borchers LR, Dodson CK, Marchman VA, Travis KE, Ben-Shachar M, Feldman HM.	Front Hum Neurosci	2019
**559**	Left Pan-Hippocampal Low Grade Glioma-2-Stage Transsylvian Transventricular and Paramedian Supracerebellar Transtentorial Approaches: 2-Dimensional Operative Video	Fernandez-Miranda JC.	Oper Neurosurg (Hagerstown)	2019
**560**	Presurgical simulation for neuroendoscopic	Garcia-Garcia S, Kakaizada S, Oleaga L, Benet A, Rincon-Toroella J, González-Sánchez JJ.	Neurol India	2019

	procedures: Virtual study of the integrity of neurological pathways using diffusion tensor imaging tractography			
**561**	Cognitive and neural mechanisms underlying the mnemonic effect of songs after stroke	Leo V, Sihvonen AJ, Linnavalli T, Tervaniemi M, Laine M, Soinila S, Särkämö T.	Neuroimage Clin	2019
**562**	General psychopathology factor and unresolved-disorganized attachment uniquely correlated to white matter integrity using diffusion tensor imaging	Riem MME, van Hoof MJ, Garrett AS, Rombouts SARB, van der Wee NJA, van IJzendoorn MH, Vermeiren RRJM.	Behav Brain Res	2019
**563**	Longitudinal Changes in Diffusion Tensor Imaging Following Mild Traumatic Brain Injury and Correlation With Outcome	Yin B, Li DD, Huang H, Gu CH, Bai GH, Hu LX, Zhuang JF, Zhang M.	Front Neural Circuits	2019
**564**	Functional and structural connectivity of the brain in very preterm babies: relationship with gestational age and body and brain growth	Mouka V, Drougia A, Xydis VG, Astrakas LG, Zikou AK, Kosta P, Andronikou S, Argyropoulou MI.	Pediatr Radiol	2019
**565**	Dissociating motor-speech from lexico-semantic systems in the left frontal lobe: insight from a series of 17 awake intraoperative mappings in glioma patients	Corrivetti F, de Schotten MT, Poisson I, Froelich S, Descoteaux M, Rheault F, Mandonnet E.	Brain Struct Funct	2019
**566**	Cortical morphometry and structural connectivity relate to executive function and estradiol level in healthy adolescents	Stoica T, Knight LK, Naaz F, Ramic M, Depue BE.	Brain Behav	2019
**567**	Genetic effects on white matter integrity in drug-naive patients with major depressive disorder: a diffusion tensor imaging study of 17 genetic loci associated with depressive symptoms	Kakeda S, Watanabe K, Katsuki A, Sugimoto K, Ueda I, Igata N, Kishi T, Iwata N, Abe O, Yoshimura R, Korogi Y.	Neuropsychiatr Dis Treat	2019
**568**	Longitudinal diffusion weighted imaging of limbic regions in patients with major depressive disorder after 6 years and partial to full remission	Doolin K, Andrews S, Carballedo A, McCarthy H, O'Hanlon E, Tozzi L, Frodl T.	Psychiatry Res Neuroimaging	2019
**569**	Prefrontal-Temporal Pathway Mediates the Cross-Modal and	Luan Y, Wang C, Jiao Y, Tang T, Zhang J, Teng GJ.	Front Neurosci	2019

	Cognitive Reorganization in Sensorineural Hearing Loss With or Without Tinnitus: A Multimodal MRI Study			
**570**	Auditory white matter pathways are associated with effective connectivity of auditory prediction errors within a fronto-temporal network	Oestreich LKL, Randeniya R, Garrido MI.	Neuroimage	2019
**571**	Estimating effects of graded white matter damage and binary tract disconnection on post-stroke language impairment	Geller J, Thye M, Mirman D.	Neuroimage	2019
**572**	Microneuroanatomy of the Anterior Frontal Laser Trajectory to the Insula	Baydin S, Gungor A, Holanda VM, Tanriover N, Danish SF.	World Neurosurg	2019
**573**	White Matter Tract-Cognitive Relationships in Children with High-Functioning Autism Spectrum Disorder	Kato Y, Kagitani-Shimono K, Matsuzaki J, Hanaie R, Yamamoto T, Tominaga K, Watanabe Y, Mohri I, Taniike M.	Psychiatry Investig	2019
**574**	Alterations of structural and functional connectivity in profound sensorineural hearing loss infants within an early sensitive period: A combined DTI and fMRI study	Wang S, Chen B, Yu Y, Yang H, Cui W, Li J, Fan GG.	Dev Cogn Neurosci	2019
**575**	Paternal age contribution to brain white matter aberrations in autism spectrum disorder	Yassin W, Kojima M, Owada K, Kuwabara H, Gonoi W, Aoki Y, Takao H, Natsubori T, Iwashiro N, Kasai K, Kano Y, Abe O, Yamasue H.	Psychiatry Clin Neurosci	2019
**576**	Overlapping Anatomical Networks Convey Cross-Modal Suppression in the Sighted and Coactivation of "Visual" and Auditory Cortex in the Blind	Anurova I, Carlson S, Rauschecker JP.	Cereb Cortex	2019
**577**	White matter integrity in brain structures supporting semantic processing is associated with value-directed remembering in older adults	Hennessee JP, Reggente N, Cohen MS, Rissman J, Castel AD, Knowlton BJ.	Neuropsychologia	2019
**578**	Identification of Preoperative Language Tracts for Intrinsic Frontotemporal Diseases: A Pilot Reconstruction Algorithm in a Middle-Income Country	Ordóñez-Rubiano EG, Valderrama-Arias FA, Forbes JA, Johnson JM, Younus I, Marín-Muñoz JH, Sánchez-Montaño M, Angulo DA, Cifuentes-Lobelo HA, Cortes-Lozano W, Pedraza-Ciro MC, Bello-Dávila ML, Patiño-Gómez JG, Ordóñez-Mora EG.	World Neurosurg	2019

**579**	Diffuse Tract Damage in CADASIL Is Correlated with Global Cognitive Impairment	Ban S, Wang H, Wang M, Xu S, Qin Z, Su J, Du X, Liu JR.	Eur Neurol	2019
**580**	Association of olfaction dysfunction with brain microstructure in prodromal Parkinson disease	Sanjari Moghaddam H, Dolatshahi M, Salardini E, Aarabi MH.	Neurol Sci	2019
**581**	White matter alterations in adult with autism spectrum disorder evaluated using diffusion kurtosis imaging	Hattori A, Kamagata K, Kirino E, Andica C, Tanaka S, Hagiwara A, Fujita S, Maekawa T, Irie R, Kumamaru KK, Suzuki M, Wada A, Hori M, Aoki S.	Neuroradiology	2019
**582**	Relevance of brain lesion location for cognition in vascular mild cognitive impairment	Giorgio A, Di Donato I, De Leucio A, Zhang J, Salvadori E, Poggesi A, Diciotti S, Cosottini M, Ciulli S, Inzitari D, Pantoni L, Mascalchi M, Federico A, Dotti MT, De Stefano N; VMCI-Tuscany Study Group.	Neuroimage Clin	2019
**583**	Effect of aerobic exercise on white matter microstructure in the aging brain	Clark CM, Guadagni V, Mazerolle EL, Hill M, Hogan DB, Pike GB, Poulin MJ.	Behav Brain Res	2019
**584**	Neural structures supporting spontaneous and assisted (entrained) speech fluency	Bonilha L, Hillis AE, Wilmskoetter J, Hickok G, Basilakos A, Munsell B, Rorden C, Fridriksson J.	Brain	2019
**585**	Dorsal language stream anomalies in an inherited speech disorder	Liégeois FJ, Turner SJ, Mayes A, Bonthrone AF, Boys A, Smith L, Parry-Fielder B, Mandelstam S, Spencer-Smith M, Bahlo M, Scerri TS, Hildebrand MS, Scheffer IE, Connelly A, Morgan AT.	Brain	2019
**586**	Comparison of structural connectivity in Parkinson's disease with depressive symptoms versus non-depressed: a diffusion MRI connectometry study	Ansari M, Adib Moradi S, Ghazi Sherbaf F, Hedayatnia A, Aarabi MH.	Int Psychogeriatr	2019
**587**	White Matter Microstructural Change Contributes to Worse Cognitive Function in Patients With Type 2 Diabetes	Gao S, Chen Y, Sang F, Yang Y, Xia J, Li X, Zhang J, Chen K, Zhang Z.	Diabetes	2019
**588**	Functional imaging localization of complex organic hallucinations	Bernal B, Guillen M, Ragheb J, Altman N, Ibrahim GM, LaPlante P, Padilla A, Duchowny M.	Neurocase	2019
**589**	Mapping whole brain connectivity changes: The potential impact of different surgical resection approaches for temporal lobe epilepsy	Busby N, Halai AD, Parker GJM, Coope DJ, Lambon Ralph MA.	Cortex	2019
**590**	What is special about the human arcuate fasciculus? Lateralization, projections, and expansion	Eichert N, Verhagen L, Folloni D, Jbabdi S, Khrapitchev AA, Sibson NR, Mantini D, Sallet J, Mars RB.	Cortex	2019
**591**	White matter correlates of different	Rigon A, Voss MW, Turkstra LS, Mutlu B, Duff MC.	Soc Neurosci	2019

	aspects of facial affect recognition impairment following traumatic brain injury			
**592**	Increased Peripheral Interleukin 10 Relate to White Matter Integrity in Schizophrenia	Fu G, Zhang W, Dai J, Liu J, Li F, Wu D, Xiao Y, Shah C, Sweeney JA, Wu M, Lui S.	Front Neurosci	2019
**593**	White matter integrity is associated with gait impairment and falls in mild cognitive impairment. Results from the gait and brain study	Snir JA, Bartha R, Montero-Odasso M.	Neuroimage Clin	2019
**594**	Specifying the diffusion MRI connectome in Chinese-speaking children with developmental dyslexia and auditory processing deficits	Wang HS, Wang NY, Yeh FC.	Pediatr Neonatol	2019
**595**	Neuroanatomical structures supporting lexical diversity, sophistication, and phonological word features during discourse	Wilmskoetter J, Fridriksson J, Gleichgerrcht E, Stark BC, Delgaizo J, Hickok G, Vaden KI Jr, Hillis AE, Rorden C, Bonilha L.	Neuroimage Clin	2019
**596**	Effects of long-term adolescent alcohol consumption on white matter integrity and their correlations with metabolic alterations	Shen Q, Heikkinen N, Kärkkäinen O, Gröhn H, Könönen M, Liu Y, Kaarre O, Zhang Z, Tan C, Tolmunen T, Vanninen R.	Psychiatry Res Neuroimaging	2019
**597**	Electrically induced verbal perseveration: A striatal deafferentation model	Mandonnet E, Herbet G, Moritz-Gasser S, Poisson I, Rheault F, Duffau H.	Neurology	2019
**598**	The mis-wired language network in children with developmental language disorder: insights from DTI tractography	Verly M, Gerrits R, Sleurs C, Lagae L, Sunaert S, Zink I, Rommel N.	Brain Imaging Behav	2019
**599**	Multimodal MRI of grey matter, white matter, and functional connectivity in cognitively healthy mutation carriers at risk for frontotemporal dementia and Alzheimer's disease	Feis RA, Bouts MJRJ, Dopper EGP, Filippini N, Heise V, Trachtenberg AJ, van Swieten JC, van Buchem MA, van der Grond J, Mackay CE, Rombouts SARB.	BMC Neurol	2019
**600**	A huge benign fibrous histiocytoma arising from the renal capsule: report of a case	Sun C, Wang S, Li B, Sun X.	BMC Nephrol	2019
**601**	Diffusion Tensor Imaging And Tractography In Autistic, Dysphasic, And Healthy Control Children	Hrdlicka M, Sanda J, Urbanek T, Kudr M, Dudova I, Kickova S, Pospisilova L, Mohaplova M, Maulisova A, Krsek P, Kyncl M, Blatny M, Komarek V.	Neuropsychiatr Dis Treat	2019

**602**	Altered white matter connectivity in young people exposed to childhood abuse: a tract-based spatial statistics (TBSS) and tractography study	Lim L, Hart H, Howells H, Mehta MA, Simmons A, Mirza K, Rubia K.	J Psychiatry Neurosci	2019
**603**	Stimulating the inferior fronto-occipital fasciculus elicits complex visual hallucinations	Andelman-Gur MM, Gazit T, Strauss I, Fried I, Fahoum F.	Brain Stimul	2020
**604**	Microstructural disruption of the right inferior fronto-occipital and inferior longitudinal fasciculus contributes to WMH-related cognitive impairment	Chen HF, Huang LL, Li HY, Qian Y, Yang D, Qing Z, Luo CM, Li MC, Zhang B, Xu Y.	CNS Neurosci Ther	2020
**605**	Anatomical integrity within the inferior fronto-occipital fasciculus and semantic processing deficits in schizophrenia spectrum disorders	Surbeck W, Hänggi J, Scholtes F, Viher PV, Schmidt A, Stegmayer K, Studerus E, Lang UE, Riecher-Rössler A, Strik W, Seifritz E, Borgwardt S, Quednow BB, Walther S.	Schizophr Res	2020
**606**	Visual hallucinations and inferior longitudinal fasciculus in Parkinson's disease	Yuki N, Yoshioka A, Mizuhara R, Kimura T.	Brain Behav	2020
**607**	Shape analysis of the human association pathways	Yeh FC.	Neuroimage	2020
**608**	Prediction of Memory Impairment in Epilepsy Surgery by White Matter Diffusion	García-Pallero MA, Torres Díaz CV, Hernando CG, Plasencia PM, Manzanares R, García LE, Navas M, Pulido P, Delgado-Fernández J, Aragón Rubio JI, Sola RG.	World Neurosurg	2020
**609**	Pervasive White Matter Fiber Degeneration in Ischemic Stroke	Egorova N, Dhollander T, Khlif MS, Khan W, Werden E, Brodtmann A.	Stroke	2020
**610**	Microstructural Injury to Left-Sided Perisylvian White Matter Predicts Language Decline After Brain Radiation Therapy	Tibbs MD, Huynh-Le MP, Karunamuni R, Reyes A, Macari AC, Tringale KR, Salans M, Yip A, Liu E, Simon A, McDonald CR, Hattangadi-Gluth JA.	Int J Radiat Oncol Biol Phys	2020
**611**	Heschl's gyrus fiber intersection area: a new insight on the connectivity of the auditory-language hub	Fernández L, Velásquez C, García Porrero JA, de Lucas EM, Martino J.	Neurosurg Focus	2020
**612**	Anatomy and white matter connections of the lateral occipital cortex	Palejwala AH, O'Connor KP, Pelargos P, Briggs RG, Milton CK, Conner AK, Milligan TM, O'Donoghue DL, Glenn CA, Sughrue ME.	Surg Radiol Anat	2020
**613**	Diffusion tensor imaging in borderline personality disorder showing prefrontal white matter alterations	Nenadić I, Katzmann I, Besteher B, Langbein K, Güllmar D.	Compr Psychiatry	2020
**614**	White matter correlates of creative	Wertz CJ, Chohan MO, Ramey SJ, Flores RA, Jung RE.	Neuroimage	2020

	cognition in a normal cohort			
**615**	Change in Right Inferior Longitudinal Fasciculus Integrity Is Associated With Naming Recovery in Subacute Poststroke Aphasia	Blom-Smink M, Verly M, Spielmann K, Smits M, Ribbers GM, van de Sandt-Koenderman MWME.	Neurorehabil Neural Repair	2020
**616**	Persistent Differences in Brain Structure in Developmental Dyscalculia: A Longitudinal Morphometry Study	McCaskey U, von Aster M, O'Gorman R, Kucian K.	Front Hum Neurosci	2020
**617**	White matter integrity alternations associated with cocaine dependence and long-term abstinence: Preliminary findings	He Q, Li D, Turel O, Bechara A, Hser YI.	Behav Brain Res	2020
**618**	Linking individual differences in semantic cognition to white matter microstructure	Marino Dávolos J, Arias JC, Jefferies E.	Neuropsychologia	2020
**619**	Glioblastoma surgery related emotion recognition deficits are associated with right cerebral hemisphere tract changes	Sinha R, Dijkshoorn ABC, Li C, Manly T, Price SJ.	Brain Commun	2020
**620**	Neuroanatomical correlates of apathy and disinhibition in behavioural variant frontotemporal dementia	Sheelakumari R, Bineesh C, Varghese T, Kesavadas C, Verghese J, Mathuranath PS.	Brain Imaging Behav	2020
**621**	White Matter Asymmetry: A Reflection of Pathology in Traumatic Brain Injury	Vakhtin AA, Zhang Y, Wintermark M, Massaband P, Robinson MT, Ashford JW, Furst AJ.	J Neurotrauma	2020
**622**	Pinpointing Neural Correlates of Attachment in Poly-Drug Use: A Diffusion Tensor Imaging Study	Fuchshuber J, Unterrainer HF, Hiebler-Ragger M, Koschutnig K, Papousek I, Weiss EM, Fink A.	Front Neurosci	2020
**623**	White matter integrity disparities between normal-weight and overweight/obese adolescents: an automated fiber quantification tractography study	Carbine KA, Duraccio KM, Hedges-Muncy A, Barnett KA, Kirwan CB, Jensen CD.	Brain Imaging Behav	2020
**624**	Disruption of the structural and functional connectivity of the frontoparietal network underlies symptomatic anxiety in late-life depression	Li H, Lin X, Liu L, Su S, Zhu X, Zheng Y, Huang W, Que J, Shi L, Bao Y, Lu L, Deng J, Sun X.	Neuroimage Clin	2020
**625**	Language lateralization differences between left and right temporal lobe epilepsy as	Neudorf J, Kress S, Gould L, Gibb K, Mickleborough M, Borowsky R.	Epilepsy Behav	2020

	measured by overt word reading fMRI activation and DTI structural connectivity			
**626**	Tracking Inhibitory Control in Youth With ADHD: A Multi-Modal Neuroimaging Approach	Tremblay LK, Hammill C, Ameis SH, Bhaijiwala M, Mabbott DJ, Anagnostou E, Lerch JP, Schachar RJ.	Front Psychiatry	2020
**627**	Quantitative analyses of high-angular resolution diffusion imaging (HARDI)-derived long association fibers in children with sensorineural hearing loss	Shiohama T, Chew B, Levman J, Takahashi E.	Int J Dev Neurosci	2020
**628**	Anatomy and White Matter Connections of the Superior Frontal Gyrus	Briggs RG, Khan AB, Chakraborty AR, Abraham CJ, Anderson CD, Karas PJ, Bonney PA, Palejwala AH, Conner AK, O'Donoghue DL, Sughrue ME.	Clin Anat	2020
**629**	Changes in white matter microstructure related to non-linguistic cognitive impairment in post-stroke aphasia	Yao J, Liu X, Lu X, Xu C, Chen H, Zhang Y.	Neurol Res	2020
**630**	Anatomical Connectivity of the Visuospatial Attentional Network in Schizophrenia: A Diffusion Tensor Imaging Tractography Study	Leroux E, Poirel N, Dollfus S.	J Neuropsychiatry Clin Neurosci	2020
**631**	Assessment of White Matter Lesions in Parkinson's Disease: Voxel-based Analysis and Tract-based Spatial Statistics Analysis of Parkinson's Disease with Mild Cognitive Impairment	Pu W, Shen X, Huang M, Li Z, Zeng X, Wang R, Shen G, Yu H.	Curr Neurovasc Res	2020
**632**	Disrupted White Matter Integrity and Structural Brain Networks in Temporal Lobe Epilepsy With and Without Interictal Psychosis	Sone D, Sato N, Shigemoto Y, Kimura Y, Maikusa N, Ota M, Foong J, Koepp M, Matsuda H.	Front Neurol	2020
**633**	Disrupted Neural Synchrony Mediates the Relationship between White Matter Integrity and Cognitive Performance in Older Adults	Hinault T, Kraut M, Bakker A, Dagher A, Courtney SM.	Cereb Cortex	2020
**634**	Structural alterations associated with suicide attempts in major depressive disorder and bipolar disorder: A diffusion tensor imaging study	Wei S, Womer FY, Edmiston EK, Zhang R, Jiang X, Wu F, Kong L, Zhou Y, Tang Y, Wang F.	Prog Neuropsychopharmacol Biol Psychiatry	2020

**635**	The Impact of Early Deafness on Brain Plasticity: A Systematic Review of the White and Gray Matter Changes	Simon M, Campbell E, Genest F, MacLean MW, Champoux F, Lepore F.	Front Neurosci	2020
**636**	Transcorticosubcortical Approach for Left Posterior Mediobasal Temporal Region Gliomas: A Case Series and Anatomic Review of Relevant White Matter Tracts	Brown DA, Hanalioglu S, Chaichana K, Duffau H.	World Neurosurg	2020
**637**	Aberrant structural connectivity in childhood maltreatment: A meta-analysis	Lim L, Howells H, Radua J, Rubia K.	Neurosci Biobehav Rev	2020
**638**	White Matter Microstructure Changes and Cognitive Impairment in the Progression of Chronic Kidney Disease	Liu M, Wu Y, Wu X, Ma X, Yin Y, Fang H, Huang S, Su H, Jiang G.	Front Neurosci	2020
**639**	BDNF Serum Levels are Associated With White Matter Microstructure in Schizophrenia - A Pilot Study	Hammans C, Neugebauer K, Kumar V, Mevissen L, Sternkopf MA, Novakovic A, Wensing T, Habel U, Abel T, Nickl-Jockschat T.	Front Psychiatry	2020
**640**	White matter tract myelin maturation and its association with general psychopathology in adolescence and early adulthood	Vanes LD, Moutoussis M, Ziegler G, Goodyer IM, Fonagy P, Jones PB, Bullmore ET; NSPN Consortium; Dolan RJ.	Hum Brain Mapp	2020
**641**	Association between diffusivity measures and language and cognitive-control abilities from early toddler's age to childhood	Farah R, Tzafrir H, Horowitz-Kraus T.	Brain Struct Funct	2020
**642**	Functional connectivity and microstructural changes of the brain in primary Sjögren syndrome: the relationship with depression	Andrianopoulou A, Zikou AK, Astrakas LG, Gerolymatou N, Xydis V, Voulgari P, Kiortsis DN, Argyropoulou MI.	Acta Radiol	2020
**643**	Uncinate fasciculus disruption relates to poor recognition of negative facial emotions in Alzheimer's disease: a cross-sectional diffusion tensor imaging study	Takahashi M, Kitamura S, Matsuoka K, Yoshikawa H, Yasuno F, Makinodan M, Kimoto S, Miyasaka T, Kichikawa K, Kishimoto T.	Psychogeriatrics	2020
**644**	Long Longitudinal Tract Lesion Contributes to the Progression of Alzheimer's Disease	Luo C, Li M, Qin R, Chen H, Huang L, Yang D, Ye Q, Liu R, Xu Y, Zhao H, Bai F.	Front Neurol	2020

**645**	Missing the forest because of the trees: slower alternations during binocular rivalry are associated with lower levels of visual detail during ongoing thought	Ho NSP, Baker D, Karapanagiotidis T, Seli P, Wang HT, Leech R, Bernhardt B, Margulies D, Jefferies E, Smallwood J.	Neurosci Conscious	2020
**646**	Association between white matter impairment and cognitive dysfunction in patients with ischemic Moyamoya disease	Liu Z, He S, Xu Z, Duan R, Yuan L, Xiao C, Yi Z, Wang R.	BMC Neurol	2020
**647**	Fixel-Based Diffusion Magnetic Resonance Imaging Reveals Novel Associations Between White Matter Microstructure and Childhood Aggressive Behavior	Grazioplene R, Tseng WL, Cimino K, Kalvin C, Ibrahim K, Pelphrey KA, Sukhodolsky DG.	Biol Psychiatry Cogn Neurosci Neuroimaging	2020
**648**	Abnormalities in the white matter tracts in patients with Parkinson disease and psychosis	Lenka A, Ingalhalikar M, Shah A, Saini J, Arumugham SS, Hegde S, George L, Yadav R, Pal PK.	Neurology	2020
**649**	Structural white and gray matter differences in a large sample of patients with Posttraumatic Stress Disorder and a healthy and trauma-exposed control group: Diffusion tensor imaging and region-based morphometry	Siehl S, Wicking M, Pohlack S, Winkelmann T, Zidda F, Steiger-White F, King J, Burgess N, Flor H, Nees F.	Neuroimage Clin	2020
**650**	White Matter Abnormalities Based on TBSS and Its Correlation With Impulsivity Behavior of Methamphetamine Addicts	Huang S, Yang W, Luo J, Yan C, Liu J.	Front Psychiatry	2020
**651**	Characteristic patterns of white matter tract injury in sport-related concussion: An image based meta-analysis	Hellewell SC, Nguyen VPB, Jayasena RN, Welton T, Grieve SM.	Neuroimage Clin	2020
**652**	Regional White Matter Integrity Predicts Treatment Response to Escitalopram and Memantine in Geriatric Depression: A Pilot Study	Krause-Sorio B, Siddarth P, Milillo MM, Vlasova R, Ercoli L, Narr KL, Lavretsky H.	Front Psychiatry	2020
**653**	Localizing deficits in white matter tracts of patients with narcolepsy with cataplexy: tract-specific statistical analysis	Park HR, Kim HR, Seong JK, Joo EY.	Brain Imaging Behav	2020
**654**	Age-related differences in white matter diffusion measures in	Thompson A, Shahidiani A, Fritz A, O'Muircheartaigh J, Walker L, D'Almeida V,	Mol Autism	2020

	autism spectrum condition	Murphy C, Daly E, Murphy D, Williams S, Deoni S, Ecker C.		
**655**	White matter microstructure mediates the association between prenatal exposure to phthalates and behavior problems in preschool children	England-Mason G, Grohs MN, Reynolds JE, MacDonald A, Kinniburgh D, Liu J, Martin JW, Lebel C, Dewey D; APrON Study Team.	Environ Res	2020
**656**	White Matter Connectivity in Youth at Risk for Serious Mental Illness: A Longitudinal Analysis	Shakeel MK, MacQueen G, Addington J, Metzak PD, Georgopoulos G, Bray S, Goldstein BI, MacIntosh BJ, Wang J, Kennedy SH, Lebel C.	Psychiatry Res Neuroimaging	2020
**657**	Disrupted structural brain connectome underlying the cognitive deficits in remitted late-onset depression	Wang Z, Yuan Y, You J, Zhang Z.	Brain Imaging Behav	2020
**658**	White Matter Microstructural Damage as an Early Sign of Subjective Cognitive Decline	Luo C, Li M, Qin R, Chen H, Yang D, Huang L, Liu R, Xu Y, Bai F, Zhao H.	Front Aging Neurosci	2020
**659**	Theory of mind network in multiple Sclerosis: A double disconnection mechanism	Isernia S, Cabinio M, Pirastru A, Mendozzi L, Di Dio C, Marchetti A, Massaro D, Baglio F.	Soc Neurosci	2020
**660**	White Matter Hyperintensities Related to Parkinson's Disease Executive Function	Linortner P, McDaniel C, Shahid M, Levine TF, Tian L, Cholerton B, Poston KL.	Mov Disord Clin Pract	2020
**661**	Convergent creative thinking performance is associated with white matter structures: Evidence from a large sample study	Takeuchi H, Taki Y, Matsudaira I, Ikeda S, Dos S Kawata KH, Nouchi R, Sakaki K, Nakagawa S, Nozawa T, Yokota S, Araki T, Hanawa S, Ishibashi R, Yamazaki S, Kawashima R.	Neuroimage	2020
**662**	Micro-structural white matter abnormalities and cognitive impairment in asymptomatic carotid plaque patients: A DTI study using TBSS analysis	Liu L, Huang Q, Yang S, Wen Y, He W, Liu H, Meng L, Jiang H, Xia J, Liao W, Liu Y.	Clin Neurol Neurosurg	2020
**663**	Network reorganisation following anterior temporal lobe resection and relation with post-surgery seizure relapse: A longitudinal study	da Silva NM, Forsyth R, McEvoy A, Miserocchi A, de Tisi J, Vos SB, Winston GP, Duncan J, Wang Y, Taylor PN.	Neuroimage Clin	2020
**664**	Adrenarcheal hormone-related development of white matter during late childhood	Barendse MEA, Simmons JG, Smith RE, Seal ML, Whittle S.	Neuroimage	2020
**665**	A microstructural neural network	Valeriani D, Simonyan K.	Proc Natl Acad Sci U S A	2020

	biomarker for dystonia diagnosis identified by a DystoniaNet deep learning platform			
**666**	Multimodal principal component analysis to identify major features of white matter structure and links to reading	Geeraert BL, Chamberland M, Lebel RM, Lebel C.	PLoS One	2020
**667**	Neuroanatomical correlates of impulsive traits in children aged 9 to 10	Owens MM, Hyatt CS, Gray JC, Miller JD, Lynam DR, Hahn S, Allgaier N, Potter A, Garavan H.	J Abnorm Psychol	2020
**668**	Association of Poorer Hearing With Longitudinal Change in Cerebral White Matter Microstructure	Armstrong NM, Williams OA, Landman BA, Deal JA, Lin FR, Resnick SM.	JAMA Otolaryngol Head Neck Surg	2020
**669**	Game theoretical mapping of white matter contributions to visuospatial attention in stroke patients with hemineglect	Toba MN, Zavaglia M, Malherbe C, Moreau T, Rastelli F, Kaglik A, Valabrègue R, Pradat-Diehl P, Hilgetag CC, Valero-Cabré A.	Hum Brain Mapp	2020
**670**	Characterizing White Matter Tract Organization in Polymicrogyria and Lissencephaly: A Multifiber Diffusion MRI Modeling and Tractography Study	Arrigoni F, Peruzzo D, Mandelstam S, Amorosino G, Redaelli D, Romaniello R, Leventer R, Borgatti R, Seal M, Yang JY.	AJNR Am J Neuroradiol	2020
**671**	Brain structural correlates of depressive symptoms in Parkinson's disease patients at different disease stage	Li Y, Huang P, Guo T, Guan X, Gao T, Sheng W, Zhou C, Wu J, Song Z, Xuan M, Gu Q, Xu X, Yang Y, Zhang M.	Psychiatry Res Neuroimaging	2020
**672**	Fiber-specific white matter reductions in Parkinson hallucinations and visual dysfunction	Zarkali A, McColgan P, Leyland LA, Lees AJ, Rees G, Weil RS.	Neurology	2020
**673**	White matter microstructural alterations in posttraumatic stress disorder: An ROI and whole-brain based meta-analysis	Ju Y, Ou W, Su J, Averill CL, Liu J, Wang M, Wang Z, Zhang Y, Liu B, Li L, Abdallah CG.	J Affect Disord	2020
**674**	Long-term development of white matter fibre density and morphology up to 13 years after preterm birth: A fixel-based analysis	Kelly CE, Thompson DK, Genc S, Chen J, Yang JY, Adamson C, Beare R, Seal ML, Doyle LW, Cheong JL, Anderson PJ.	Neuroimage	2020
**675**	Visual pathways evaluation in Kearns Sayre syndrome: a diffusion tensor imaging study	Rossi-Espagnet MC, Lucignani M, Pasquini L, Napolitano A, Pro S, Romano A, Diodato D, Martinelli D, Longo D.	Neuroradiology	2020

**676**	Temporal lobe structural evaluation after transsylvian selective amygdalohippocampectomy	Giacomini L, de Souza JPSA, Formentin C, de Campos BM, Todeschini AB, de Oliveira E, Tedeschi H, Joaquim AF, Cendes F, Ghizoni E.	Neurosurg Focus	2020
**677**	Developmental Differences of Structural Connectivity and Effective Connectivity in Semantic Judgments of Chinese Characters	Fan LY, Lo YC, Hsu YC, Chen YJ, Tseng WI, Chou TL.	Front Hum Neurosci	2020
**678**	Abnormal brain white matter in patients with hemifacial spasm: a diffusion tensor imaging study	Guo C, Xu H, Niu X, Krimmel S, Liu J, Gao L, Zhang M, Wang Y.	Neuroradiology	2020
**679**	Combined structural and diffusion tensor imaging detection of ischemic injury in moyamoya disease: relation to disease advancement and cerebral hypoperfusion	Kazumata K, Tokairin K, Ito M, Uchino H, Sugiyama T, Kawabori M, Osanai T, Tha KK, Houkin K.	J Neurosurg	2020
**680**	fMRI-Targeted High-Angular Resolution Diffusion MR Tractography to Identify Functional Language Tracts in Healthy Controls and Glioma Patients	Sanvito F, Caverzasi E, Riva M, Jordan KM, Blasi V, Scifo P, Iadanza A, Crespi SA, Cirillo S, Casarotti A, Leonetti A, Puglisi G, Grimaldi M, Bello L, Gorno-Tempini ML, Henry RG, Falini A, Castellano A.	Front Neurosci	2020
**681**	Assessing the connectional anatomy of superior and lateral surgical approaches for medial temporal lobe epilepsy	Baran O, Balak N, Baydin S, Aydin I, Kayhan A, Evran S, Kemerdere R, Tanriover N.	J Clin Neurosci	2020
**682**	Support vector machine based classification of smokers and nonsmokers using diffusion tensor imaging	Zhao M, Liu J, Cai W, Li J, Zhu X, Yu D, Yuan K.	Brain Imaging Behav	2020
**683**	Plasticity in language cortex and white matter tracts after resection of dominant inferior parietal lobule arteriovenous malformations: a combined fMRI and DTI study	Jiao Y, Lin F, Wu J, Li H, Fu W, Huo R, Cao Y, Wang S, Zhao J.	J Neurosurg	2020
**684**	Combining Electrostimulation With Fiber Tracking to Stratify the Inferior Fronto-Occipital Fasciculus	Roux A, Lemaitre AL, Deverdun J, Ng S, Duffau H, Herbet G.	Front Neurosci	2021
**685**	Altered microstructural pattern of white matter in Cushing's	Cui M, Zhou T, Feng S, Liu X, Wang F, Zhang Y, Yu X.	Neuroimage Clin	2021

	disease identified by automated fiber quantification			
**686**	Neuroanatomic Volume Differences in Tinnitus and Hearing Loss	Yousef A, Hinkley LB, Nagarajan SS, Cheung SW.	Laryngoscope	2021
**687**	The ventral pathway of the human brain: A continuous association tract system	Weiller C, Reisert M, Peto I, Hennig J, Makris N, Petrides M, Rijntjes M, Egger K.	Neuroimage	2021
**688**	The extreme capsule and aphasia: proof-of-concept of a new way relating structure to neurological symptoms	Martinez Oeckel A, Rijntjes M, Glauche V, Kümmerer D, Kaller CP, Egger K, Weiller C.	Brain Commun	2021
**689**	Risk Assessment by Pre-surgical Tractography in Left Hemisphere Low-Grade Gliomas	Ius T, Somma T, Baiano C, Guarracino I, Pauletto G, Nilo A, Maieron M, Palese F, Skrap M, Tomasino B.	Front Neurol	2021
**690**	Widespread White Matter Alterations in Patients With Visual Snow Syndrome	Michels L, Stämpfli P, Aldusary N, Piccirelli M, Freund P, Weber KP, Fierz FC, Kollias S, Traber G.	Front Neurol	2021
**691**	Cortical regions and networks of hyperkinetic seizures: Electrocorticography and diffusion tensor imaging study	Sasagawa A, Enatsu R, Kuribara T, Arihara M, Hirano T, Ochi S, Mikuni N.	Epilepsy Behav	2021
**692**	Neural Underpinnings of Proactive Interference in Working Memory: Evidence From Patients With Unilateral Lesions	Ries SK, Schendel KL, Herron TJ, Dronkers NF, Baldo JV, Turken AU.	Front Neurol	2021
**693**	Towards a tractography-based risk stratification model for language area associated gliomas	Tuncer MS, Salvati LF, Grittner U, Hardt J, Schilling R, Bährend I, Silva LL, Fekonja LS, Faust K, Vajkoczy P, Rosenstock T, Picht T.	Neuroimage Clin	2021
**694**	White Matter Abnormalities Associated With Prolonged Recovery in Adolescents Following Concussion	Lima Santos JP, Kontos AP, Mailliard S, Eagle SR, Holland CL, Suss SJ Jr, Abdul-Waalee H, Stiffler RS, Bitzer HB, Blaney NA, Colorito AT, Santucci CG, Brown A, Kim T, Iyengar S, Skeba A, Diler RS, Ladouceur CD, Phillips ML, Brent D, Collins MW, Versace A.	Front Neurol	2021
**695**	Anatomy and White Matter Connections of the Lingual Gyrus and Cuneus	Palejwala AH, Dadario NB, Young IM, O'Connor K, Briggs RG, Conner AK, O'Donoghue DL, Sughrue ME.	World Neurosurg	2021
**696**	Anatomy and White Matter Connections of the Middle Frontal Gyrus	Briggs RG, Lin YH, Dadario NB, Kim SJ, Young IM, Bai MY, Dhanaraj V, Fonseka RD, Hormovas J, Tanglay O, Chakraborty AR, Milligan TM, Abraham CJ, Anderson CD, Palejwala AH, Conner AK, O'Donoghue DL, Sughrue ME.	World Neurosurg	2021
**697**	Tracking white-matter brain modifications in chronic non-bothersome acoustic trauma tinnitus	Jaroszynski C, Attyé A, Job A, Delon-Martin C.	Neuroimage Clin	2021
**698**	Fixel-based evidence of microstructural damage in crossing	Zhang J, Zheng W, Shang D, Chen Y, Zhong S, Ye J, Li L, Yu Y, Zhang L, Cheng R, He F, Wu D, Ye X, Luo B.	Neuroimage Clin	2021

	pathways improves language mapping in Post-stroke aphasia			
**699**	Composition and organization of the sagittal stratum in the human brain: a fiber dissection study	Maldonado IL, Destrieux C, Ribas EC, Siqueira de Abreu Brito Guimarães B, Cruz PP, Duffau H.	J Neurosurg	2021
**700**	Spatiotemporal changes in diffusivity and anisotropy in fetal brain tractography	Machado-Rivas F, Afacan O, Khan S, Marami B, Velasco-Annis C, Lidov H, Warfield SK, Gholipour A, Jaimes C.	Hum Brain Mapp	2021
**701**	Anatomy and White Matter Connections of the Superior Parietal Lobule	Lin YH, Dadario NB, Hormovas J, Young IM, Briggs RG, MacKenzie AE, Palejwala AH, Fonseka RD, Kim SJ, Tanglay O, Fletcher LR, Abraham CJ, Conner AK, O'Donoghue DL, Sughrue ME.	Oper Neurosurg (Hagerstown)	2021
**702**	Predicted disconnectome associated with progressive periventricular white matter ischemia	Li Z, Dolui S, Habes M, Bassett DS, Wolk D, Detre JA.	Cereb Circ Cogn Behav	2021
**703**	In vivo microstructural white matter changes in early spinocerebellar ataxia 2	Stezin A, Bhardwaj S, Khokhar S, Hegde S, Jain S, Bharath RD, Saini J, Pal PK.	Acta Neurol Scand	2021
**704**	Benefit of Action Naming Over Object Naming for Visualization of Subcortical Language Pathways in Navigated Transcranial Magnetic Stimulation-Based Diffusion Tensor Imaging-Fiber Tracking	Ohlerth AK, Bastiaanse R, Negwer C, Sollmann N, Schramm S, Schröder A, Krieg SM.	Front Hum Neurosci	2021
**705**	Associations of subclinical autistic-like traits with brain structural variation using diffusion tensor imaging and voxel-based morphometry	Schröder Y, Hohmann DM, Meller T, Evermann U, Pfarr JK, Jansen A, Kamp-Becker I, Grezellschak S, Nenadić I.	Eur Psychiatry	2021
**706**	Childhood conduct problems are associated with reduced white matter fibre density and morphology	Burley DT, Genc S, Silk TJ.	J Affect Disord	2021
**707**	Long-term sequelae of herpes simplex virus encephalitis-related white matter injury: correlation of neuropsychological outcome and diffusion tensor imaging	Medhi G, Kapadia A, Parida S, C D, Bagepalli BS, M N, Kumar K, Gupta AK, Saini J.	J Neurovirol	2021
**708**	Associations between sarcopenia and white matter alterations in older adults with diabetes mellitus: A diffusion tensor imaging study	Tamura Y, Shimoji K, Ishikawa J, Tachibana A, Kodera R, Oba K, Toyoshima K, Chiba Y, Tokumaru AM, Araki A.	J Diabetes Investig	2021
**709**	White matter microstructure	Zhou C, Dong M, Duan W, Lin H, Wang S, Wang Y, Zhang Y, Shi J, Liu S, Cheng Y, Xu X, Xu J.	Lupus	2021

	alterations in systemic lupus erythematosus: A preliminary coordinate-based meta-analysis of diffusion tensor imaging studies			
**710**	Brain structural connectivity, anhedonia, and phenotypes of major depressive disorder: A structural equation model approach	Pfarr JK, Brosch K, Meller T, Ringwald KG, Schmitt S, Stein F, Meinert S, Grotegerd D, Thiel K, Lemke H, Winter A, Waltemate L, Hahn T, Opel N, Repple J, Bauer J, Jansen A, Dannlowski U, Krug A, Kircher T, Nenadić I.	Hum Brain Mapp	2021
**711**	Second Language Learning in Older Adults: Effects on Brain Structure and Predictors of Learning Success	Nilsson J, Berggren R, Garzón B, Lebedev AV, Lövdén M.	Front Aging Neurosci	2021
**712**	Association between Uncinate Fasciculus Integrity and Agoraphobia Symptoms in Female Patients with Panic Disorder	Kim SE, Bang M, Won E, Lee SH.	Clin Psychopharmacol Neurosci	2021
**713**	A Longitudinal Study of White Matter Functional Network in Mild Traumatic Brain Injury	Jia X, Chang X, Bai L, Wang Y, Dong D, Gan S, Wang S, Li X, Yang X, Sun Y, Li T, Xiong F, Niu X, Yan H.	J Neurotrauma	2021
**714**	Tract Specificity of Age Effects on Diffusion Tensor Imaging Measures of White Matter Health	Matijevic S, Ryan L.	Front Aging Neurosci	2021
**715**	White Matter Differences in Networks in Elders with Mild Cognitive Impairment and Alzheimer's Disease	Yang FPG, Bal SS, Lee JF, Chen CC.	Brain Connect	2021
**716**	Associations among diffusion tensor imaging and neurocognitive function in survivors of pediatric brain tumor: A pilot study	Aleksonis HA, Wier R, Pearson MM, Cannistraci CJ, Anderson AW, Kuttesch JF, Compas BE, Hoskinson KR.	Appl Neuropsychol Child	2021
**717**	A connectivity model of the anatomic substrates underlying ideomotor apraxia: A meta-analysis of functional neuroimaging studies	O'Neal CM, Ahsan SA, Dadario NB, Fonseka RD, Young IM, Parker A, Maxwell BD, Yeung JT, Briggs RG, Teo C, Sughrue ME.	Clin Neurol Neurosurg	2021
**718**	Lesion-symptom mapping of language impairments in patients suffering from left perisylvian gliomas	Fekonja LS, Wang Z, Doppelbauer L, Vajkoczy P, Picht T, Pulvermüller F, Dreyer FR.	Cortex	2021
**719**	The Relevant Role of Navigated Tractography in Speech Eloquent Area	Salvati LF, De Marco R, Palmieri G, Minardi M, Massara A, Pesaresi A, Cagetti B, Melcarne A, Garbossa D.	Brain Sci	2021

	Glioma Surgery: Single Center Experience			
**720**	Replicable association between human cytomegalovirus infection and reduced white matter fractional anisotropy in major depressive disorder	Zheng H, Bergamino M, Ford BN, Kuplicki R, Yeh FC, Bodurka J, Burrows K; Tulsa 1000 Investigators; Hunt PW, Teague TK, Irwin MR, Yolken RH, Paulus MP, Savitz J.	Neuropsychopharmacology	2021
**721**	Neonatal White Matter Microstructure and Emotional Development during the Preschool Years in Children Who Were Born Very Preterm	Kanel D, Vanes LD, Pecheva D, Hadaya L, Falconer S, Counsell SJ, Edwards DA, Nosarti C.	eNeuro	2021
**722**	Predicting the Extent of Resection in Low-Grade Glioma by Using Intratumoral Tractography to Detect Eloquent Fascicles Within the Tumor	Mato D, Velasquez C, Gómez E, Marco de Lucas E, Martino J.	Neurosurgery	2021
**723**	The utility of diffusion tractography for speech preservation in laser ablation of the dominant insula: illustrative case	Kaufmann TJ, Lehman VT, Wong-Kisiel LC, Kerezoudis P, Miller KJ.	J Neurosurg Case Lessons	2021
**724**	Audiovisual structural connectivity in musicians and non-musicians: a cortical thickness and diffusion tensor imaging study	Møller C, Garza-Villarreal EA, Hansen NC, Højlund A, Bærentsen KB, Chakravarty MM, Vuust P.	Sci Rep	2021
**725**	The Associations Between White Matter Disruptions and Cognitive Decline at the Early Stage of Subcortical Vascular Cognitive Impairment: A Case-Control Study	Qiao Y, He X, Zhang J, Liang Y, Shao W, Zhang Z, Zhang S, Peng D.	Front Aging Neurosci	2021
**726**	White matter abnormalities in a patient with visual snow syndrome: New evidence from a diffusion tensor imaging study	Latini F, Fahlström M, Marklund N, Feresiadou A.	Eur J Neurol	2021
**727**	Diffuse Tract Damage Correlates With Global Cognitive Impairment in Cerebral Autosomal Dominant Arteriopathy With Subcortical Infarcts and Leukoencephalopathy: A Tract-Based Spatial Statistics Study	Zhang Q, Wang D, Wu S, Ren Y, Li Y, Zhang J, Feng X.	J Comput Assist Tomogr	2021
**728**	White-Matter Neuroanatomical Predictors of Aphasic Verb Retrieval	Dresang HC, Hula WD, Yeh FC, Warren T, Dickey MW.	Brain Connect	2021

**729**	New insights into the anatomo-functional architecture of the right sagittal stratum and its surrounding pathways: an axonal electrostimulation mapping study	Berro DH, Herbet G, Duffau H.	Brain Struct Funct	2021
**730**	Individual differences in interoceptive accuracy and prediction error in motor functional neurological disorders: A DTI study	Sojka P, Diez I, Bareš M, Perez DL.	Hum Brain Mapp	2021
**731**	Orientational changes of white matter fibers in Alzheimer's disease and amnestic mild cognitive impairment	Zhao H, Cheng J, Liu T, Jiang J, Koch F, Sachdev PS, Basser PJ, Wen W; Alzheimer's Disease Neuroimaging Initiative.	Hum Brain Mapp	2021
**732**	Plasma β-Amyloid Levels Associated With Structural Integrity Based on Diffusion Tensor Imaging in Subjective Cognitive Decline: The SILCODE Study	Wang X, Zhao M, Lin L, Han Y.	Front Aging Neurosci	2021
**733**	Widespread white matter aberration is associated with the severity of apathy in amnestic Mild Cognitive Impairment: Tract-based spatial statistics analysis	Setiadi TM, Martens S, Opmeer EM, Marsman JC, Tumati S, Reesink FE, De Deyn PP, Aleman A, Ćurčić-Blake B.	Neuroimage Clin	2021
**734**	Brain structure associations with phonemic and semantic fluency in typically-developing children	Gonzalez MR, Baaré WFC, Hagler DJ Jr, Archibald S, Vestergaard M, Madsen KS.	Dev Cogn Neurosci	2021
**735**	White matter microstructure and connectivity in patients with obsessive-compulsive disorder and their unaffected siblings	Dikmeer N, Besiroglu L, Di Biase MA, Zalesky A, Kasal MI, Bilge A, Durmaz E, Polat S, Gelal F, Zorlu N.	Acta Psychiatr Scand	2021
**736**	Association of Gyrification Pattern, White Matter Changes, and Phenotypic Profile in Patients With Parkinson Disease	Tang X, Zhang Y, Liu D, Hu Y, Jiang L, Zhang J.	Neurology	2021
**737**	The Correlation of Reduced Fractional Anisotropy in the Cingulum With Suicide Risk in Bipolar Disorder	Tian F, Wang X, Long X, Roberts N, Feng C, Yue S, Jia Z.	Front Psychiatry	2021
**738**	Altered white matter microarchitecture in Parkinson's disease: a voxel-based meta-	Suo X, Lei D, Li W, Li L, Dai J, Wang S, Li N, Cheng L, Peng R, Kemp GJ, Gong Q.	Front Med	2021

	analysis of diffusion tensor imaging studies			
**739**	Posterior Cortical Cognitive Deficits Are Associated With Structural Brain Alterations in Mild Cognitive Impairment in Parkinson's Disease	Devignes Q, Viard R, Betrouni N, Carey G, Kuchcinski G, Defebvre L, Leentjens AFG, Lopes R, Dujardin K.	Front Aging Neurosci	2021
**740**	Tract-specific analysis and neurocognitive functioning in sickle cell patients without history of overt stroke	Chai Y, Ji C, Coloigner J, Choi S, Balderrama M, Vu C, Tamrazi B, Coates T, Wood JC, O'Neil SH, Lepore N.	Brain Behav	2021
**741**	White matter alterations in Parkinson's disease with levodopa-induced dyskinesia	Ogawa T, Hatano T, Kamagata K, Andica C, Takeshige-Amano H, Uchida W, Saito Y, Shimo Y, Oyama G, Umemura A, Iwamuro H, Ito M, Hori M, Aoki S, Hattori N.	Parkinsonism Relat Disord	2021
**742**	Modern Technology in Multi-Shell Diffusion MRI Reveals Diffuse White Matter Changes in Young Adults With Relapsing-Remitting Multiple Sclerosis	Beaudoin AM, Rheault F, Theaud G, Laberge F, Whittingstall K, Lamontagne A, Descoteaux M.	Front Neurosci	2021
**743**	White matter abnormalities in misophonia	Eijsker N, Schröder A, Liebrand LC, Smit DJA, van Wingen G, Denys D.	Neuroimage Clin	2021
**744**	Disrupting self-evaluative processing with electrostimulation mapping during awake brain surgery	Ng S, Herbet G, Lemaitre AL, Moritz-Gasser S, Duffau H.	Sci Rep	2021
**745**	Alterations in white matter integrity and asymmetry in patients with benign childhood epilepsy with centrotemporal spikes and childhood absence epilepsy: An automated fiber quantification tractography study	Shu M, Yu C, Shi Q, Li Y, Niu K, Zhang S, Wang X.	Epilepsy Behav	2021
**746**	White matter alterations in young children with prenatal alcohol exposure	Kar P, Reynolds JE, Grohs MN, Gibbard WB, McMorris C, Tortorelli C, Lebel C.	Dev Neurobiol	2021
**747**	Cerebral white matter connectivity, cognition, and age-related macular degeneration	Zhuang J, Madden DJ, Cunha P, Badea A, Davis SW, Potter GG, Lad EM, Cousins SW, Chen NK, Allen K, Maciejewski AJ, Fernandez XD, Diaz MT, Whitson HE.	Neuroimage Clin	2021
**748**	Surgery of Insular Diffuse Gliomas-Part 2: Probabilistic Cortico-Subcortical Atlas of Critical Eloquent Brain Structures and Probabilistic Resection Map During Transcortical Awake Resection	Pallud J, Roux A, Trancart B, Peeters S, Moiraghi A, Edjlali M, Oppenheim C, Varlet P, Chrétien F, Dhermain F, Zanello M, Dezamis E.	Neurosurgery	2021
**749**	Transopercular Insular Approach, Overcoming the Training Curve Using a	Santos C, Velasquez C, Esteban J, Fernandez L, Mandonnet E, Duffau H, Martino J.	Oper Neurosurg (Hagerstown)	2021

	Cadaveric Simulation Model: 2-Dimensional Operative Video			
**750**	White matter microstructure in youth at risk for serious mental illness: A comparative analysis	Shakeel MK, Hassel S, Davis AD, Metzak PD, MacQueen GM, Arnott SR, Bray S, Frey BN, Goldstein BI, Hall GB, Harris J, Lam RW, MacIntosh BJ, Milev R, Mueller DJ, Rotzinger S, Strother SC, Wang J, Zamyadi M, Kennedy SH, Addington J, Lebel C.	Psychiatry Res Neuroimaging	2021
**751**	Neurobiological substrates of major psychiatry disorders: transdiagnostic associations between white matter abnormalities, neuregulin 1 and clinical manifestation	Duan J, Wei Y, Womer FY, Zhang X, Chang M, Zhu Y, Liu Z, Li C, Yin Z, Zhang R, Sun J, Wang P, Wang S, Jiang X, Wei S, Zhang Y, Tang Y, Wang F.	J Psychiatry Neurosci	2021
**752**	The Role of 3D Tractography in Skull Base Surgery: Technological Advances, Feasibility, and Early Clinical Assessment with Anterior Skull Base Meningiomas	Chakravarthi SS, Fukui MB, Monroy-Sosa A, Gonen L, Epping A, Jennings JE, Mena LPSR, Khalili S, Singh M, Celix JM, Kura B, Kojis N, Rovin RA, Kassam AB.	J Neurol Surg B Skull Base	2021
**753**	White matter tract-specific alterations in patients with primary restless legs syndrome	Park HR, Kim HR, Oh S, Seong JK, Joo EY.	Sci Rep	2021
**754**	White Matter Changes With Rehabilitation in Children With Developmental Coordination Disorder: A Randomized Controlled Trial	Izadi-Najafabadi S, Zwicker JG.	Front Hum Neurosci	2021
**755**	White matter abnormalities in adults with bipolar disorder type-II and unipolar depression	Manelis A, Soehner A, Halchenko YO, Satz S, Ragozzino R, Lucero M, Swartz HA, Phillips ML, Versace A.	Sci Rep	2021
**756**	Diffusion MRI of the infant brain reveals unique asymmetry patterns during the first-half-year of development	Liu T, Gao F, Zheng W, You Y, Zhao Z, Lv Y, Chen W, Zhang H, Ji C, Wu D.	Neuroimage	2021
**757**	Transsylvian amygdalohippocampectomy for mesial temporal lobe epilepsy: Comparison of three different approaches	de Souza JPSAS, Pimentel-Silva LR, Ayub G, Nogueira MH, Zanao T, Yasuda CL, Campos BM, Rogerio F, Tedeschi H, Cendes F, Ghizoni E.	Epilepsia	2021
**758**	Motor function and white matter connectivity in children cooled for neonatal encephalopathy	Spencer APC, Brooks JCW, Masuda N, Byrne H, Lee-Kelland R, Jary S, Thoresen M, Goodfellow M, Cowan FM, Chakkarapani E.	Neuroimage Clin	2021
**759**	Microsurgical and Tractographic Anatomical Study of Transtemporal-Transchoroidal Fissure	Egemen E, Celtikci P, Dogruel Y, Yakar F, Sahinoglu D, Farouk M, Adiguzel E, Ugur HC, Coskun E, Güngör A.	Oper Neurosurg (Hagerstown)	2021

	Approaches to the Ambient Cistern			
**760**	Prosopagnosia following nonlanguage dominant inferior temporal lobe low-grade glioma resection in which the inferior longitudinal fasciculus was disrupted preoperatively: illustrative case	Young JS, Morshed RA, Andrews JP, Cha S, Berger MS.	J Neurosurg Case Lessons	2021
**761**	Frontal cortical regions associated with attention connect more strongly to central than peripheral V1	Sims SA, Demirayak P, Cedotal S, Visscher KM.	Neuroimage	2021
**762**	Caffeine exposure in utero is associated with structural brain alterations and deleterious neurocognitive outcomes in 9-10 year old children	Christensen ZP, Freedman EG, Foxe JJ.	Neuropharmacology	2021
**763**	Inferior fronto-occipital fascicle displacement in temporoinsular gliomas using diffusion tensor imaging	Camins À, Naval-Baudin P, Majós C, Sierpowska J, Sanmillan JL, Cos M, Rodriguez-Fornells A, Gabarrós A.	J Neuroimaging	2022
**764**	A Diffusion Tensor Imaging Study on the White Matter Structures Related to the Phonology in Cantonese-Mandarin Bilinguals	Xu X, Jin Y, Pan N, Cao M, Jing J, Ma J, Fan X, Tan S, Song X, Li X.	Front Hum Neurosci	2022
**765**	Individual differences in white matter of the uncinate fasciculus and inferior fronto-occipital fasciculus: possible early biomarkers for callous-unemotional behaviors in young children with disruptive behavior problems	Graziano PA, Garic D, Dick AS.	J Child Psychol Psychiatry	2022
**766**	White Matter Correlates of Domain-Specific Working Memory	Horne A, Ding J, Schnur TT, Martin RC.	Brain Sci	2022
**767**	Impairment and Plasticity of Language-Related White Matter in Patients With Brain Arteriovenous Malformations	Deng X, Yin H, Zhang Y, Zhang D, Wang S, Cao Y, Li M, Wang B, Zong F, Zhao J.	Stroke	2022
**768**	Functional network and structural connections involved in picture naming	Jarret J, Ferré P, Chedid G, Bedetti C, Bore A, Joanette Y, Rouleau I, Maria Brambati S.	Brain Lang	2022
**769**	Segregated circuits for phonemic and	Zigiotto L, Vavassori L, Annicchiarico L, Corsini F, Avesani P, Rozzanigo U, Sarubbo S, Papagno C.	Neuroimage Clin	2022

	semantic fluency: A novel patient-tailored disconnection study			
**770**	Alice in Wonderland syndrome: a lesion mapping study	Piervincenzi C, Petsas N, Giannì C, Di Piero V, Pantano P.	Neurol Sci	2022
**771**	Exploring the ventral white matter language network in bimodal and unimodal bilinguals	Quartarone C, Navarrete E, Budisavljević S, Peressotti F.	Brain Lang	2022
**772**	Two different subcortical language networks supporting distinct Japanese orthographies: morphograms and phonograms	Tamai S, Kinoshita M, Nakajima R, Okita H, Nakada M.	Brain Struct Funct	2022
**773**	Mapping brain-wide excitatory projectome of primate prefrontal cortex at submicron resolution and comparison with diffusion tractography	Yan M, Yu W, Lv Q, Lv Q, Bo T, Chen X, Liu Y, Zhan Y, Yan S, Shen X, Yang B, Hu Q, Yu J, Qiu Z, Feng Y, Zhang XY, Wang H, Xu F, Wang Z.	Elife	2022
**774**	Correlation of Hemispatial Neglect with White Matter Tract Integrity: A DTI Study	Kwon BM, Lee JY, Ko N, Kim BR, Moon WJ, Choi DH, Lee J.	Brain Neurorehabil	2022
**775**	Early white matter connectivity and plasticity in post stroke aphasia recovery	Schevenels K, Gerrits R, Lemmens R, De Smedt B, Zink I, Vandermosten M.	Neuroimage Clin	2022
**776**	Track-weighted imaging analysis of white matter microstructures in healthy children: Sex and hemispheric differences	Raja R, Na X, Glasier C, Badger T, Akmyradov C, Ou X.	Proc SPIE Int Soc Opt Eng	2022
**777**	Brain microstructural antecedents of visual difficulties in infants born very preterm	Chandwani R, Harpster K, Kline JE, Mehta V, Wang H, Merhar SL, Schwartz TL, Parikh NA.	Neuroimage Clin	2022
**778**	Alterations in white matter microstructural properties after lingual strength exercise in patients with dysphagia	Krekeler BN, Hou J, Nair VA, Prabhakaran V, Rusche N, Rogus-Pulia N, Robbins J.	Neuroreport	2022
**779**	Comparison of Diffusion Signal Models for Fiber Tractography in Eloquent Glioma Surgery-Determination of Accuracy Under Awake Craniotomy Conditions	Becker D, Neher P, Jungk C, Jesser J, Pflüger I, Brinster R, Bendszus M, Bruckner T, Maier-Hein K, Scherer M, Unterberg A.	World Neurosurg	2022
**780**	Alterations in white matter microarchitecture in adolescents and young adults with major depressive disorder: A voxel-based meta-analysis of diffusion tensor imaging	Zhou L, Wang L, Wang M, Dai G, Xiao Y, Feng Z, Wang S, Chen G.	Psychiatry Res Neuroimaging	2022

**781**	Aberrant White Matter Microstructure in Depressed Patients with Suicidality	Zhang H, Li H, Yin L, Chen Z, Wu B, Huang X, Jia Z, Gong Q.	J Magn Reson Imaging	2022
**782**	A tract-based spatial statistics study of white matter integrity in epilepsy	Hou XX, Feng HX, Xu B, Li ZS, Lu YL, Zhao HM, Wang MX, Shi QR, Gui Q, Wu GH, Shen MQ, Zhu W, Xu QR, Dong XF, Cheng QZ, Zhang JB, Ding ZL.	Am J Transl Res	2022
**783**	Optic Tract as an Upper Limit in Amygdalectomy: Microsurgical Study	Garcia-Oriola G, Tomasi SO, Gallardo F, Umana GE, Lawton MT, Winkler PA.	Oper Neurosurg (Hagerstown)	2022
**784**	Jargonaphasia as a disconnection syndrome: A study combining white matter electrical stimulation and disconnectome mapping	Giampiccolo D, Moritz-Gasser S, Ng S, Lemaître AL, Duffau H.	Brain Stimul	2022
**785**	Association fiber tracts related to Broca's area: A comparative study based on diffusion spectrum imaging and fiber dissection	Wu Y, Liu J, Yu G, Jv R, Wang Y, Zang P.	Front Neurosci	2022
**786**	The long-range white matter microstructural alterations in drug-naive children with ADHD: A tract-based spatial statistics study	Zhou R, Dong P, Chen S, Qian A, Tao J, Zheng X, Cheng J, Yang C, Huang X, Wang M.	Psychiatry Res Neuroimaging	2022
**787**	Reduced myelin density in unmedicated major depressive disorder: An inhomogeneous magnetization transfer MRI study	Chen G, Fu S, Chen P, Zhong S, Chen F, Qian L, Luo Z, Pan Y, Tang G, Jia Y, Huang L, Wang Y.	J Affect Disord	2022
**788**	Accelerated decline in white matter microstructure in subsequently impaired older adults and its relationship with cognitive decline	Shafer AT, Williams OA, Perez E, An Y, Landman BA, Ferrucci L, Resnick SM.	Brain Commun	2022
**789**	Characteristics of Microstructural Changes Associated with Glioma Related Epilepsy: A Diffusion Tensor Imaging (DTI) Study	Zhang H, Zhou C, Zhu Q, Li T, Wang Y, Wang L.	Brain Sci	2022
**790**	Multimodal MRI cerebral correlates of verbal fluency switching and its impairment in women with depression	Domain L, Guillery M, Linz N, König A, Batail JM, David R, Corouge I, Bannier E, Ferré JC, Dondaine T, Drapier D, Robert GH.	Neuroimage Clin	2022
**791**	[A study on longitudinal changes in white matter microstructure of parents who have lost their only child based on diffusion tensor	Lan QY, Cao ZH, Qi RF, Luo YF, Zhang JY, Ge HH, Dai P, Liu F, Chen LJ, Li GM, Lu G.	Zhonghua Yi Xue Za Zhi	2022

	imaging and its relationship with symptoms of posttraumatic stress disorder]			
**792**	White matter tract-specific microstructural disruption is associated with depressive symptoms in isolated RBD	Byun JI, Oh S, Sunwoo JS, Shin JW, Kim TJ, Jun JS, Kim HJ, Shin WC, Seong JK, Jung KY.	Neuroimage Clin	2022
**793**	Effects of Arterial Stiffness on Cerebral WM Integrity in Older Adults: A Neurite Orientation Dispersion and Density Imaging and Magnetization Transfer Saturation Imaging Study	Kikuta J, Kamagata K, Abe M, Andica C, Saito Y, Takabayashi K, Uchida W, Naito H, Tabata H, Wada A, Tamura Y, Kawamori R, Watada H, Aoki S.	AJNR Am J Neuroradiol	2022
**794**	White Matter Microstructure and Gray Matter Volume in Cannabis-Induced Psychosis and Schizophrenia With Cannabis Use	Shah R, Ghosh A, Avasthi A, Ahuja CK, Khandelwal N, Nehra R.	J Neuropsychiatry Clin Neurosci	2022
**795**	Comparison of White Matter Structure of Drug-Naïve Patients With Bipolar Disorder and Major Depressive Disorder Using Diffusion Tensor Tractography	Koreki A, Niida R, Niida A, Yamagata B, Anamizu S, Mimura M.	Front Psychiatry	2022
**796**	Alterations in White Matter Fiber Tracts Characterized by Automated Fiber-Tract Quantification and Their Correlations With Cognitive Impairment in Neuromyelitis Optica Spectrum Disorder Patients	Yan Z, Wang X, Zhu Q, Shi Z, Chen X, Han Y, Zheng Q, Wei Y, Wang J, Li Y.	Front Neurosci	2022
**797**	Microsurgical anatomy of the auditory radiations: revealing the enigmatic acoustic pathway from a surgical viewpoint	Esen Aydin A, Aydin S, Bilgin B, Mirkhasilova M, Bayramli N, Tanriover N.	J Neurosurg	2022
**798**	Effect of the long-term lack of half visual inputs on the white matter microstructure in congenital monocular blindness	Qu X, Ding J, Wang Q, Cui J, Dong J, Guo J, Li T, Xie L, Li D, Xian J.	Brain Res	2022
**799**	A Prospective Study of the Impact of Severe Childhood Deprivation on Brain White Matter in Adult Adoptees: Widespread Localized Reductions in Volume	Mackes NK, Mehta MA, Beyh A, Nkrumah RO, Golm D, Sarkar S, Fairchild G, Dell'Acqua F, Sonuga-Barke EJS; ERA Young Adult Follow-Up Team.	eNeuro	2022

	But Unaffected Microstructural Organization			
**800**	Disrupted White Matter Integrity and Cognitive Functions in Amyloid-β Positive Alzheimer's Disease with Concomitant Lobar Cerebral Microbleeds	Qiao Y, Sun Y, Guo J, Chen Y, Hou W, Zhang J, Peng D.	J Alzheimers Dis	2022
**801**	Deep brain stimulation in Parkinson's disease: analysis of brain fractional anisotropy differences in operated patients	Arévalo-Sáenz A, López-Manzanares L, Navas-García M, Pastor J, Vega-Zelaya L, Torres CV.	Rev Neurol	2022
**802**	Tractography in Type 2 Diabetes Mellitus With Subjective Memory Complaints: A Diffusion Tensor Imaging Study	Wang J, Ma L, Liu G, Bai W, Ai K, Zhang P, Hu W, Zhang J.	Front Neurosci	2022
**803**	Cognitive and functional deficits are associated with white matter abnormalities in two independent cohorts of patients with schizophrenia	Yamada S, Takahashi S, Malchow B, Papazova I, Stöcklein S, Ertl-Wagner B, Papazov B, Kumpf U, Wobrock T, Keller-Varady K, Hasan A, Falkai P, Wagner E, Raabe FJ, Keeser D.	Eur Arch Psychiatry Clin Neurosci	2022
**804**	Comparing human and chimpanzee temporal lobe neuroanatomy reveals modifications to human language hubs beyond the frontotemporal arcuate fasciculus	Sierpowska J, Bryant KL, Janssen N, Blazquez Freches G, Römkens M, Mangnus M, Mars RB, Piai V.	Proc Natl Acad Sci U S A	2022
**805**	The effect of polygenic risk on white matter microstructural degeneration in Parkinson's disease: A longitudinal Diffusion Tensor Imaging study	Gu L, Guan X, Gao T, Zhou C, Yang W, Lv D, Wu J, Fang Y, Guo T, Song Z, Xu X, Tian J, Yin X, Zhang M, Zhang B, Pu J, Yan Y.	Eur J Neurol	2022
**806**	Associations between Brain Microstructure and Phonological Processing Ability in Preschool Children	Zhou Y, Li G, Song Z, Zhang Z, Huang H, Li H, Tang X.	Children (Basel)	2022
**807**	Microsurgical anatomy and insular connectivity of the cerebral opercula	Demirtaş OK, Güngör A, Çeltikçi P, Çeltikçi E, Munoz-Gualan AP, Doğulu FH, Türe U.	J Neurosurg	2022
**808**	Altered White Matter Microstructure in Herpes Zoster and Postherpetic Neuralgia Determined by Automated Fiber Quantification	Wu Y, Gu L, Hong S, Li J, Yang J, Xiong J, Lv H, Jiang J.	Brain Sci	2022
**809**	White matter alterations in focal to bilateral tonic-clonic seizures	Maher C, D'Souza A, Zeng R, Barnett M, Kavehei O, Nikpour A, Wang C.	Front Neurol	2022

**810**	Test-retest and repositioning effects of white matter microstructure measurements in selected white matter tracts	Anand C, Brandmaier AM, Lynn J, Arshad M, Stanley JA, Raz N.	Neuroimage Rep	2022
**811**	Sensory processing sensitivity and axonal microarchitecture: identifying brain structural characteristics for behavior	David S, Brown LL, Heemskerk AM, Aron E, Leemans A, Aron A.	Brain Struct Funct	2022
**812**	Association between Changes in White Matter Microstructure and Cognitive Impairment in White Matter Lesions	Hu AM, Ma YL, Li YX, Han ZZ, Yan N, Zhang YM.	Brain Sci	2022
**813**	Trajectories of brain white matter development in young children with prenatal alcohol exposure	Kar P, Reynolds JE, Gibbard WB, McMorris C, Tortorelli C, Lebel C.	Hum Brain Mapp	2022
**814**	Disruption of white matter integrity and its relationship with cognitive function in non-severe traumatic brain injury	Abdullah AN, Ahmad AH, Zakaria R, Tamam S, Abd Hamid AI, Chai WJ, Omar H, Abdul Rahman MR, Fitzrol DN, Idris Z, Ghani ARI, Wan Mohamad WNA, Mustafar F, Hanafi MH, Reza MF, Umar H, Mohd Zulkifly MF, Ang SY, Zakaria Z, Musa KI, Othman A, Embong Z, Sapiai NA, Kandasamy R, Ibrahim H, Abdullah MZ, Amaruchkul K, Valdes-Sosa PA, Bringas Vega ML, Biswal B, Songsiri J, Yaacob HS, Sumari P, Noh NA, Azman A, Jamir Singh PS, Abdullah JM.	Front Neurol	2022
**815**	Endoscopic Anatomy of Transcallosal Hemispherotomy: Laboratory Study with Advanced Three-Dimensional Modeling	Roldan P, Guizzardi G, Di Somma A, Valera R, Varriano F, Donaire A, Hoyos J, Topczewski TE, Torales J, Enseñat J, Rumia J, Prats-Galino A.	World Neurosurg	2022
**816**	Stroke disconnectome decodes reading networks	Forkel SJ, Labache L, Nachev P, Thiebaut de Schotten M, Hesling I.	Brain Struct Funct	2022
**817**	Cerebrospinal Fluid sTREM2 Has Paradoxical Association with Brain Structural Damage Rate in Early- and Late-Stage Alzheimer's Disease	Leng F, Zhan Z, Sun Y, Liu F, Edison P, Sun Y, Wang Z; Alzheimer's Disease Neuroimaging Initiative.	J Alzheimers Dis	2022
**818**	Characteristics of White Matter Integrity During Different Phases of Abstinence in Heroin Use Disorders: A Diffusion Tensor Imaging Study	Zhu J, Yan X, Lyu Z, Wang S, Chen J, Wang W, Li Q, Li W.	J Addict Med	2022
**819**	White matter correlates of sensorimotor synchronization in persistent developmental stuttering	Jossinger S, Sares A, Zislis A, Sury D, Gracco V, Ben-Shachar M.	J Commun Disord	2022

**820**	Developmental pattern of association fibers and their interaction with associated cortical microstructures in 0-5-month-old infants	Liu T, Wu J, Zhao Z, Li M, Lv Y, Li M, Gao F, You Y, Zhang H, Ji C, Wu D.	Neuroimage	2022
**821**	White matter pathways associated with empathy in females: A DTI investigation	Steinberg SN, Tedla NB, Hecht E, Robins DL, King TZ.	Brain Cogn	2022
**822**	Individual variation in white matter microstructure is related to better recovery from negative stimuli	Pedersen WS, Dean DC, Adluru N, Gresham LK, Lee SD, Kelly MP, Mumford JA, Davidson RJ, Schaefer SM.	Emotion	2022
**823**	Impaired white matter microstructure associated with severe depressive symptoms in patients with PD	Shen Q, Liu Y, Guo J, Zhang H, Xiang Y, Liao H, Cai S, Zhou B, Wang M, Liu S, Yi J, Zhang Z, Tan C.	Brain Imaging Behav	2022
**824**	Brain microstructural changes and fatigue after COVID-19	Bispo DDC, Brandão PRP, Pereira DA, Maluf FB, Dias BA, Paranhos HR, von Glehn F, de Oliveira ACP, Regattieri NAT, Silva LS, Yasuda CL, Soares AASM, Descoteaux M.	Front Neurol	2022
**825**	White matter integrity and key structures affected in Alzheimer's disease characterized by diffusion tensor imaging	Xiao D, Wang K, Theriault L, Charbel E; Alzheimer's Disease Neuroimaging Initiative.	Eur J Neurosci	2022
**826**	Neural underpinnings of the slowness of information processing in patients with traumatic brain injury: insights from tract-based spatial statistics	Boccia M, Barbetti S, Valentini F, De Angelis C, Tanzilli A, Fabio V, Guariglia C, Galati G, Formisano R, Ciurli MP.	Neurol Sci	2022
**827**	Social Health Is Associated With Tract-Specific Brain White Matter Microstructure in Community-Dwelling Older Adults	Costanzo A, van der Velpen IF, Ikram MA, Vernooij-Dassen MJF, Niessen WJ, Vernooij MW, Kas MJ.	Biol Psychiatry Glob Open Sci	2022
**828**	Left parietal involvement in motion sickness susceptibility revealed by multimodal magnetic resonance imaging	Sakai H, Harada T, Larroque SK, Demertzi A, Sugawara T, Ito T, Wada Y, Fukunaga M, Sadato N, Laureys S.	Hum Brain Mapp	2022
**829**	Treatment-resistant schizophrenia: Addressing white matter integrity, intracortical glutamate levels, clinical and cognitive profiles between early- and adult-onset patients	Matrone M, Kotzalidis GD, Romano A, Bozzao A, Cuomo I, Valente F, Gabaglio C, Lombardozzi G, Trovini G, Amici E, Perrini F, De Persis S, Iasevoli F, De Filippis S, de Bartolomeis A.	Prog Neuropsychopharmacol Biol Psychiatry	2022
**830**	Quantitative MRI Evidence for Cognitive Reserve in Healthy	Fingerhut H, Gozdas E, Hosseini SMH.	J Alzheimers Dis	2022

	Elders and Prodromal Alzheimer's Disease			
**831**	Identification of association fibers using ex vivo diffusion tractography in Alexander disease brains	Shiohama T, Stewart N, Nangaku M, van der Kouwe AJW, Takahashi E.	J Neuroimaging	2022
**832**	Right ventral stream damage underlies both poststroke aprosodia and amusia	Sihvonen AJ, Sammler D, Ripollés P, Leo V, Rodríguez-Fornells A, Soinila S, Särkämö T.	Eur J Neurol	2022
**833**	Preservation of frontal white matter tracts in ventricular surgery: favoring an anterior interhemispheric transcallosal approach vs a transcortical transfrontal transventricular approach	El-Bendary Y, Apra C, Aldea S, Chauvet D, Dorfmüller G, Ferrand-Sorbets S, Lecler A, Le Guérinel C, Bourdillon P.	Neurosurg Rev	2022
**834**	Selective association of cytokine levels and kynurenine/tryptophan ratio with alterations in white matter microstructure in bipolar but not in unipolar depression	Comai S, Melloni E, Lorenzi C, Bollettini I, Vai B, Zanardi R, Colombo C, Valtorta F, Benedetti F, Poletti S.	Eur Neuropsychopharmacol	2022
**835**	White matter fiber-specific degeneration in older adults with metabolic syndrome	Andica C, Kamagata K, Uchida W, Takabayashi K, Shimoji K, Kaga H, Someya Y, Tamura Y, Kawamori R, Watada H, Hori M, Aoki S.	Mol Metab	2022
**836**	Characteristics of Brain White Matter Microstructure in HIV Male Patients With Primary Syphilis Co-Infection	Qi Y, Li RL, Wang YY, Wang W, Liu XZ, Liu J, Li X, Zhang XD, Yu W, Liu JJ, Guo YF, Rao B, Li HJ.	Front Neurol	2022
**837**	Brain grey and white matter structural associations with future suicidal ideation and behaviors in adolescent and young adult females with mood disorders	Colic L, Villa LM, Dauvermann MR, van Velzen LS, Sankar A, Goldman DA, Panchal P, Kim JA, Quatrano S, Spencer L, Constable RT, Suckling J, Goodyer IM, Schmaal L, van Harmelen AL, Blumberg HP.	JCPP Adv	2022
**838**	Tract-based white matter hyperintensity patterns in patients with systemic lupus erythematosus using an unsupervised machine learning approach	Rumetshofer T, Inglese F, de Bresser J, Mannfolk P, Strandberg O, Jönsen A, Bengtsson A, Nilsson M, Knutsson L, Lätt J, Steup-Beekman GM, Huizinga TWJ, van Buchem MA, Ronen I, Sundgren PC.	Sci Rep	2022
**839**	Early life factors and white matter microstructure in children with overweight and obesity: The ActiveBrains project	Solis-Urra P, Esteban-Cornejo I, Rodriguez-Ayllon M, Verdejo-Román J, Labayen I, Catena A, Ortega FB.	Clin Nutr	2022
**840**	Structural connectivity in ventral language pathways characterizes non-verbal autism	Olivé G, Slušná D, Vaquero L, Muchart-López J, Rodríguez-Fornells A, Hinzen W.	Brain Struct Funct	2022

**841**	The importance of basal-temporal white matter to pre- and post-surgical naming ability in temporal lobe epilepsy	Kaestner E, Stasenko A, Ben-Haim S, Shih J, Paul BM, McDonald CR.	Neuroimage Clin	2022
**842**	Abnormal White Matter Microstructure in the Limbic System Is Associated With Tuberous Sclerosis Complex-Associated Neuropsychiatric Disorders	Sato A, Tominaga K, Iwatani Y, Kato Y, Wataya-Kaneda M, Makita K, Nemoto K, Taniike M, Kagitani-Shimono K.	Front Neurol	2022
**843**	Alterations in the inferior fronto-occipital fasciculus - a specific neural correlate of gender incongruence?	van Heesewijk J, Steenwijk MD, Kreukels BPC, Veltman DJ, Bakker J, Burke SM.	Psychol Med	2023
**844**	White and gray matter alterations in de novo PD patients: which matter most?	Pietracupa S, Belvisi D, Piervincenzi C, Tommasin S, Pasqua G, Petsas N, De Bartolo MI, Fabbrini A, Costanzo M, Manzo N, Berardelli A, Pantano P.	J Neurol	2023
**845**	Dissociations in white matter tracts and neuropsychological findings in a 17 years old patient with Subacute sclerosing panencephalitis	Tomasino B, Valente M, Negro ID, De Colle MC, Guarracino I, Maieron M, Gigli GL.	Brain Cogn	2023
**846**	Visual networks: Electric brain stimulation and diffusion tensor imaging	Tamada T, Enatsu R, Saito T, Chiba R, Kanno A, Mikuni N.	Rev Neurol (Paris)	2023
**847**	Impaired attention mechanisms in confabulating patients: A VLSM and DWI study	Colás-Blanco I, Chica AB, Thiebaut de Schotten M, Busquier H, Olivares G, Triviño M.	Cortex	2023
**848**	Micro-structural white matter abnormalities in new daily persistent headache: a DTI study using TBSS analysis	Mei Y, Wang W, Qiu D, Yuan Z, Bai X, Tang H, Zhang P, Zhang X, Zhang Y, Yu X, Sui B, Wang Y.	J Headache Pain	2023
**849**	Higher-order sensorimotor circuit of the whole-brain functional network involved in pruritus regulation in atopic dermatitis	Wu H, Dai W, Hong Z, Qin Y, Yang M, Wang B, Liao J.	J Eur Acad Dermatol Venereol	2023
**850**	A Consideration of Optimal Head Position in Transsylvian Selective Amygdalohippocampectomy	Hamasaki T, Uchikawa H, Kawano T, Kai K, Takezaki T, Mukasa A.	Neurol Med Chir (Tokyo)	2023
**851**	Functional and anatomical alterations in bilateral vestibulopathy: A multimodal neuroimaging study and clinical correlation	Lee ES, Weon YC, Kim JS, Lee TK, Park JY.	Front Neurol	2023

**852**	Microstructural Alterations in Projection and Association Fibers in Neonatal Hypoxia-Ischemia	Cao Z, Lin H, Gao F, Shen X, Zhang H, Zhang J, Du L, Lai C, Ma X, Wu D.	J Magn Reson Imaging	2023
**853**	Development of associational fiber tracts in fetal human brain: a cadaveric laboratory investigation	Di Carlo DT, Filice ME, Fava A, Quilici F, Fuochi B, Cecchi P, Donatelli G, Restani L, Nardini V, Turillazzi E, Cosottini M, Perrini P.	Brain Struct Funct	2023
**854**	Neural correlates of mental state decoding and mental state reasoning in schizophrenia	Demirlek C, Karakılıç M, Sarıkaya E, Bayrakçı A, Verim B, Gülyüksel F, Yalınçetin B, Oral E, Gelal F, Zorlu N, Bora E.	Psychiatry Res Neuroimaging	2023
**855**	Preoperative language tract integrity is a limiting factor in recovery from aphasia after glioma surgery	Prasse G, Meyer HJ, Scherlach C, Maybaum J, Hoffmann A, Kasper J, Karl Fehrenbach M, Wilhelmy F, Meixensberger J, Hoffmann KT, Wende T.	Neuroimage Clin	2023
**856**	White matter microstructure is associated with the precision of visual working memory	Li X, Rangelov D, Mattingley JB, Oestreich L, Lévy-Bencheton D, O'Sullivan MJ.	Neuroimage	2023
**857**	Contribution of White Matter Fiber Bundle Damage to Language Change After Surgery for Temporal Lobe Epilepsy	Binding LP, Dasgupta D, Taylor PN, Thompson PJ, O'Keeffe AG, de Tisi J, McEvoy AW, Miserocchi A, Winston GP, Duncan JS, Vos SB.	Neurology	2023
**858**	Magnetic Resonance Tractography and Intraoperative Direct Electrical Stimulation in Eloquent Area Glioma Surgery for 102 Cases: A Tertiary Care Center Experience From Northwest India	Sahoo SK, Mohanty M, Emanee SYS, Prabhakar A, Panda N, Chauhan R, Soni SL, Gendle C, Kumar A.	World Neurosurg	2023
**859**	An Applied Anatomic Guide to Anterior Temporal Lobectomy and Amygdalohippocampectomy: Laboratory Cranial and White Matter Dissections to Inform Surgical Practice	Charalampopoulou E, Neromyliotis E, Anastasopoulos L, Komaitis S, Drosos E, Skandalakis GP, Kalyvas AV, Stranjalis G, Koutsarnakis C.	Oper Neurosurg (Hagerstown)	2023
**860**	Clinical and cognitive correlates tractography analysis in patients with white matter hyperintensity of vascular origin	Kuang Q, Huang M, Lei Y, Wu L, Jin C, Dai J, Zhou F.	Front Neurosci	2023
**861**	Differences in structural MRI and diffusion tensor imaging underlie visuomotor performance declines in older adults with an	Rogojin A, Gorbet DJ, Hawkins KM, Sergio LE.	Front Aging Neurosci	2023

	increased risk for Alzheimer's disease			
**862**	White matter changes and its relationship with clinical symptom in medication-naive first-episode early onset schizophrenia	Cai J, Xie M, Zhao L, Li X, Liang S, Deng W, Guo W, Ma X, Sham PC, Wang Q, Li T.	Asian J Psychiatr	2023
**863**	Atypical development in white matter microstructures in ADHD: A longitudinal diffusion imaging study	Chiang HL, Tseng WI, Tseng WL, Tung YH, Hsu YC, Chen CL, Gau SS.	Asian J Psychiatr	2023
**864**	Characterisation of brain microstructural alterations in children with obstructive sleep apnea syndrome using diffusion kurtosis imaging	Li Y, Wen H, Li H, Peng Y, Tai J, Bai J, Mei L, Ji T, Li X, Liu Y, Ni X.	J Sleep Res	2023
**865**	Improved prediction of glioma-related aphasia by diffusion MRI metrics, machine learning, and automated fiber bundle segmentation	Shams B, Reisch K, Vajkoczy P, Lippert C, Picht T, Fekonja LS.	Hum Brain Mapp	2023
**866**	Abnormal white matter along fibers by automated fiber quantification in patients undergoing hemodialysis	Chen HJ, Qiu J, Xu X, Guo Y, Fu L, Fu Q, Wu Y, Qi Y, Chen F.	Neurol Sci	2023
**867**	Disrupted structural connectivity and less efficient network system in patients with the treatment-naive adult attention-deficit/hyperactivity disorder	Ohnishi T, Toda W, Itagaki S, Sato A, Matsumoto J, Ito H, Ishii S, Miura I, Yabe H.	Front Psychiatry	2023
**868**	Diffusion Tensor Imaging of Auditory Pathway: A Comparison of Pediatric Cochlear Implant Candidates and Healthy Cases	Aksoy DÖ, Karagöz Y, Kaldırımoğlu KF, Ulusan MB, Mahmutoğlu AS.	J Int Adv Otol	2023
**869**	Automated Tractography for the Assessment of Aphasia in Acute Care Stroke Rehabilitation: A Case Series	Mochizuki M, Uchiyama Y, Domen K, Koyama T.	Prog Rehabil Med	2023
**870**	Segmental disturbance of white matter microstructure in predicting mild cognitive impairment in idiopathic Parkinson's disease: An individualized study based on automated fiber quantification tractography	Yu Z, Pang H, Yu H, Wu Z, Ding Z, Fan G.	Parkinsonism Relat Disord	2023

**871**	White matter microstructure alterations in idiopathic restless legs syndrome: a study combining crossing fiber-based and tensor-based approaches	Xue Y, Xie S, Wang X, Xi X, Liu C.	Front Neurosci	2023
**872**	Neural substrates of interoceptive sensibility: An integrated study in normal and pathological functioning	Boccia M, Teghil A, Raimo S, Di Vita A, Grossi D, Guariglia C, Palermo L.	Neuropsychologia	2023
**873**	Persistence of post-traumatic stress disorder in Chinese Shidu parents is associated with combined gray and white matter abnormalities	Ge J, Luo Y, Qi R, Wu L, Dai H, Lan Q, Liu B, Zhang L, Lu G, Cao Z, Shen J.	Psychiatry Res Neuroimaging	2023
**874**	Right hemisphere and metaphor comprehension: A connectionist perspective	Duque ACM, Cuesta TAC, Melo AS, Maldonado IL.	Neuropsychologia	2023
**875**	Robust associations between white matter microstructure and general intelligence	Stammen C, Fraenz C, Grazioplene RG, Schlüter C, Merhof V, Johnson W, Güntürkün O, DeYoung CG, Genç E.	Cereb Cortex	2023
**876**	Secondary neurodegeneration following Stroke: what can blood biomarkers tell us?	Brunelli S, Giannella E, Bizzaglia M, De Angelis D, Sancesario GM.	Front Neurol	2023
**877**	Functional MRI but not white matter fibre dissection identifies language dominance	Al Busaidi A, Gangemi E, Wastling S, Berg ASVD, Mancini L, Yousry T.	Eur Radiol	2023
**878**	Diffusion-Weighted Magnetic Resonance Imaging Demonstrates White Matter Alterations in Watershed Regions in Children With Moyamoya Without Stroke or Silent Infarct	Ahtam B, Solti M, Doo JM, Feldman HA, Vyas R, Zhang F, O'Donnell LJ, Rathi Y, Smith ER, Orbach D, See AP, Grant PE, Lehman LL.	Pediatr Neurol	2023
**879**	Sex disparity of cerebral white matter hyperintensity in the hypertensive elderly: The Shanghai Changfeng study	Wang L, Lin H, Zhao Z, Chen L, Wu L, Liu T, Li J, Huang CC, Peng Y, Lo CZ, Gao X.	Hum Brain Mapp	2023
**880**	Lesion-symptom mapping of language impairments in people with brain tumours: The influence of linguistic stimuli	Ntemou E, Rybka L, Lubbers J, Tuncer MS, Vajkoczy P, Rofes A, Picht T, Faust K.	J Neuropsychol	2023
**881**	Characteristics of white matter alterations along fibres in patients with bulimia	Chen Q, Wang M, Wu GW, Li WH, Ren XD, Wang YL, Wei X, Wang JN, Yang Z, Li XH, Li ZJ, Tang LR, Zhang P, Wang Z.	Eur J Neurosci	2023

	nervosa: A combined voxelwise and tractography study			
**882**	Multiparametric mapping of white matter reorganizations in patients with frontal glioma-related epilepsy	Zhang S, Zhao F, Yang X, Tan Q, Li S, Shao H, Su X, Gong Q, Yue Q.	CNS Neurosci Ther	2023
**883**	White matter alterations in MR-negative temporal and frontal lobe epilepsy using fixel-based analysis	Bartoňová M, Tournier JD, Bartoň M, Říha P, Vojtíšek L, Mareček R, Doležalová I, Rektor I.	Sci Rep	2023
**884**	Diffusivity Changes in Posterior Cortical Atrophy and Logopenic Progressive Aphasia: A Longitudinal Diffusion Tensor Imaging Study	Singh NA, Graff-Radford J, Machulda MM, Pham NTT, Schwarz CG, Reid RI, Lowe VJ, Petersen RC, Jack CR, Josephs KA, Whitwell JL.	J Alzheimers Dis	2023
**885**	Altered white matter integrity in euthymic children with bipolar disorder: A tract-based spatial statistical analysis of diffusion tensor imaging	Singh A, Pandey HR, Arya A, Agarwal V, Shree R, Kumar U.	J Affect Disord	2023
**886**	Negative emotion differentiation and white matter microstructure	Matyi MA, Spielberg JM.	J Affect Disord	2023
**887**	Dynamic cortical and tractography atlases of proactive and reactive alpha and high-gamma activities	Ono H, Sonoda M, Sakakura K, Kitazawa Y, Mitsuhashi T, Firestone E, Jeong JW, Luat AF, Marupudi NI, Sood S, Asano E.	Brain Commun	2023
**888**	Study of the microstructure of brain white matter in medial temporal lobe epilepsy based on diffusion tensor imaging	Zhang Y, Liu Z, Dou W, Wei J, Lv Y, Hou B, You H, Feng F.	Brain Behav	2023
**889**	Study of the relationship between onset lateralization and hemispheric white matter asymmetry in Parkinson's disease	Zhu Y, Li S, Da X, Lai H, Tan C, Liu X, Deng F, Chen L.	J Neurol	2023
**890**	Intra- and inter-hemispheric network dynamics supporting object recognition and speech production	Kitazawa Y, Sonoda M, Sakakura K, Mitsuhashi T, Firestone E, Ueda R, Kambara T, Iwaki H, Luat AF, Marupudi NI, Sood S, Asano E.	Neuroimage	2023
**891**	White matter microstructural abnormalities in individuals with attenuated positive symptom syndromes	Chen Z, Bo Q, Zhao L, Wang Y, Zhang Z, Zhou Y, Wang C.	J Psychiatr Res	2023
**892**	Joint impact on attention, alertness and inhibition of lesions at a frontal white matter crossroad	Kaufmann BC, Cazzoli D, Pastore-Wapp M, Vanbellingen T, Pflugshaupt T, Bauer D, Müri RM, Nef T, Bartolomeo P, Nyffeler T.	Brain	2023

**893**	Exploring structural and functional alterations in drug-naïve obsessive-compulsive disorder patients: An ultrahigh field multimodal MRI study	Tang W, Shen T, Huang Y, Zhu W, You S, Zhu C, Zhang L, Ma J, Wang Y, Zhao J, Li T, Lai HY.	Asian J Psychiatr	2023
**894**	DTI of Opioid-Exposed Fetuses Using ComBat Harmonization: A Bi-Institutional Study	Dudley JA, Nagaraj UD, Merhar S, Mangano FT, Kline-Fath BM, Ou X, Acheson A, Yuan W.	AJNR Am J Neuroradiol	2023
**895**	Familial risk for major depression: differential white matter alterations in healthy and depressed participants	Winter A, Thiel K, Meinert S, Lemke H, Waltemate L, Breuer F, Culemann R, Pfarr JK, Stein F, Brosch K, Meller T, Ringwald KG, Thomas-Odenthal F, Jansen A, Nenadić I, Krug A, Repple J, Opel N, Dohm K, Leehr EJ, Grotegerd D, Kugel H, Hahn T, Kircher T, Dannlowski U.	Psychol Med	2023
**896**	Causal relationships between migraine and microstructural white matter: a Mendelian randomization study	Zhao L, Zhao W; International Headache Genetics Consortium (IHGC); Cao J, Tu Y.	J Headache Pain	2023
**897**	Strategic white matter hyperintensity locations for cognitive impairment: A multicenter lesion-symptom mapping study in 3525 memory clinic patients	Coenen M, Kuijf HJ, Huenges Wajer IMC, Duering M, Wolters FJ, Fletcher EF, Maillard PM; Alzheimer's Disease Neuroimaging Initiative; Barkhof F, Barnes J, Benke T, Boomsma JMF, Chen CPLH, Dal-Bianco P, Dewenter A, Enzinger C, Ewers M, Exalto LG, Franzmeier N, Groeneveld O, Hilal S, Hofer E, Koek DL, Maier AB, McCreary CR, Padilla CS, Papma JM, Paterson RW, Pijnenburg YAL, Rubinski A, Schmidt R, Schott JM, Slattery CF, Smith EE, Steketee RME, Sudre CH, van den Berg E, van der Flier WM, Venketasubramanian N, Vernooij MW, Xin X, DeCarli C, Biessels GJ, Biesbroek JM.	Alzheimers Dement	2023
**898**	Asymmetry of radiomics features in the white matter of patients with primary progressive aphasia	Tafuri B, Filardi M, Urso D, Gnoni V, De Blasi R, Nigro S, Logroscino G; Frontotemporal Lobar Degeneration Neuroimaging Initiative.	Front Aging Neurosci	2023
**899**	White matter tracts contribute selectively to cognitive functioning in patients with glioma	Andreoli M, Mackie MA, Aaby D, Tate MC.	Front Oncol	2023
**900**	White matter integrity mediates the associations between white matter hyperintensities and cognitive function in patients with silent cerebrovascular diseases	Chen J, Ge A, Zhou Y, Ma Y, Zhong S, Chen C, Shi W, Ding J, Wang X.	CNS Neurosci Ther	2023
**901**	The connectivity-based parcellation of the angular gyrus: fiber dissection and MR tractography study	Yakar F, Çeltikçi P, Doğruel Y, Egemen E, Güngör A.	Brain Struct Funct	2023
**902**	Deficits in naming pictures of objects are associated with glioma infiltration of the inferior longitudinal fasciculus: A study with	Papagno C, Pascuzzo R, Ferrante C, Casarotti A, Riva M, Antelmi L, Gennari A, Mattavelli G, Bizzi A.	Hum Brain Mapp	2023

	diffusion MRI tractography, volumetric MRI, and neuropsychology			
**903**	Microsurgical anatomy of the isthmic cingulum: a new white matter crossroad and neurosurgical implications in the posteromedial interhemispheric approaches and the glioma invasion patterns	Saygi T, Avyasov R, Barut O, Daglar Z, Baran O, Hasimoglu O, Altinkaya A, Tanriover N.	Neurosurg Rev	2023
**904**	The inferior fronto-occipital fasciculus correlates with early precursors of mathematics and reading before the start of formal schooling	Vandecruys F, Vandermosten M, De Smedt B.	Cortex	2024
**905**	Educational stereoscopic representation of a step-by-step brain white fiber dissection according to Klingler's method	Jacquesson T, Djarouf I, Simon É, Haegelen C, Mertens P, Picart T, Fernandez-Miranda J.	Surg Radiol Anat	2024
**906**	Based on white matter microstructure to early identify bipolar disorder from patients with depressive episode	Sun H, Yan R, Hua L, Xia Y, Huang Y, Wang X, Yao Z, Lu Q.	J Affect Disord	2024
**907**	Alteration of white matter microstructure in patients with sleep disorders after COVID-19 infection	Qin H, Duan G, Zhou K, Qin L, Lai Y, Liu Y, Lu Y, Peng B, Zhang Y, Zhou X, Huang J, Huang J, Liang L, Wei Y, Zhang Q, Li X, OuYang Y, Bin B, Zhao M, Yang J, Deng D.	Sleep Med	2024
**908**	Alterations in the white matter structure of major depressive disorder patients and their link to childhood trauma	Zhao H, Rong B, Gao G, Zhou M, Huang J, Tu N, Bu L, Xiao L, Wang G.	Front Psychiatry	2024
**909**	Aberrant White Matter Development in Cerebral Visual Impairment: A Proposed Mechanism for Visual Dysfunction Following Early Brain Injury	Bauer CM, Merabet LB.	J Integr Neurosci	2024
**910**	Evidence of Ongoing Cerebral Microstructural Reorganization in Children With Persisting Symptoms Following Mild Traumatic Brain Injury: A NODDI DTI Analysis	Stein A, Vinh To X, Nasrallah FA, Barlow KM.	J Neurotrauma	2024

**911**	Exploring MGMT methylation-driven structural connectivity changes in insular gliomas: a tractography and graph theoretical analysis	Yang ZC, Yin CD, Yeh FC, Xue BW, Song XY, Li G, Sun SJ, Deng ZH, Hou ZG, Xie J.	J Neurooncol	2024
**912**	A normative modeling approach to quantify white matter changes and predict functional outcomes in stroke patients	Su H, Yan S, Zhu H, Liu Y, Zhang G, Peng X, Zhang S, Li Y, Zhu W.	Front Neurosci	2024
**913**	Brain white matter microstructural alterations in patients with systemic lupus erythematosus: an automated fiber quantification study	Zhang P, Feng Y, Xu T, Li Y, Xia J, Zhang H, Sun Z, Tian W, Zhang J.	Brain Imaging Behav	2024
**914**	White matter volume and treatment with selective progesterone receptor modulator in patients with premenstrual dysphoric disorder	Kaltsouni E, Wikström J, Lanzenberger R, Sundström-Poromaa I, Comasco E.	Psychoneuroendocrinology	2024
**915**	Q-ball high-resolution fiber tractography of language associated tracts: quantitative evaluation of applicability for glioma resections	Becker D, Scherer M, Neher P, Jungk C, Jesser J, Pflüger I, Bendszus M, Maier-Hein K, Unterberg A.	J Neurosurg Sci	2024
**916**	Impact of white matter hyperintensities on structural connectivity and cognition in cognitively intact ADNI participants	Taghvaei M, Mechanic-Hamilton DJ, Sadaghiani S, Shakibajahromi B, Dolui S, Das S, Brown C, Tackett W, Khandelwal P, Cook P, Shinohara RT, Yushkevich P, Bassett DS, Wolk DA, Detre JA; Alzheimer's Disease Neuroimaging Initiative.	Neurobiol Aging	2024
**917**	Causal relationship between multiparameter brain MRI phenotypes and age: evidence from Mendelian randomization	Wang X, Chen Q, Liu Y, Sun J, Li J, Zhao P, Cai L, Liu W, Yang Z, Wang Z, Lv H.	Brain Commun	2024
**918**	The relationship between blast-related mild traumatic brain injury and executive function is moderated by white matter integrity	O'Brien MC, Disner SG, Davenport ND, Sponheim SR.	Brain Imaging Behav	2024
**919**	White matter alterations in affective and non-affective early psychosis: A diffusion MRI study	Moghaddam HS, Parsaei M, Taghavizanjani F, Cattarinussi G, Aarabi MH, Sambataro F.	J Affect Disord	2024
**920**	Reproducibility of Diffusion MRI-Based Tractography in the Fetal Brain	Xiao J, Sun C, Chen R, Zhao Z, Wang G, Wu D.	J Magn Reson Imaging	2024
**921**	Free-water diffusion magnetic resonance imaging under	Takeshige-Amano H, Hatano T, Kamagata K, Andica C, Ogawa T, Shindo A, Uchida W, Sako	J Neurol Sci	2024

	selegiline treatment in Parkinson's disease	W, Saiki S, Shimo Y, Oyama G, Umemura A, Ito M, Hori M, Aoki S, Hattori N.		
**922**	Diffusion tensor imaging reveals distributed white matter abnormalities in primary trigeminal neuralgia: Tract-based spatial statistics study	Filimonova E, Pashkov A, Moysak G, Martirosyan A, Zaitsev B, Rzaev J.	Clin Neurol Neurosurg	2024
**923**	Visual outcomes and their association with grey and white matter microstructure in adults born preterm with very low birth weight	Ingvaldsen SH, Jørgensen AP, Grøtting A, Sand T, Eikenes L, Håberg AK, Indredavik MS, Lydersen S, Austeng D, Morken TS, Evensen KAI.	Sci Rep	2024
**924**	Distinguishing schizophrenia and bipolar disorder through a Multiclass Classification model based on multimodal neuroimaging data	Chen M, Xia X, Kang Z, Li Z, Dai J, Wu J, Chen C, Qiu Y, Liu T, Liu Y, Zhang Z, Shen Q, Tao S, Deng Z, Lin Y, Wei Q.	J Psychiatr Res	2024
**925**	Prenatal exposure to maternal disadvantage-related inflammatory biomarkers: associations with neonatal white matter microstructure	Sanders AFP, Tirado B, Seider NA, Triplett RL, Lean RE, Neil JJ, Miller JP, Tillman R, Smyser TA, Barch DM, Luby JL, Rogers CE, Smyser CD, Warner BB, Chen E, Miller GE.	Transl Psychiatry	2024
**926**	Ventral and dorsal aspects of the inferior frontal-occipital fasciculus support verbal semantic access and visually-guided behavioural control	Gonzalez Alam TRJ, Cruz Arias J, Jefferies E, Smallwood J, Leemans A, Marino Davolos J.	Brain Struct Funct	2024
**927**	Higher order neurocognition in pediatric brain tumor survivors: What can we learn from white matter microstructure?	Glazer S, Kim YJ, Fecher M, Billetdeaux KA, Gilliland EB, Wilde EA, Olshefski R, Yeates KO, Vannatta K, Hoskinson KR.	Pediatr Blood Cancer	2024
**928**	Baseline symptom-related white matter tracts predict individualized treatment response to 12-week antipsychotic monotherapies in first-episode schizophrenia	Chen Y, Liu S, Zhang B, Zhao G, Zhang Z, Li S, Li H, Yu X, Deng H, Cao H.	Transl Psychiatry	2024

Human structural MRI acquisition

A T1-weighted acquisition (single slab MPRAGE) was collected in a pure coronal orientation with a GE 3T Scanner. Field of view (FoV) was 24.0 x 18.0 x 17.2 cm, acquisition matrix was512 x 384 x 184 matrix size (AP x LR x IS), using 75% FoV in the ky-direction. Data were reconstructed to 512x512x368 with a voxel size of 0.47x0.47x0.40 mm. TR 15.8ms, TE 5.5ms,

flip angle 9°, inversion time 200 ms, number of averages 2, receiver bandwidth ±15.63kHz (corresponding to 59.98Hz/pixel on non-GE scanners).

Diffusion MRI processing

Human diffusion MRI comprised a multi-shell acquisition (1.6 mm isotropic resolution, 101 directions, 14 b0, *b*-values: 300, 700, and 2500 s/mm2) in a GE 3T Scanner. Diffusion MRI data were corrected for noise, Gibbs ringing and signal drift using MRtrix3 (https://www.mrtrix.org).[1] Distortion correction was performed using a synthesized b0 (Synb0-DisCo) produced from a T1-weighted MRI.[2] The result was then included into FSL's *Topup*. Magnetic susceptibility field, Eddy current and motion artifact correction were performed using FSL (https://fsl.fmrib.ox.ac.uk/fsl).[3] Response functions for cerebrospinal fluid, and white and grey matter were estimated using Single-Shell 3-Tissue CSD and Multi-Shell 3-Tissue CSD in MRtrix 3. Anatomically constrained tractography (ACT) using hybrid surface and volume segmentation in MRtrix3 was performed using second-order integration over fiber orientation distribution probabilistic fiber tracking algorithm selecting a maximum of 5000 streamlines from 30 million seeds.[4] Cortical parcellation for FreeSurfer were used for probabilistic seed-based constrained spherical deconvolution tractography in MRtrix3 (https://www.mrtrix.org).

For primate tractography, preprocessing was performed with ExploreDTI and MRTrix, correcting for noise, Gibbs ringing, motion artefact and Eddy. Whole brain probabilistic high angular resolution diffusion imaging was computed using StarTrack (https://www.mr-startrack.com/) for marmoset and macaques. Spherical deconvolution was based on a modified damped Richardson-Lucy algorithm[5] with the following parameters: fibre response $\alpha = 1.5$; number of iterations = 300; amplitude threshold $\eta = 0.0015$; geometric regularisation $v = 16$. Fibre tracking was performed according to the following parameters: minimum HMOA threshold = 0.003; number of seeds per voxel = 10; maximum angle threshold = 25°; minimum fibre length = 20 mm; maximum fibre length = 300 mm. Multishell data (marmoset) were processed using the shell with the highest b-value in StarTrack. Squirrel monkey were already processed as detailed in http://saimiri.bcblab.com.[6] Chimpanzee data was processed using a second-order integration over fiber orientation distribution probabilistic fiber tracking algorithm selecting a maximum of 5000 streamlines from 30 million seeds in MRtrix3.[4]

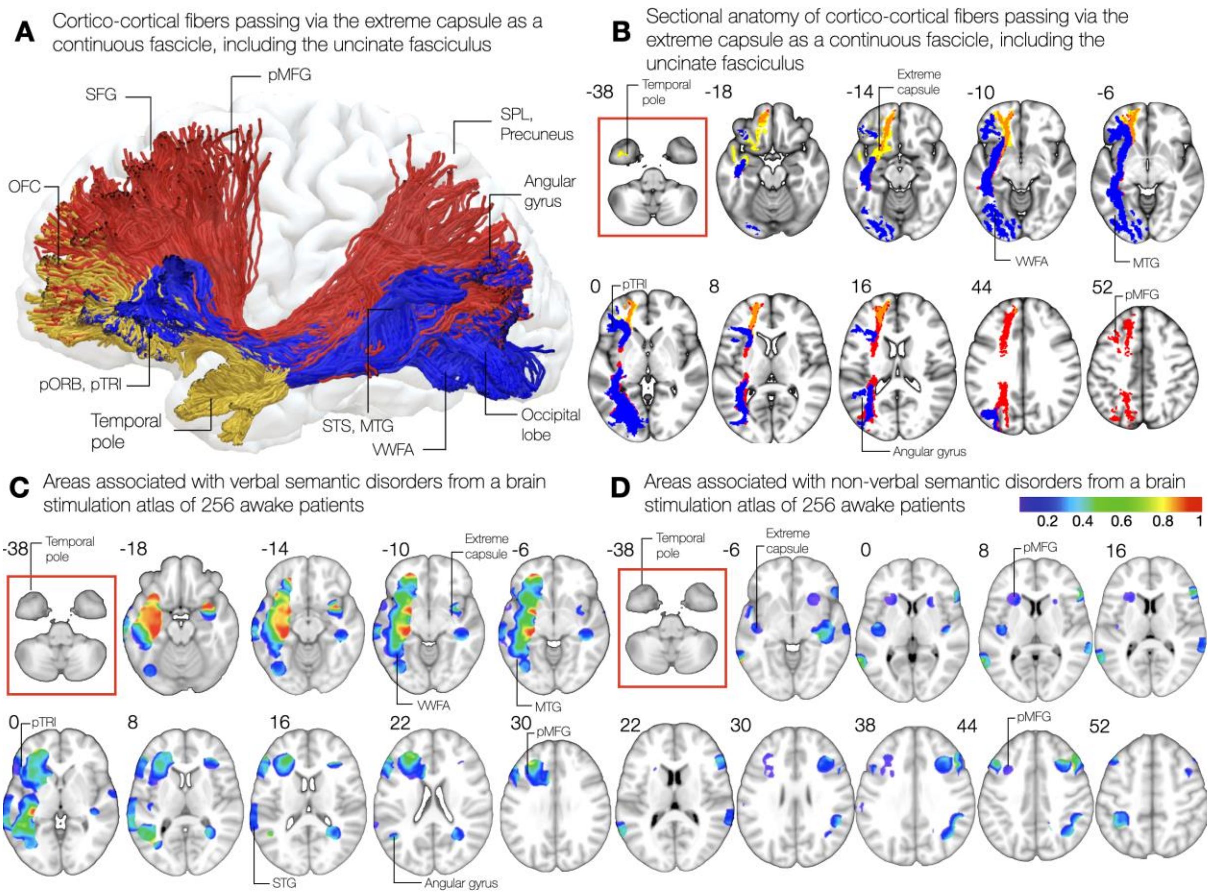

Suppl Fig. 1. Cortico-cortical pathways passing via the extreme capsule. A) 3D reconstruction of cortico-cortical projections passing via the extreme capsule comprising the uncinate fasciculus from a high-resolution diffusion dataset (1.6 mm isotropic) in a healthy subject. B) Sectional anatomy of cortico-cortical projections passing via the extreme capsule. Please note cortical terminations of the uncinate fascicle in the temporal pole (red box) C) Distribution of non-verbal semantic disorders in a stimulation atlas of 256 awake patients showing bilateral distribution of induced errors.[45] Please note no error at the level of the temporal pole (red box), which would include cortical terminations of the uncinate fasciculus. D) Distribution of non-verbal semantic disorders in a stimulation atlas of 256 awake patients showing bilateral distribution of induced errors.[45] Please note no error at the level of the temporal pole (red box), which would include cortical terminations of the uncinate fasciculus. *Yellow: uncinate fasciculus; Red: dorsal component of the IFOF; blue: ventral component of the IFOF; OFC: orbitofrontal cortex; SFG: superior frontal gyrus; pMFG: posterior middle frontal gyrus; pTRI: pars triangularis; pORB: pars orbitalis; STG: superior temporal gyrus; MTG middle temporal gyrus; VWFA: visual word form area; SOG: superior occipital gyrus; MOG: middle occipital gyrus; IOG: inferior occipital gyrus; SPL: superior parietal lobule;*

Ang: Angular gyrus; PreCu: precuneus; pFUSA: posterior fusiform gyrus; STS: superior temporal sulcus

References

1. Tournier JD, Smith R, Raffelt D, et al. MRtrix3: A fast, flexible and open software framework for medical image processing and visualisation. *Neuroimage*. 2019;202. doi:10.1016/j.neuroimage.2019.116137
2. Schilling KG, Blaber J, Huo Y, et al. Synthesized b0 for diffusion distortion correction (Synb0-DisCo). *Magn Reson Imaging*. 2019;64:62-70.
3. Smith SM, Jenkinson M, Woolrich MW, et al. Advances in functional and structural MR image analysis and implementation as FSL. *Neuroimage*. 2004;23(SUPPL. 1):208-219. doi:10.1016/j.neuroimage.2004.07.051
4. Binding LP, Dasgupta D, Taylor PN, et al. Contribution of White Matter Fiber Bundle Damage to Language Change After Surgery for Temporal Lobe Epilepsy. *Neurology*. Published online February 7, 2023:10.1212/WNL.0000000000206862. doi:10.1212/WNL.0000000000206862
5. Dell'Acqua F, Scifo P, Rizzo G, et al. A modified damped Richardson-Lucy algorithm to reduce isotropic background effects in spherical deconvolution. *Neuroimage*. 2010;49(2):1446-1458. doi:10.1016/j.neuroimage.2009.09.033
6. Orset T, Royo J, Santin MD, Pouget P, Thiebaut de Schotten M. A new open, high-resolution, multishell, diffusion-weighted imaging dataset of the living squirrel monkey. *Sci Data*. 2023;10(1):224.
